# Machine Vision-Enabled Octahedral Network Reconstruction and Structural Analysis of Perovskite Quantum Dots

**DOI:** 10.1021/acsnano.5c20211

**Published:** 2026-02-13

**Authors:** Guangyu Du, Haichao Zhang, Tieyuan Bian, Weizhen Wang, Long Hu, Yuxin Liu, Zhen Zhan, Songwei Liu, Yuanzhe Li, Xie He, Chutian Huang, Ying Kong, Lianzheng Hao, Jiawen Wang, Ni Zhou, Bao Tu, Chen Zhu, Jiadong Jaydon Gong, Tom Wu, Jun Yin, Zhouchen Lin, Songhua Cai

**Affiliations:** † Department of Applied Physics, 26680 The Hong Kong Polytechnic University, Kowloon, Hong Kong SAR 999077, China; ‡ AI for Science, Hong Kong Research Institute, 636577 Contemporary Amperex Technology (Hong Kong) Limited (CATL-HK), Hong Kong Science Park, New Territories Hong Kong SAR 999077, China; § School of Advanced Technology, 673407 Xi’an Jiaotong-Liverpool University, Suzhou 215123, China; ∥ School of Materials Science and Engineering, 7800 University of New South Wales, Sydney, NSW 2052, Australia; ⊥ Fujian Science & Technology Innovation Laboratory for Energy Devices of China (CATL 21C Lab), Fujian 352000, China; # Key Laboratory of Multifunctional Nanomaterials and Smart Systems, Suzhou Institute of Nano-Tech and Nano-Bionics, Chinese Academy of Sciences, Suzhou 215123, China; ∇ State Key Laboratory of General Artificial Intelligence, School of Intelligence Science and Technology, 12465 Peking University, Beijing 100871, China

**Keywords:** computer vision, octahedral network, perovskite quantum dots, lattice distortion, scanning transmission electron microscopy

## Abstract

The structural framework of metal-halide perovskites is defined by corner-sharing PbX_6_ octahedra, whose tilts, distortions, and connectivity dictate the phase stability, carrier dynamics, and optoelectronic performance. Despite their pivotal role, direct experimental analysis of octahedral configurations in perovskite quantum dots (QDs) remains elusive due to the lack of robust analytical standards. Here, we introduce a machine vision-enabled approach integrating self-supervised denoising (S2SRED) for noise-sensitive datasets, atomic species classification, and automated reconstruction of the PbX_6_ octahedral network with precise lattice parameter extraction, enabling high-fidelity processing of low-dose scanning transmission electron microscopy (STEM) images. In CsPbI_3_ QDs, we observe reduced PbX_6_ octahedral tilting in the outer unit cells, forming an isotropic core–shell feature. In contrast, mixed-halide CsPbI_3–*x*_Br_*x*_ (*x* = 0.5) QDs show inhomogeneous and anisotropic PbX_6_ octahedral tilting distributions resulting from dopant segregation and impaired phase stability as corroborated by photoluminescence measurements. By standardizing metrics for octahedral and lattice geometries, this method helps establish atomic-scale structure–property links in perovskite nanomaterials.

## Graphical abstract

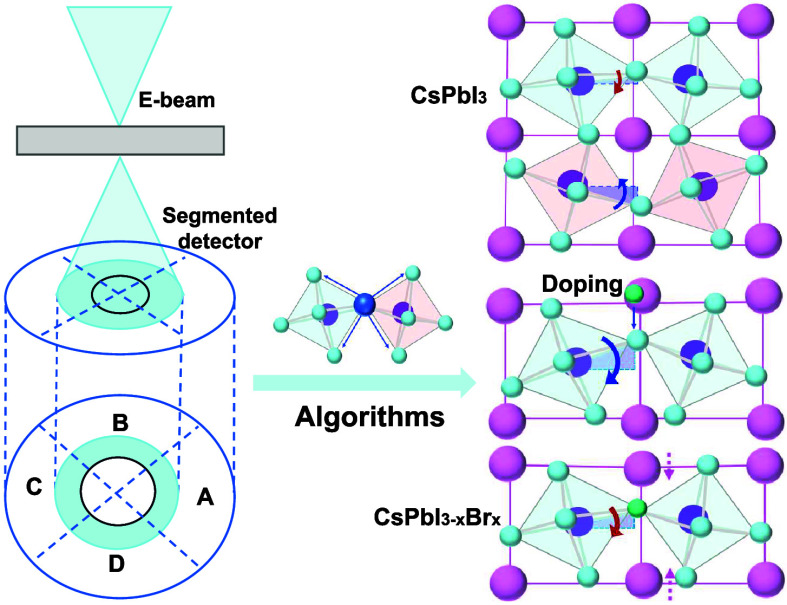

The emergence of halide perovskite ABX_3_ (A: Cs or organic cations; B: Pb, Sn; X = Cl, Br, I), featuring a cubic array of corner-sharing BX_6_ octahedra, with A-site cations occupying the cuboctahedra cavities, has generated significant interest due to their versatile applications in photovoltaics,^1,2^ light-emitting diodes,^3–5^ photodetectors,^6^ and lasers.^7,8^ The distinct crystal structures and chemical components are crucial in determining their optoelectronic properties and phase stabilities,^9,10^ underscoring the significance of structural engineering in realizing high-performance and durable devices.^11^ One of the most important structural features within the perovskite unit cell is the tilting conditions of the BX_6_ octahedra.^12–17^ These tilts, which represent the relative rotations and distortions of the octahedra, are intrinsically correlated to the perovskite phase type (e.g., α, β, and γ phases) and determine materials’ properties, including phase stability, band-edge energies, carrier dynamics, and exciton–phonon coupling.^18,19^ Numerous efforts have been made to tailor the octahedral tilting to optimize phase stabilities and optoelectronic properties, including doping, surface, and interface engineering.^1,4,20^ When it comes to the halide perovskite quantum dots (QDs), nanoscale confinement, high surface-to-volume ratios, and rapid crystallization amplify tilt inhomogeneities and dynamic disorder, profoundly impacting optoelectronic performance and phase resilience. While techniques such as X-ray diffraction (XRD) and grazing-incidence wide-angle X-ray scattering (GIWAXS) reveal average tilt motifs, they lack the spatial resolution to resolve single-unit-cell tilt distributions within individual QDs, precluding a precise determination of structure–property relationships. This gap highlights the need for more precise and direct microscopic characterization and analyzation techniques.^21–25^

Recent advancements in low-dose aberration-corrected scanning transmission electron microscopy (STEM) offer unparalleled insights into the atomic configurations of halide perovskites with minimal beam damage-induced artifacts.^19,26–29^ Compared with the *Z*-contrast high-angle annular dark field (HAADF) STEM imaging, the integrated differential phase contrast (iDPC) STEM technique provides superior sensitivity to light elements with better signal-to-noise ratio (SNR), making iDPC-STEM particularly effective for identifying atomic-level defects and structural distortions and compatible with the Gaussian fitting method for locating atomic positions. Additionally, the iDPC-STEM images do not exhibit thickness-induced artifacts under specific thickness conditions, indicating the robustness for reliable atomic position determination within a defined regime.^30–32^ Currently, leveraging atomic-resolution iDPC-STEM imaging alongside denoising and atom peak-finding processing techniques,^33^ local B-X octahedral distortions within specific regions can be determined to investigate structure variations within metal-halide QDs.^17–19,34,35^ However, achieving reliable atomic-scale interpretation requires exceptionally high-quality images. Although numerous deep learning-based denoising techniques have been developed in recent years, their practical application to atomic-resolution STEM datasets remains severely limited by the scarcity of sufficiently large and high-quality training datasets. Acquiring large volumes of paired noisy/clean images at such resolution is not only experimentally costly and time-consuming but also fundamentally limited by beam-induced damage and intrinsic variability across specimens, thereby restricting the generalizability and robustness of supervised denoising frameworks. Therefore, alternative approaches tailored to atomic-resolution STEM datasets should be pursued.

In addition, current tilt-mapping algorithms exhibit two fundamental limitations when applied across an entire QD. First, the marginal regions near the QD surface often suffer from reduced SNR due to the decreased sample thickness, which leads to inaccuracies and artifacts when using atomic classification and identification methods. Second, the calculation of octahedral tilts in traditional algorithms primarily relied on measuring the tilt of a single X-Pb-X bond within a PbX_6_ octahedron, implicitly assuming a rigid, undistorted framework.^17–19,34,35^ In practice, point defects, composition fluctuations, and surface relaxation induce nonrigid distortions that decouple individual bond tilts from the true octahedral orientation. Consequently, single-angle measurements may underestimate local tilt heterogeneity and misrepresent tilt magnitudes in defect-rich or doped QDs. The compromise in the accuracy and reliability of PbX_6_ tilt maps induced by low SNR at QD margins and oversimplified tilt metrics underscores the need for novel algorithms that integrate robust noise modeling, multibond angular fitting, and full-octahedral geometric reconstruction to achieve high-fidelity octahedral tilting analysis across entire QDs.

In this study, we present a high-accuracy quantitative analysis methodology for examining the structural details of individual halide perovskite QD nanocrystals based on atomic-resolution STEM imaging. For the preprocessing step of iDPC-STEM images taken at low-dose conditions, we employed Self2Self (S2S),^36^ an advanced self-supervised denoising strategy that learns exclusively from the input noisy image, strategically integrating the Regularization-by-Denoising (RED),^37^ named as S2SRED. Compared with existing blind-spot or self-supervised denoisers,^38–40^ S2SRED specifically targets the challenges of low-dose STEM by coupling stochastic self-prediction with a nonlocal RED prior. This hybrid formulation stabilizes single-image optimization and suppresses structural incoherence that conventional Self2Self (S2S) or DIP occasionally produces at marginal regions. In addition, this approach circumvents the constraint imposed by the limited availability of high-quality iDPC-STEM training data while faithfully preserving the subtle structural motifs of QDs. By suppressing noise without external references, it enables highly precise two-dimensional Gaussian fitting and, in turn, accurate localization of atomic columns, even at the marginal regions of QDs. Importantly, the method avoids the hallucination artifacts associated with supervised models trained on synthetic datasets, thereby preserving the weak lattice-frequency components essential for tilt-angle quantification. We also developed a machine vision framework featuring a specialized algorithm termed Spiral Octahedra First Search (SOFS), enabling not only the prospective atomic classification but also the automatic and highly efficient identification of all PbX_6_ octahedra, achieved by linking adjacent halogen atoms in a unified ‘one-pot’ process following atom position detection.^41,42^ Moreover, by fitting all four Pb-X bond angles per octahedron and averaging along two axes, the SOFS drastically reduces tilt errors from local distortions. This enables the direct, quantitative maps of PbX_6_ tilt distributions across entire QDs, alongside simultaneous extraction of bond lengths and angles for comprehensive structural analysis. Applying SOFS to CsPbI_3_ QDs reveals a pronounced core–shell tilt gradient, indicative of reduced octahedral rotation near the particle boundary, demonstrating a potential surface relaxation effect.^17,34,43^ Notably, in CsPbI_3–*x*_Br_*x*_ (*x* = 0.5) nanocrystals doped by Br, the PbX_6_ octahedral tilting becomes more inhomogeneous because of the dopant segregation, evidenced by the shrinkage of X-Pb-X and Cs–Cs bond length, corresponding to the PbX_6_ octahedral tilting decrease. This observation unveils the effect of surface relaxation and dopant segregation on phase inhomogeneity within individual halide perovskite QDs. Molecular dynamics (MD) simulations corroborate these trends, linking surface strain and halide distribution to a local octahedral geometry. Photoluminescence (PL) indicates fast degradation of CsPbI_3–*x*_Br_*x*_ (*x* = 0.5) QDs, potentially attributed to the phase separation accelerated by dopant segregation. Our machine vision-enabled approach provides a promising solution to extract key physical parameters from low-dose STEM observations to gain insights into structural and phase details, highlighting the importance of machine vision methodology in understanding the structure–property relationships in halide perovskites.

## Results and Discussion

### Machine Vision-Enabled Measurements of QD Structural Details

The atomic-resolution HAADF and iDPC-STEM images of CsPbI_3_ and CsPbI_3–*x*_Br_*x*_ QD nanocrystals were obtained by aberration-corrected STEM at room temperature using an electron beam current reduced to 1 pA to mitigate beam-induced damage. The QDs were imaged with orientations close to a major crystallographic zone axis, as evidenced by the projected lattice symmetry and corresponding FFT patterns (Figure S1). The observed atomic column arrangement matches the [001] projection of an orthorhombic perovskite phase (space group *Pbnm*), in agreement with previous reports.^18^ The average lattice parameters of CsPbI_3_ are measured as *a* = 0.911 and *b* = 0.909 nm, while for CsPbI_3–*x*_Br_*x*_ it becomes *a* = 0.897 and *b* = 0.895 nm, suggesting a slight doping effect.

Leveraging the iDPC-STEM imaging exhibiting a better SNR and contrast on both heavier and lighter atoms, we developed a machine vision algorithm combining the denoising by S2SRED, atom position finding, and octahedral identification based on SOFS, achieving the visualization of all PbX_6_ octahedra and corresponding Pb-X bonds within the entire perovskite QD, alongside precise determination of all atomic columns’ coordinates. In this tailored algorithmic pipeline, a single absorptive band-stop filter (ABSF) prefiltered iDPC-STEM image was first denoised using the S2SRED framework (see Figure 1a, Supporting Note 1, and Figures S2–S6). By comparison with ABSF, which suppresses high-frequency components (details in the Methods), S2SRED offers a fundamentally different denoising mechanism. Unlike the purely frequency-selective approach, S2SRED leverages the intrinsic self-similarity of the raw STEM images to infer the underlying lattice signal directly from noisy observations. By optimizing a deep prior constrained by the image itself, it adaptively distinguishes structured atomic features from stochastic fluctuations across all spatial scales, thereby recovering weak lattice details that ABSF would otherwise indiscriminately suppress, while simultaneously achieving an effective reduction of broadband noise (Figures S3–S4). After ABSF processing, although the image resolution does not improve, the SNR of the lattice-frequency spots at the highest resolution is enhanced (Figure 1a). In contrast, although ABSF can attenuate portions of the background noise, it cannot strengthen the weak lattice signals, resulting in a limited improvement of SNR in ABSF-preprocessed images, which may still lead to artifacts in atomic position identification (Figure S5a–g). When these same images were further processed with S2SRED (Figures S3d–f and S4d–f), the denoised results displayed sharp lattice contrast and well-defined atomic columns, recovering subtle structural motifs that ABSF alone failed to preserve, thereby enabling more reliable and precise atomic position determination across the entire nanocrystal (Figures S5 and S6). It is worth noting that, because S2SRED relies on self-supervised blind-spot prediction, we explicitly considered the risk that isolated defects or weak atomic columns could be misinterpreted as noise. Importantly, S2SRED is nongenerative and does not impose lattice periodicity or structural priors; stable reconstruction of atomic columns requires a consistent, correlated signal across multiple blind-spot realizations. As a result, features not supported by reproducible experimental contrast are not artificially introduced during denoising. The denoised output then served as the basis for subpixel localization of atomic centers through 2D Gaussian fitting (Figure 1b and Supporting Note 2). From this refined atomic registry, the SOFS algorithm was initiated from a seed halogen site, I_1_, and recursively expanded to delineate the complete halogen sublattice, extending robustly to the outermost regions of the QDs (see Figure 1c, Supporting Note 3, and Figure S7). To mitigate biases arising from imaging errors, we delineate some regions of the nanoparticle’s outermost layer that cannot be correctly identified (Figure S8) to exclude outside regions most susceptible to background noise.

**Figure 1:**
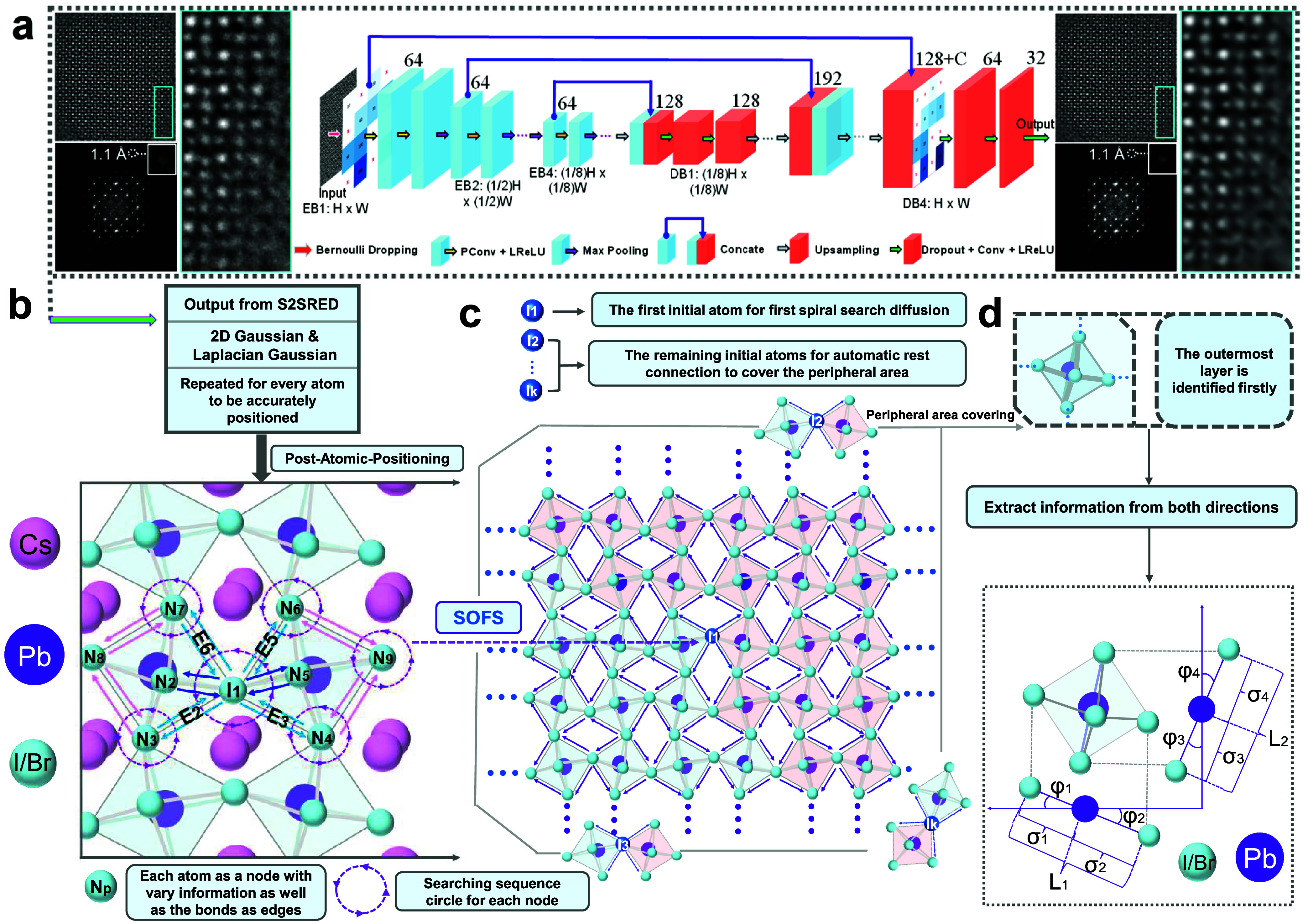
Overall pipeline and algorithmic mechanism of the SOFS framework. (a) Architecture of the employed S2SRED neural network, the FFT of S2SRED-denoised iDPC-STEM image appears sharper and more intense lattice-frequency spots at maximum resolution and magnified view of the cyan box in the original and S2SRED-denoised images, where denoising yields a clearer, more uniform lattice, enabling reliable atomic position extraction. (b) Postatomic-positioning stage: atomic centers extracted from S2SRED outputs using combined Gaussian and Laplacian fitting, with each atom represented as a node carrying positional and intensity attributes, while edges encode interatomic bonds. (c) SOFS algorithm: beginning from the initialized atom I_1_, the algorithm initiates a probabilistically weighted spiral search that links neighboring atoms according to distance, intensity, and angular orientation. At each recursion depth, only the most probable connections are retained, progressively assembling a refined lattice-like framework while discarding spurious background signals. As the search expands toward the particle boundary, additional initial points are introduced to compensate for surface irregularities and ensure complete peripheral coverage. (d) Peripheral-layer coverage strategy: the outermost atoms are recognized first, and bidirectional extraction ensures robust structural representation for further comprehensive analysis.

Within the delineated region of the QD, the octahedral tilting is defined by two angles, θ_1_ and θ_2_, measured along two orthogonal directions. These angles represent the averaged four Pb-X bonds tilt angles, φ*_k_* (*k* = 1, 2, 3, 4) within the PbX_6_ octahedra, as illustrated in Figure 1d. Specifically, φ_1_ and φ_2_ are derived from the horizontal direction, while φ_3_ and φ_4_ correspond to the vertical direction. The angle θ_1_ is computed as the average of φ_1_ and φ_2_, and θ_2_ is determined as the average of φ_3_ and φ_4_, effectively transforming the bond tilts into the octahedral tilting along the respective directions, thus mitigating the measurement error induced by local distortion and imaging artifacts. To extract the comprehensive structural information, we also measured X-Pb-X bond length along two orthogonal directions, denoted as L_1_ and L_2_, which are computed as the summation of Pb-X bond lengths σ*_k_* (*k* = 1, 2, 3, 4) within the PbX_6_ octahedra (Figure 1d). Additionally, we measured the Cs–Cs and Pb–Pb spacings along two orthogonal directions to correlate the lattice distortions, octahedral tilting, and doping effects, denoted as C*_k_* and P*_k_* (*k* = 1, 2). Mapping all the structural parameters enables a multihierarchy interpretation of perovskite structural and compositional information, including phase, strain, and doping conditions, while allowing us to distinguish intrinsic lattice fluctuation from dopant-induced heterogeneity.

### Octahedral Tilting Distribution in Pure CsPbI_3_ QDs

We performed PbX_6_ octahedral tilting and bond length mapping first on CsPbI_3_ QDs without doping. As shown in Figure 2a, the S2SRED-denoised atomic-resolution iDPC-STEM image of an individual CsPbI_3_ QD clearly shows the positions of all atomic columns. The PbX_6_ octahedral tilting distribution was shown in Figure 2b, indicating the tilting angles along horizontal (θ_1_) and vertical (θ_2_) directions, and the averaged tilting angles (θ_3_), respectively. Notably, this PbX_6_ octahedral tilting exhibits a remarkable core–shell-like gradient structure, with an obvious decrease of the octahedral tilting at the outer two or three unit cells of the QD. The Pb-X bond tilting mappings (φ*_k_*) are also given in Figure S9a,b, together with the bonding skeleton that defines the framework of the structure shown in Figure S9c,d. This octahedral tilting angle transition can also be observed from the line profiles extracted from the mapping results (Figures 2d and S10). The averaged value of PbX_6_ octahedral tilting in the outer three unit cells is calculated as 8.92° (θ_1_ and θ_2_ are 8.51°and 9.32°, respectively), and increases to 10.91° in the rest “core” region of the QD (θ_1_ and θ_2_ are 10.27° and 11.55°, respectively). Moreover, the reduction of PbX_6_ octahedral tilting near the QD surface appears to be an isotropic characteristic.

**Figure 2:**
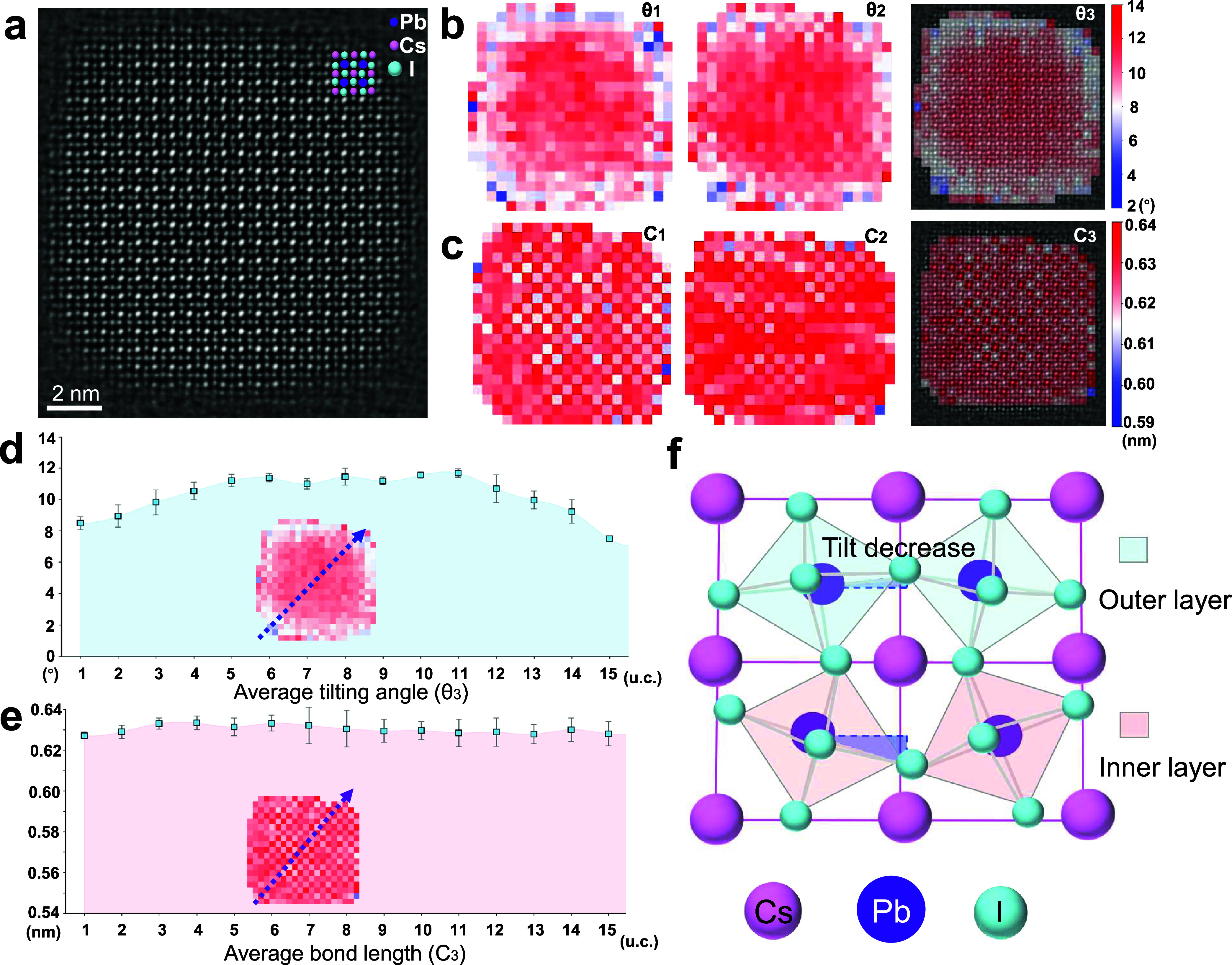
Characterization of a single CsPbI_3_ QD sample. (a) S2SRED-denoised iDPC-STEM image of the quantum dot, providing a high-clarity visualization of its atomic framework. (b) Spatial mappings of the octahedral tilt angles θ_1_ and θ_2_, together with a composite map θ_3_ that integrates their averaged distribution, offering a holistic view of the structural tilting. (c) Mappings of Cs–Cs bond lengths along two orthogonal directions (C_1_ and C_2_) and the combined average distribution (C_3_), derived from the overlap of the two orientations. (d) Line profile of tilt angles obtained along the arrow in the θ_3_ map, extracted by integrating four regions adjacent to the deep blue arrow, each separated by one lattice spacing. (e) Line profile of Cs–Cs bond lengths acquired along the same orientation in the C_3_ map, revealing the directional variation in lattice spacing. (f) Mechanism view of the surface relaxation, which causes the octahedral tilt to decrease in the outer layers.

To determine whether the lattice distortion corresponds to PbX_6_ octahedral tilting, the I–Pb–I spacing and separated Pb–I bond length (σ*_k_* and L_1_, L_2_ in Figure 1d) distribution along horizontal and vertical directions are extracted (Figure S11). Interestingly, these lattice spacings show subtle heterogeneity across the entire QD with the maximal deviations less than 0.02 nm, indicating the structural homogeneity of this QD sample. Moreover, the Cs–Cs and Pb–Pb spacing distributions are analyzed by classifying the Cs and Pb atomic columns through this algorithm. As shown in Figures 2c,e and S12–S14, both the Cs–Cs and Pb–Pb spacing exhibit subtle heterogeneity across the entire QD, indicating an almost strain-free condition. As a result, the decrease in PbX_6_ octahedral tilting at the marginal region cannot be attributed to strain concentration or lattice distortion. Another CsPbI_3_ QD nanocrystal with a smaller size was also analyzed through this methodology (Figures S15–S19), demonstrating a very similar phenomenon. The subtle strain concentration within CsPbI_3_ QDs is also validated by geometric phase analysis (GPA) as shown in Figure S20. The rotation angle of the PbX_6_ octahedra increases from the outer toward the center, which can be explained by the increased surface energy required to form a highly symmetrical structure near the edge^17,34,43^ (Figure 2f). Notably, this surface energy is not large enough to induce obvious edge Cs–Cs distortion. Therefore, in CsPbI_3_, the ligand-coordinated surface stabilizes the halide framework by increasing the structural symmetry through reduced octahedra rotation rather than stretching the outer lattice.^44^

### Visualization of Bromine Doping Effect

In another mixed-halide CsPbI_3–*x*_Br_*x*_ QD sample with Br^–^ doping, a distinct distribution of PbX_6_ octahedral tilting and bond length can be identified (Figure 3a). Rather than appearing as an isotropic “core–shell” like PbX_6_ octahedral tilting character in pure CsPbI_3_ QDs, the distribution of PbX_6_ octahedral tilting becomes more anisotropic with remarkable deviations at localized regions of the CsPbI_3–*x*_Br_*x*_ QD (Figure 3b), implying the influence from Br^–^ doping as both QD nanocrystals have similar sizes (Figures 2a and 3a). Notably, the PbX_6_ octahedral tilting decreases especially at the bottom-left corner of the CsPbI_3–*x*_Br_*x*_ QD (Figures 3b,d and S21 and S22), which cannot solely be attributed to the surface relaxation effect. In particular, the incorporation of Br^–^ will shorten the bond length of Pb–I, leading to a smaller PbX_6_ octahedron, which tends to tilt more slightly. As shown in Figure S23, the average X-Pb-X bond length is around 0.607 nm in this mixed-halide system, smaller than in the pure CsPbI_3_ QDs, around 0.633 nm (Figure S11), confirming the Br^–^ doping-induced Pb-X bond length shrinkage. Interestingly, the X-Pb-X bond length distribution also appears anisotropic within the CsPbI_3–*x*_Br_*x*_ QD, with the X-Pb-X bond length at the bottom-left corner region slightly smaller than the rest of the part (Figure S23), consistent with the decreased PbX_6_ octahedral tilting (Figure 3b). Therefore, the localized decrease of PbX_6_ octahedral tilting and X-Pb-X bond length should result from a higher incorporation degree of Br^–^, which showcases the segregation of Br^–^ dopants. Although localized vacancies or elastic strain could also induce bond-length variations and changes in octahedral tilting, vacancy-related distortions are typically spatially localized, whereas the bond contraction and tilt reduction observed here extend over multiple unit cells. Similarly, pure elastic strain would be expected to produce smoother or symmetric-related lattice distortions, rather than the anisotropic and spatially confined features resolved in our maps. Moreover, the average Cs–Cs spacing is about 0.606 nm within the CsPbI_3–*x*_Br_*x*_ QD with a slight decrease at the bottom-left corner (Figures 3c,e and S24 and S25), smaller than in the pure CsPbI_3_ QDs around 0.631 nm (Figure 2c,e). A slight reduction of Pb–Pb spacing (Figure S26) can be observed at the bottom-left corner with minimum PbX_6_ octahedral tilting, confirming the Br^–^ segregation-induced lattice distortion (Figure 3f).

**Figure 3:**
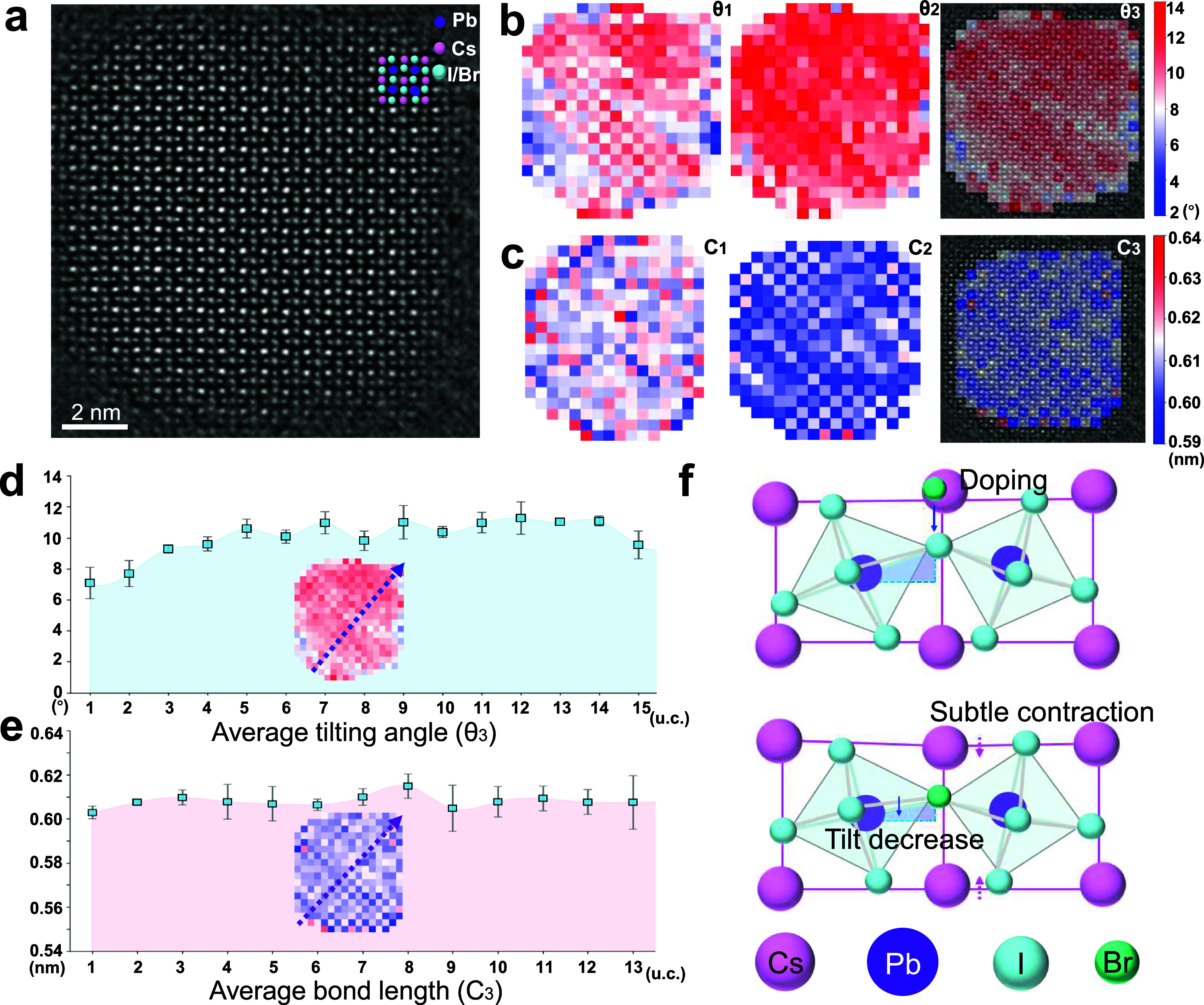
Characterization mappings of a single CsPbI_3‑*x*_Br_*x*_ (*x* = 0.5) QD. (a) S2SRED-denoised iDPC-STEM image of the quantum dot, providing a high-clarity visualization of its atomic framework. (b) Spatial mappings of the octahedral tilt angles θ_1_ and θ_2_, together with a composite map θ_3_ that integrates their averaged distribution, offering a holistic view of the structural tilting. (c) Mappings of Cs–Cs bond lengths along two orthogonal directions (C_1_ and C_2_) and the combined average distribution (C_3_), derived from the overlap of the two orientations. (d) Line profile of tilt angles obtained along the dark-blue arrow in the θ_3_ map, extracted by integrating four regions adjacent to the arrow, each separated by one lattice spacing. (e) Line profile of Cs–Cs bond lengths acquired along the same orientation in the C_3_ map, revealing the directional variation in lattice spacing. (f) Mechanism view of the smaller Br doping-induced lattice contraction.

We then applied this methodology to two additional CsPbI_3–*x*_Br_*x*_ QDs with different sizes, both of which exhibit a similar anisotropic distribution of PbX_6_ octahedral tilting and bond lengths (Figures S27–S36). In a much larger CsPbI_3–*x*_Br_*x*_ QD shown in Figure S27, this algorithm is also effective in analyzing the structural parameters, although the shape of the QD is irregular with more complex margin geometry. The protruding region at the bottom-left corner exhibits a reduced PbX_6_ octahedral tilting (Figures S27b–e and S28) with corresponding shrinkage of X-Pb-X bond length (Figure S29), suggesting a localized Br^–^ dopant segregation at this region. Both the Cs–Cs (Figure S30) and Pb–Pb spacings (Figure S31) exhibit a slight shrinkage in this protruding region. It is worth noting that the PbX_6_ octahedral tilting also tends to decrease in the marginal region of this larger QD nanocrystal, which may result from the coupling effect of surface relaxation. When it comes to another CsPbI_3–*x*_Br_*x*_ QD with a much smaller size (Figure S32), an obvious decrease of PbX_6_ octahedral tilting appears at one- or two-unit cells of the outer left (Figures S32b–e and S33), consistent with the shrinkage of X-Pb-X bond length at the same region (Figure S34). Besides, the Cs–Cs (Figure S35) and Pb–Pb spacing (Figure S36) also show slight shrinkage at the outer left region, implying that the reduced PbX_6_ octahedral tilting should be attributed to the localized Br^–^ dopant segregation. The GPA results are also in strong agreement with our mapping results (Figure S37). As a result, compared to the ligand-assisted surface relaxation effect contributing to the isotropic PbX_6_ octahedral tilting mitigation at the outer shell, halide doping and resultant dopant segregation will lead to more pronounced structural heterogeneity.

### Molecular Dynamics Simulations and Experimental Comparisons

Molecular dynamics (MD) simulations were performed for both CsPbI_3_ and CsPbI_3–*x*_Br_*x*_ QD nanocrystal systems to validate the influence of surface relaxation and dopant segregation on PbX_6_ octahedral tilting distribution. As shown in Figure 4a,b, cubic CsI-terminated perovskite QD models with a size equal to 9.5 nm × 9.5 nm × 9.5 nm were built based on experimentally synthesized QDs (details in Methods). In the case of pure CsPbI_3_ (Figure 4a), as presented in Figure 4c, which is a spatially resolved map of the octahedral tilting angles, the simulations revealed a distinct “core–shell” structural feature with regions classified by the tilt angles of Pb–I bonds due to surface relaxation from under-coordinated Pb–I octahedra at the CsI-terminated surfaces. It is important to note that Figure 4c is not merely a single snapshot but a time-averaged map calculated over the MD trajectory. This averaging process removes transient thermal fluctuations, revealing the intrinsic structural equilibrium of the QD. This direct visualization confirms that the reduced octahedral tilting is not randomly distributed but is spatially localized at the QD surface, providing strong qualitative agreement with the core–shell feature observed in experiments. Specifically, Figure 4d illustrates that the tilt angles in the inner core region are predominantly distributed around 9°, with a peak distribution intensity exceeding 0.2 (Figure S38d). In contrast, the outer shell region exhibits a more uniform angular distribution over a reduced angle range, corroborating the experimental analysis. Furthermore, the calculated PbX_6_ octahedral tilt angles were analyzed across three orthogonal projections to eliminate the potential directional bias. Figures S38 and S39 demonstrate consistent “core–shell” structural tendencies across all projections, strengthening the conclusion that this feature is intrinsic to QD nanocrystals rather than an artifact of a particular viewing angle, which is predominantly affected by surface relaxation. Besides, to confirm the robustness of the size-dependent behavior, additional MD simulations were performed on a smaller CsPbI_3_ QD model (approximately 4 nm in size) and compared the octahedral tilting distribution directly with the larger QD model (approximately 9.5 nm), as shown in Figure 4a. In both models, we defined the shell region as the outermost layer of PbI_6_ octahedra, with the remaining interior defined as the core. The tilt angle distributions for the shell regions of both the small (blue dashed) and large (blue solid) QDs are nearly identical (Figure S38c), suggesting that the magnitude of octahedral relaxation induced by the surface ligands is an intrinsic local effect largely independent of the total particle volume. Additionally, the simulated Cs–Cs spacing (Figure 4f) indicates minimal variation across the QD nanocrystal, consistent with experimental analysis.

**Figure 4:**
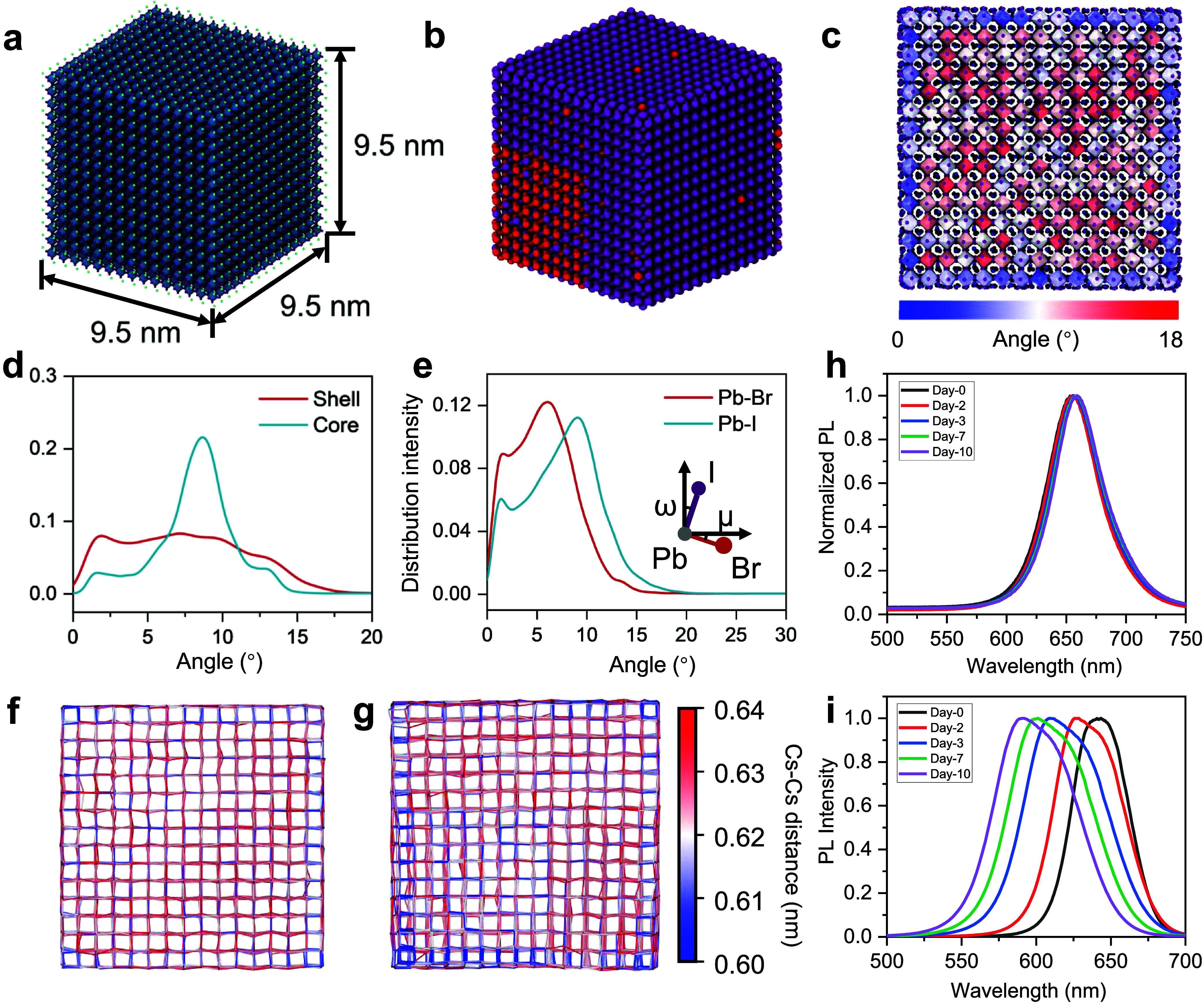
MD simulation and experimental results of CsPbI_3_ and CsPbI_3‑*x*_Br_*x*_ (*x* = 0.5) QD. Schematics of (a) CsPbI_3_ QD and (b) CsPbI_3–*x*_Br_*x*_ QD. In the CsPbI_3–*x*_Br_*x*_ schematic, red spheres represent Br atoms and purple spheres represent I atoms. Pb and Cs atoms are omitted for clarity. (c) Schematic illustration of the core–shell regions in a CsPbI_3_ QD with the simulation mapping of Pb–I bond distortion angles. (d) Distribution of Pb–I bond distortion angles in both shell and core regions in CsPbI_3_. (e) Distribution of distortion angles for the Pb–Br and Pb–I bonds in CsPbI_3–*x*_Br_*x*_. The inset diagram illustrates the calculated distortion angles. Simulation mapping of Cs–Cs interatomic distances in (f) CsPbI_3_ and (g) CsPbI_3–*x*_Br_*x*_. (h) Normalized PL spectra of the CsPbI_3_ QD sample stored for different periods of time. (i) Normalized PL spectra of CsPbI_3–*x*_Br_*x*_ QD sample stored for the same periods of time, indicating an obvious peak shift and broadening.

For the CsPbI_3–*x*_Br_*x*_ QD case, a pronounced Br–I heterogeneity is constructed to represent the dopant segregation, as shown in Figures 4b and S40. By comparison of the tilting of Pb–I and Pb–Br across three projections in the 3D model to eliminate potential biases, a significant variation in their distribution intensity peaks can be identified. Specifically, the peak for Pb–Br tilt angles is approximately 5.5°, while that for Pb–I tilt angles is around 9.5°, indicating a remarkable reduction of Pb–Br tilt angles compared to Pb–I (Figures 4e and S41). The Cs–Cs spacing (Figure 4g) and Pb–Pb spacing (Figure S42) reveal that an obvious shrinkage corresponds to the dopant segregation in CsPbI_3–*x*_Br_*x*_. The extent of reduction in the Cs–Cs spacing can be attributed to the structural adjustments for accommodating Br^–^ with a smaller ionic radius compared to I^–^. Additionally, the difference in bonding strength between the Pb–Br and Pb–I bonds also contributes to the lattice spacing reduction. The calculated average Cs–Cs and Pb–Pb spacing is 0.621 and 0.627 nm in CsPbI_3_, while for CsPbI_3–*x*_Br_*x*_ it is 0.603 and 0.618 nm, respectively (Figure S43). The variations in PbX_6_ octahedral tilting and Cs–Cs spacing in this theoretical analysis strongly suggest that the experimentally observed localized PbX_6_ octahedral tilting mitigation and lattice spacing shrinkage should result from Br^–^ segregation in mixed-halide perovskite QDs.

The divergence in PbX_6_ octahedral tilting may underpin distinct phase dynamics. To assess the stability of pure CsPbI_3_ and mixed-halide CsPbI_3–*x*_Br_*x*_ QD samples, time-series photoluminescence (PL) measurements were conducted. As illustrated in Figure 4h, the PL peak of the CsPbI_3_ QD sample displays minimal displacement and negligible shape variation after 10 days of storage, suggesting only a subtle phase transition. This enhanced phase stability can be ascribed to the core–shell-like gradient in PbX_6_ octahedral tilting, which yields a more symmetric and robust outer layer. In contrast, for the CsPbI_3–*x*_Br_*x*_ QD sample, Figure 4i reveals a progressive blue shift of the PL peak over the same period, accompanied by early-stage broadening and asymmetry relative to the initial spectrum. Notably, upon degradation, the PL spectra, particularly for CsPbI_3–*x*_Br_*x*_ QDs, exhibit pronounced broadening accompanied by spectral asymmetry and shoulder-like features, indicating that the PL evolution cannot be explained by a single homogeneous broadening mechanism. Instead, this behavior is consistent with the coexistence of multiple emissive contributions that evolve over time. Such contributions likely arise from local compositional inhomogeneity or phase variations induced by Br^–^ segregation, in agreement with our atomic-resolution STEM results showing spatially heterogeneous PbX_6_ octahedral tilting and bond-length distributions. Regions with reduced octahedral tilting and shorter Pb-X bonds are expected to possess larger effective bandgaps, giving rise to higher-energy emission components. As degradation proceeds, these structurally distinct domains may undergo divergent phase dynamics, further enhancing PL broadening and peak splitting and thereby compromising the overall sample stability.

## Conclusion and Outlook

While the halide doping can effectively modulate the optoelectronic properties of perovskite QDs through mitigating PbX_6_ octahedral tilting, the segregation of halide dopants is dominant rather than an ideal uniform doping, which may harm the phase stability due to structural heterogeneity (Figure 4h,i). Therefore, characterizing the dopant distribution within QDs is necessary to evaluate the doping effects. However, the contrast between Br and I is very close in both HAADF- and iDPC-STEM images, as shown in the simulation results (Supporting Note 4 and Figure S44). Besides, the spectroscopic techniques, including X-ray energy dispersive spectroscopy (EDS) and electron energy loss spectroscopy (EELS), always require a very high electron dose to realize atomic-level elemental mapping, making them inapplicable to distinguish the dopant position within halide perovskite QDs. As evidenced by this machine vision approach, the segregation of Br^–^ dopants can be determined by the reduction in PbX_6_ octahedral tilting and corresponding bond-length shrinkage. This provides a possible solution to analyze the structural and phase heterogeneity in mixed-halide perovskite systems through atomic-resolution STEM imaging, which is meaningful in unveiling the underlying structure–property relationships. Furthermore, combining this machine vision-enabled STEM datasets analysis with *in situ* characterizations, the dynamic evolutions of nanoscale phase transitions under external stimuli can be revealed at a time resolution of up to seconds.^18,19,34,35^

Our computational analysis corroborates the experimental findings, revealing a spatially heterogeneous pattern of octahedral distortion within the Br-doped perovskite lattice. In Br-enriched domains, the octahedral tilt angles decrease markedly compared with the surrounding matrix, whereas the accompanying contraction of the Cs–Cs and Pb–Pb sublattice spacings is smaller than initially anticipated, which could be attributed to the fact that the framework relieves part of the local strain through angular tilting rather than pronounced bond-length shortening. A slight overall reduction in the average Cs–Cs and Pb–Pb spacings nevertheless persists, consistent with net lattice compaction associated with the enhanced tilts. The incorporation of Br thus introduces pronounced local heterogeneity that disrupts the global symmetry of the crystal lattice. Such segregation-induced symmetry breaking and tilt distortion may serve as preferential nucleation sites for grain-boundary development or even drive solid-state phase transformations. Elucidating how these locally confined distortions influence collective structural transitions, such as ferroelectric phase switching, will be crucial for understanding and ultimately controlling the macroscopic behavior of doped perovskites.

In conclusion, we developed a novel machine vision algorithm for high-efficiency processing of atomic-resolution STEM images, enabling the identification of different atoms and precise measurement of key structural parameters, including the PbX_6_ octahedral tilting, bond length, and lattice spacing throughout the entire perovskite QD nanocrystals. Particularly, the tilting angle of PbX_6_ octahedra is measured from two orthogonal directions, minimizing potential artifacts resulting from structural distortion or imaging conditions. In the pure CsPbI_3_ QD, we uncovered an isotropic reduction of PbX_6_ octahedral tilting at the outer region, potentially driven by ligand-assisted surface relaxation, forming a “core–shell” like feature, suggesting that organic–inorganic interfacial interactions play a significant role in structural configuration. In contrast, in mixed-halide CsPbI_3–*x*_Br_*x*_ QD nanocrystal, the segregation of Br^–^ dopant leads to localized PbX_6_ octahedral tilting alleviation, causing severe structural heterogeneity, which may be detrimental to phase stability. The development of machine vision-assisted atomic-scale structural analysis offers a potential solution for quantifying structural parameters in halide perovskite QDs. While an ultimate end-to-end deep learning framework that directly infers atomic positions and structural parameters from raw low-dose STEM data would be highly desirable, such approaches currently face practical challenges related to training data availability, interpretability, and quantitative reliability under low-dose conditions.^45–48^ By combining self-supervised denoising with established model-based fitting and physics-guided atom classification, the present workflow achieves high accuracy and robustness for complete structural parameter extraction in beam-sensitive halide perovskite QDs. By enabling spatially resolved mapping of PbX_6_ octahedral tilting angles, bond lengths, and lattice parameters across entire nanocrystals, this methodology provides unprecedented insights into nanoscale structural heterogeneity that was previously obscured by ensemble-averaged techniques. The correlation of spatially resolved PbX_6_ octahedral tilting and superficial and compositional parameters will be enlightening for designing structural engineering strategies to modulate the optoelectronic properties and improve the stability of perovskite semiconductors. Moreover, the methodology can be extended to other atomic-resolution phase-sensitive imaging techniques, as recent advances in low-dose phase-imaging methods, including exit-wave-reconstructed TEM and 4D-STEM ptychography, show promise in recovering atomic-scale information even for beam-sensitive materials.^49–52^

## Experimental Section

### CsPbI_3_ and CsPbI_3–*x*_Br_*x*_ (*x* = 0.5) QD Synthesis

Both types of QDs were synthesized according to our previous recipe.^53^ 0.2 g Cs_2_CO_3_ was mixed with 10 mL ODE and 0.8 mL OA in a three-neck flask, and then this mixture was completely dissolved under stirring and vacuum at 120 °C for 1 h, named as Cs-OA precursor. CsPbI_3_ and CsPbI_3‑*x*_Br_*x*_ QDs were synthesized separately using two three-neck flasks. All conditions were exactly the same except for the injection temperature and the ratio of PbI_2_ and PbBr_2_ for both QD syntheses. For CsPbI_3_ QD synthesis, 0.5 g of PbI_2_, 2.5 mL of oleic acid (OA), and 25 mL of octadecene (ODE) were added into a 100 mL three-neck flask and vacuum pumped under continuous stirring at 100 °C for 1 h. Then, 2.5 mL of oleylamine (OLA) was injected into the flask. After PbI_2_ was completely dissolved, the temperature increased to 150 °C under N_2_ flow protection. Two mL of Cs-OA precursor was swiftly injected into the reaction mixture, and the solution was quenched by an ice bath after 10 s. For CsPbI_3‑*x*_Br_*x*_ QD synthesis, 0.368 g PbI_2_ and 0.07 g PbBr_2_ were loaded into the mixture of 25 mL ODE and 2.5 mL OA, and all other conditions were identical except the injection temperature at 170 °C. Both types of QD solutions were evenly divided into three 50 mL centrifugation tubes, and then anhydrous methyl acetate was added to the tubes with a volume ratio of 1:2 (QD solution: methyl acetate). Subsequently, QD precipitates were obtained by centrifugation at a speed of 8000 rpm for 3 min. All QD precipitates in 3 tubes were redispersed with 3 mL of hexane and then precipitated by adding 4.5 mL of methyl acetate and centrifuged again at 8000 rpm for 3 min. Finally, the purified QDs were dissolved in octane for further characterization.

#### Steady-State Photoluminescence Measurements

Steady-state PL measurements over storage time were conducted at room temperature using a custom laser PL spectroscopy system, Crystal Laser, Model BLC-050–405, with the excitation wavelength being 532 nm.

### Low-Dose STEM Characterization

Low-dose STEM observations were performed on an aberration-corrected microscope equipped with a field emission gun at 300 kV (Spectra 300, Thermo Fisher). A convergence half-angle of 29.9 mrad was used, and the collection half-angle of the HAADF-STEM detector ranged from 57 to 200 mrad for atomic-resolution imaging. The beam current of the electron probe was reduced to 1 pA, and the dwell time for each pixel was 1 μs. The average frame size is around 34.5 × 34.5 nm^2^ and 2048 × 2048 pixels for the atomic-resolution STEM image. Thus, the average dose rate can be estimated as around 53 e·Å^–2^·s^–1^ for acquiring each atomic-resolution image. Then all the raw STEM images were prefiltered with ABSF. The filter was run with a step size of 5 pixels, a frequency increment (Δ) of 2.0% per step, 20 iterative cycles, and a bandwidth of 3 pixels combined with a 0.5-pixel soft-edge bandwidth to reduce ringing at the stop-band edges. The main stop-bands were centered at spatial frequencies between 0.18 and 0.24 Å^–1^, corresponding to the dominant scan-line noise peaks in the Fourier spectrum.

### Image Predenoising

The network (Figure 1a) is an encoder–decoder tailored for single-image S2S training with five encoder blocks (EB1-EB5) and four decoder blocks (DB1-DB4). EB1-EB4 each apply partial convolution (3 × 3, stride 1, same padding) + LReLU (slope 0.1) + 2 × 2 max-pooling (stride 2), while EB5 omits pooling; all encoder stages use a constant feature width *F* = 64, which stabilizes single-image optimization and keeps skip-connection statistics consistent across scales. The decoder upsamples by a factor of 2 at every stage (nearest-neighbor or bilinear), concatenates with the coscale encoder feature (skip), then performs two Dropout-Conv-LReLU layers per block. Concretely, DB1 (1/8 scale) upsamples EB5′s 64-ch features, concatenates with EB4′s 64-ch skip (128 channels pending projection), and projects to 128 channels for both convolutions. DB2 (1/4) repeats the pattern, receiving 128-ch input from DB1, concatenating EB3′s 64-ch skip (128 + 64 = 192 preconv), then projecting back to 128 for its two convolutions. DB3 (1/2) does the same with EB2′s skip (192 → 128). DB4 (H × W) upsamples DB3′s 128-ch features, concatenates EB1′s 64-ch skip (192 preconv), and performs a three-layer taper 128 → 64 → 32 → C, where C is the image channel count; only this last block changes channel dimensionality, mirroring the canonical S2S design. All decoder convolutions use element-wise dropout (*p* ≈ 0.3), which remains enabled at inference to realize a Monte Carlo ensemble; typical test-time aggregation averages *N* ≈ 50–100 stochastic forward passes. Furthermore, we adopt an alternating optimization strategy inspired by ADMM to integrate RED into the S2S framework (Supporting Note 1). The noise standard deviation parameter of BM3D, which functions as RED, is set to σ = 15/255, with RED regularization λ = 0.2, and ADMM penalty μ = 1.0. With parallelization across multiple images, the implementation requires approximately 2.5 h per 1024 × 1024 image on average using an NVIDIA GeForce RTX 3060 Laptop GPU.

### Image Processing for Octahedral Analysis

Accurate octahedral tilt angle quantification was ensured by aligning the *x*- and *y*-scan directions with orthogonality preserved to within 1°. The search for atomic centers was subsequently carried out with the aid of the Atomap package,^42^ candidate atomic positions were initially detected through local maxima searching within a 7 × 7 pixel neighborhood, and further refined using a center-of-mass (CoM) calculation within a circular aperture of radius 3 pixels. Nonlinear least-squares optimization was performed using the Levenberg–Marquardt algorithm with convergence tolerance 10^–6^, yielding subpixel precision typically better than 0.005 nm. To ensure robustness, fits with residual errors exceeding 8% of the local peak intensity were rejected and reinitialized with perturbed CoM estimates (Supporting Note 5). When propagated to the calculation of Pb-X bond lengths and PbX_6_ octahedral tilt angles, this positional uncertainty corresponds to bond-length uncertainties of a few picometers and angular uncertainties generally below 0.5–1°. For heavy-element sublattices (Pb), atom-specific thresholds were applied such that only peaks exceeding 1.5 times the global average amplitude were retained, enabling Pb atom recognition and the selective Pb–Pb distance analysis. Then, for the halogen (I/Br) atom recognition, we use the proposed SOFS algorithm (Supporting Note 3). For each depth level, eight candidate neighbors were identified, of which the four highest-probability nodes were retained, ensuring recursive expansion of the network with a branching factor *B* = 4 and depth limited to α = 9. Surface distortions and spurious background nodes were handled by multiseed initialization: once the expansion from a seed N_1_ converged to an irregular contour, a new seed N_2_ was randomly selected near the boundary, and the recursion continued until full coverage was achieved. Pruning heuristics and periphery-restricted searches reduced the runtime significantly, with average processing times of approximately 16 min for 1024 × 1024 images on an NVIDIA GeForce RTX 3060 Laptop GPU. Furthermore, once the Pb and halogen (I/Br) atoms are identified and subtracted, the remaining Cs sites can be unambiguously resolved. This enables reliable “one-pot” atomic classification in CsPbX_3_ (X = I/Br) QDs iDPC-STEM images without the aid of complementary HAADF images, despite the similar Gaussian-like peak profiles of Cs and halogen atoms, and facilitates subsequent analysis of the Cs–Cs lattice. All angle and lattice determinations were performed by using the calculation method illustrated in Figure 1d.

### Molecular Dynamic Simulations

We performed MD simulations of CsPbI_3_, CsPbBr_3_, and CsPbI_3‑*x*_Br_*x*_ using the reactive force field ReaxFF. ReaxFF combines bond orders with polarizable charges to describe both reactive and nonreactive atomic interactions, allowing for accurate modeling of covalent and electrostatic interactions in different materials. The contributions to the ReaxFF potential can be summarized as follows

$$E_{\mathrm{system}}=E_{\mathrm{bond}}+E_{\mathrm{vaW}}+E_{\mathrm{tors}}+E_{\mathrm{over}}+E_{\mathrm{under}}+E_{\mathrm{lp}}+E_{\mathrm{coul}}+E_{\mathrm{val}}+E_{\mathrm{conj}}+E_{\mathrm{pen}}$$

where the energy terms include, following the order above, the short-range bond energy, van der Waals energy, torsion energy, overcoordination energy, undercoordination energy, long-range electron pairs energy, Coulomb potential energy, valence angle energy, conjugation energy, and penalty energy. The ReaxFF parameters for Cs, Pb, I, and Br atoms employed in the present study are those in a previous work.^54^ The ReaxFF potential was employed for MD simulations to investigate the dynamic behavior of bulk CsPbI_3_ and mixed-halide CsPbI_3‑*x*_Br_*x*_ perovskites.^44,54,55^

All the MD simulations were performed by the large-scale atomic/molecular massively parallel simulator (LAMMPS).^56^ The temperature *T* was controlled by the Nosé-Hoover thermostat.^57,58^ To simulate isolated quantum dots, no periodic boundary conditions were applied in any of the *x*-, *y*-, or *z* directions in the simulations. Cubic CsPbI_3_ and CsPbI_3‑*x*_Br_*x*_ models were centered in a 20 nm simulation box. CsPbI_3‑*x*_Br_*x*_ model was constructed with ATOMSK software by replacing 1700 corner-site I atoms with Br, followed by randomly replacing an additional 100 I atoms at noncorner sites to enhance overall structural stability.^59^ Each simulation was conducted for 2 ns at 300 K with a time step of 1 fs. All visualizations were performed using OVITO software.^60^

## Supplementary Material



## Data Availability

The authors declare that data supporting the findings of this study are available within the paper and its Supporting Information files. The experiment raw data of this study are available from the corresponding author upon reasonable request.

## Notes

Details of the augmented Self2Self denoising framework with RED regularization and optimization strategy (Supporting Note 1; Figures S2–S4); two-dimensional Gaussian fitting for atomic column localization and robustness comparison (Supporting Note 2; Figures S5–S6); Spiral Octahedra First Search (SOFS) algorithm for automated halide sublattice reconstruction (Supporting Note 3; Figures S7–S8); validation of imaging geometry, specimen thickness, and atomic position fidelity in HAADF- and iDPC-STEM (Supporting Note 5; Figure S1); atomic-scale structural analyses of pure CsPbI_3_ and mixed-halide CsPbI_3‑*x*_Br_*x*_ (*x* = 0.5) quantum dots, including octahedral tilt, bond-length, and Cs–Cs/Pb-Pb sublattice mappings across multiple samples (Figures S9–S37); molecular dynamics simulations and multislice STEM simulations elucidating size-dependent structural relaxation, halide segregation effects, and limited I/Br contrast in HAADF- and iDPC-STEM (Supporting Note 4; Figures S38–S44) (PDF)
