# Supplementary material for: Machine Vision-Enabled Octahedral Network Reconstruction and Structural Analysis of Perovskite Quantum Dots
Source: ACS Nano. 2026 Feb 13;20(7):6125–37. doi: 10.1021/acsnano.5c20211 (PMC12947740; doi:10.1021/acsnano.5c20211)
Supplement: Supplementary file 1 [file nn5c20211_si_001.pdf]

Supplementary Information

Machine Vision-Enabled Octahedral Network Reconstruction and Structural Analysis of Perovskite Quantum Dots

Guangyu Du[1,2,8], Haichao Zhang[3,8], Tieyuan Bian[1,8], Weizhen Wang[1,8], Long Hu[4,8], Yuxin Liu[1], Zhen Zhan[1], Songwei Liu[2], Yuanzhe Li[2], Xie He[2], Chutian Huang[2], Ying Kong[5], Lianzheng Hao[1], Jiawen Wang[2], Ni Zhou[5], Bao Tu[5], Chen Zhu[5], Jiadong (Jaydon) Gong[2], Tom Wu[1,*], Jun Yin[1,*], Zhouchen Lin[6,*], Songhua Cai[1,*]

[1]Department of Applied Physics, The Hong Kong Polytechnic University, Hong Kong SAR 999077, China.

[2]AI for Science, Hong Kong Research Institute, Contemporary Amperex Technology (Hong Kong) Limited (CATL-HK), Hong Kong Science Park, Hong Kong SAR 999077, China.

[3]School of Advanced Technology, Xi'an Jiaotong-Liverpool University, Suzhou 215123, China.

[4]School of Materials Science and Engineering, University of New South Wales, Sydney, NSW 2052, Australia.

[5]Fujian Science & Technology Innovation Laboratory for Energy Devices of China (CATL 21C Lab), Fujian 352000, China

[6]Key Laboratory of Multifunctional Nanomaterials and Smart Systems, Suzhou Institute of Nano-Tech and Nano-Bionics, Chinese Academy of Sciences, Suzhou 215123, China.

[7]State Key Laboratory of General Artificial Intelligence, School of Intelligence Science and Technology, Peking University, Beijing 100871, China.

[8] These authors have contributed equally: Guangyu Du, Haichao Zhang, Tieyuan Bian, Weizhen Wang and Long Hu.

*Correspondence should be addressed to: tom-tao.wu@polyu.edu.hk; jun.yin@polyu.edu.hk; zlin@pku.edu.cn; songhua.cai@polyu.edu.hk

This PDF file includes

Supplementary Notes 1 to 5

Figure S1 to S44

Supplementary Notes

Supplementary Note 1: Mechanism of An Augmented Self2Self Framework With Denoiser-Driven Regularization

Self2Self (S2S) frames single-image denoising as stochastic self-prediction with explicit variance control. Given a noisy observation $y = x + \varepsilon$, draw an element-wise Bernoulli mask r ~ Bern(p) and form a complementary pair $\tilde{y} = r \odot y$ (input) and $\bar{y} = (1 - r) \odot y$ (targets only on hidden pixels), where p denotes the probability a pixel is visible to the network during training, and then 1-p represents the probability a pixel is hidden and must be predicted. The network G_{ϕ} is trained by minimizing the masked MSE:

$$J(\phi) = \sum_m ||(1 - r_m) \odot (G_{\phi}(\tilde{y}) - y)||_2^2 .$$

which prevents the identity map and, under independent zero-mean noise, has expectation:

$$E_{\varepsilon} J(\phi) = \sum_m ||(1 - r_m) \odot (G_{\phi}(\tilde{y}) - y)||_2^2 + \text{const.}$$

indicating that optimization implicitly targets the clean image up to an additive noise term. Interpreting G_{ϕ} as a Bayes estimator with MSE = bias^2 + var, S2S reduces estimator variance by Monte-Carlo dropout: during both training and inference, apply Bernoulli dropout to the configured layers to instantiate a set of perturbed models $\{G_{\phi_n}\}$; at test time, optionally re-mask the input with fresh r_n and average the stochastic predictions:

$$\hat{x} = \frac{1}{N}\sum_{n=1}^{N} G_{\phi_n}(r_n \odot y),$$

which exploits the approximate independence across draws to attenuate variance. Practically, the encoder uses partial convolutions to re-normalize receptive fields on sparsely observed inputs, while the decoder's convolutional blocks are equipped with dropout; the blind-spot effect arises from training on $\tilde{y}$ (hidden pixels) rather than on $\tilde{y}$ itself, and the ensemble average supplies the missing variance reduction that single-image blind-spot methods lack.

Building on the stochastic self-prediction of S2S, we strategically augment the objective with an explicit Regularization-by-Denoising (RED) prior $\rho(\cdot)$ to stabilize training and better preserve nonlocal structure. Concretely, let G_{ϕ} be the S2S network trained with Bernoulli masking on $\tilde{y}$, and introduce an auxiliary variable x together with the RED functional:

$$\rho(x) = \frac{1}{2} x^{\top}(x - f(x)),$$

where $f(\cdot)$ denotes a chosen denoiser (here we employed BM3D). We then solve

$$min_{(\phi,x)} J_{(S2S)}(\phi;\tilde{y},y) + \lambda\rho(x),$$
$$\text{s.t. } x = G_{\phi}(\tilde{y}).$$

using an ADMM splitting that avoids differentiating through f. Each iteration alternates (i) a network-update that minimizes the masked MSE plus a quadratic proximity to the current x (preserving S2S's dropout-based variance control), and (ii) a RED-update of x via the fixed-point step:

$$x \leftarrow (\lambda f(x) + \mu [G_{\phi}(\tilde{y}) + u])/(\lambda + \mu),$$

leveraging $\nabla\rho(x) = x - f(x)$ to enforce denoiser-consistent structure, where λ and μ denote weight on the RED regularization and ADMM penalty ensuring consistency between x and network output, respectively. Finally, the dual variable is refreshed, and multiple stochastic forward passes are averaged at test time. This S2S+RED hybrid preserves SNR-driven precision from self-supervision while injecting a powerful nonlocal prior, thereby improving fidelity in delicate textures and marginal regions without sacrificing the single-image training regime.

Supplementary Note 2: Mechanism of Atomic Position Finding With 2-dimensional Gaussian Fitting

The goal of this phase is to meticulously unveil the precise positions and unique shapes of each atomic column nestled within these images. The endeavor of fitting a single sublattice unfolds in a sequence of three critical steps, each orchestrated to refine and reveal atomic intricacies with scientific elegance. First, one must locate the positions of each atomic column selected for examination, marking them like stars in a microcosmic constellation. This initial survey lays the groundwork for deeper refinement. Next, through the calculated technique of center-of-mass (CoM) refinement, these preliminary positions are honed, drawing each atomic coordinate ever closer to its idealized center. This ensures that, when the final fitting occurs, the 2-D Gaussian function can seamlessly embrace each atomic column's structure. Finally, the fitting itself emerges. Each atomic column is modeled through a 2-D elliptical Gaussian function, $I(x,y)$, capturing its spatial and intensity profile:

$$I(x,y) = I_0 + Aexp\left[-(a(x-x_0)^2 - 2b(x-x_0)(y-y_0) + c(y-y_0)^2)\right].$$

here, I_0 represents the background intensity, and A the amplitude, defining the peak brightness. The parameters x_0 and y_0 designate the atomic column's central coordinates. The constants $a,\ b$ and c are crafted from the rotation and variance elements that dictate the elliptical shape, each encoding the interplay between spatial orientation and the natural deviations.

Supplementary Note 3: Mechanism of the Spiral Octahedra First Search Algorithm

Following the identification of atomic positions using 2D Gaussian fitting, all detected peaks representing atoms above the threshold are displayed across the entire image, unclassified. These dots encompass not only the atoms within the nanoparticle but also some inevitable error points in the surrounding background substrate. Given that the entire particle is the subject of interest, these external dots are often unavoidable. Moreover, isolating only the halogen atoms typically requires the integration of both dark-field and bright-field electron microscopy images, a process aimed at subtracting heavier atoms. However, this refinement step involves numerous cumbersome procedures. Our SOFS algorithm elegantly overcomes these dual challenges, offering a streamlined and efficient solution that both filters out extraneous data points and simplifies the process of atom classification, ensuring a precise visualization of the halogen atom network within the nanoparticle. The process commences with the construction of a sophisticated, multi-attribute atomic connectivity network, where each atom in the image is represented as a node, and the distance between two atoms is defined as the edge connecting them. Each node in this network encodes not only its two-dimensional Cartesian coordinates but also additional critical information, such as intensity and atom-specific roles, essential for precisely weighting the connections within the network. Beginning with a predefined reference halogen atom, $N_1 = (x_1, y_1)$, the algorithm stores this atom's positional coordinates and pixel intensity within the node. From here, a probabilistic model is developed as:

$$P(N_i = H | N_1 = H) \propto exp\,(-\frac{d_I}{\sigma_I} - \frac{d_\theta}{\sigma_\theta} + \frac{d_D}{\sigma_D}).$$

which represents the probability that the next atom N_i belongs to the same halogen group as the initial reference atom N_1 is proportional to a weighted geometric factor $exp\,(-\frac{d_I}{\sigma_I} - \frac{d_\theta}{\sigma_\theta} + \frac{d_D}{\sigma_D})$. Here, d_D represents the distance between two atoms (the edge length), d_I represents the intensity difference between the two atoms, and d_θ denotes the angle between the connecting edge and the diagonals of the initial reference point and σ_D, σ_I and σ_θ are scaling factors that control the sensitivity to distance, intensity, and angle. Since there are two diagonals, each edge will yield two angle values, only the smaller one is retained, while the larger is discarded. The algorithm proceeds by recursively applying this process, repeating the calculation for each depth up to eight iterations. For each depth, eight candidate atoms are identified based on their proximity and intensity in relation to the initial atom. However, only the four atoms with the highest probabilities P are retained, while the others are discarded. From these four selected atoms, the algorithm recurses, applying the same logic to construct the entire linkage network. This iterative approach gradually builds a refined, probabilistically weighted atomic connectivity structure that is both geometrically sound and informed by the intrinsic properties of the atoms themselves.

Due to the diminishing clarity of the image quality as it approaches the particle's surface, the atomic information in these regions becomes increasingly ambiguous and imprecise. The positions of surface atoms can either be overlooked or erroneously assigned as a result of intricate, distorted complex peak signals, leading to inaccuracies in identifying their true locations. Compounding this, error signals from the underlying substrate can introduce spurious data points, causing unintended markers to appear outside the particle boundary—an unavoidable artifact during the network construction process. Consequently, the overall shape of the particle which includes the outermost atomic layer, often emerges as irregular and asymmetrical. In alignment with the algorithmic framework described earlier, the initial point N_1 is selected randomly, setting the foundation for the first segment of the halogen octahedral tilt network. This algorithm initiates the construction of a regular, grid-like network structure, systematically expanding outward until it

nears the particle's surface. At this boundary, the process encounters distortion and erroneous connections due to the presence or absence of artifact dots near or just beyond the particle's surface. The first recursion depth is then defined as α_1. To encompass the entire irregularly shaped particle, including the outermost atomic layer, a subsequent initial point, N_2, must be promptly chosen after the completion of the first section. The same process is repeated iteratively, each cycle constructing a new segment of the tilt network until the full contour of the particle, with all its irregularities and surface anomalies, is thoroughly covered. Thus the entire construction process can be described as:

$$\mathrm{C} = \sum_{i=1}^{k} \alpha_i \mathrm{N}_i + \mu ,$$

where C represents the entire connected structure, α denotes the recursion depth from each selected point as previously defined, k signifies the total number of points required to fully encompass the structure, and μ accounts for the inevitable external error connections. Armed with these parameters, we can now formalize the minimum-depth optimization challenge. The objective is to minimize both the number of selected points and their respective depths, ensuring the most efficient coverage of the structure, which can be expressed as follows:

$$min \ \|\sum_{i=1}^{k} \alpha_i + k\|_1,$$

$$\text{s.t. } \{\alpha_1, \alpha_2, \ldots \alpha_k, \sum_{i=1}^{k} \alpha_i\} \in \mathrm{C} \text{ and } k \in \mathrm{N}.$$

where N represents the set of all points within the particle. The time complexity for evaluating this structure is estimated at $O(N^3)$. It is essential to note that, during each iteration, the selection of sub-points should focus on regions surrounding the outermost layers of the previously established linkage framework. Incorporating this precondition significantly reduces the actual runtime, which proves crucial for the algorithm's efficiency and performance.

Supplementary Note 4: HAADF&iDPC Image Simulations Reveal Limited I/Br Contrast Differentiation in $CsPbX_3$ (X = I/Br)

To assess whether the substitution of Br for I at the X-site in $CsPbI_3$ can be localized through contrast variations relative to Pb in both HAADF&iDPC-STEM images, we performed image simulations on the two end-member compounds: pure $CsPbI_3$ and pure $CsPbBr_3$, and extracted the corresponding intensity line profiles to quantify the contrast difference between I and Br. However, simulation results and the extracted line profiles (**Figure. S44**) reveal that the peak intensity ratios of Br to I deviate minimally, almost the same in both two types of images. This result underscores the significance and necessity of the employed mapping methodology in visualizing potential elemental distributions and structural dynamics.

Supplementary Note 5: Validation of Atomic Column Position Fidelity in iDPC-STEM

Atomic-scale structural analysis based on phase-contrast STEM imaging requires careful consideration of imaging geometry and potential electron-optical artifacts that may affect the apparent positions of atomic columns. In this work, all atomic-resolution HAADF- and iDPC-STEM images were acquired with the quantum dots oriented close to a major crystallographic zone axis, as verified by the projected lattice symmetry and the corresponding FFT patterns, which are consistent with a [001] projection of the orthorhombic perovskite phase (**Figure. S1**). Under these imaging conditions, atomic columns are clearly resolved in both HAADF- and iDPC-STEM images, which are recorded simultaneously during the same probe scan, resulting in the mutually consistent atomic column positions from the two modalities (**Figure. S1**). The in-plane lattice parameters extracted from the iDPC-STEM images agree well with known crystallographic values, further supporting the validity of the projected atomic registry. In addition, the specimen thickness was on the order of around 15 nm, under which, the iDPC-STEM images do not exhibit signatures of severe probe-channeling-induced column elongation, contrast inversion, or other thickness-related artifacts that could compromise positional accuracy. And the absence of pronounced directional asymmetry, streaking, or systematic distortions in the experimental iDPC-STEM images further supports that the imaging conditions employed here are well within the regime suitable for sub-picometer-scale atomic position determination. Also, while small specimen tilts on the order of a few milliradians can, in principle, induce apparent atomic displacements in phase-contrast STEM imaging, such effects are mitigated here through a combination of experimental control and analysis strategy. In particular, octahedral tilting is not inferred from individual atomic displacements or single Pb-X-Pb bond angles, but instead is quantified by averaging the orientations of four Pb-X bonds within each PbX_6 octahedron, which suppresses spurious angular deviations arising from local contrast asymmetries or projection effects.

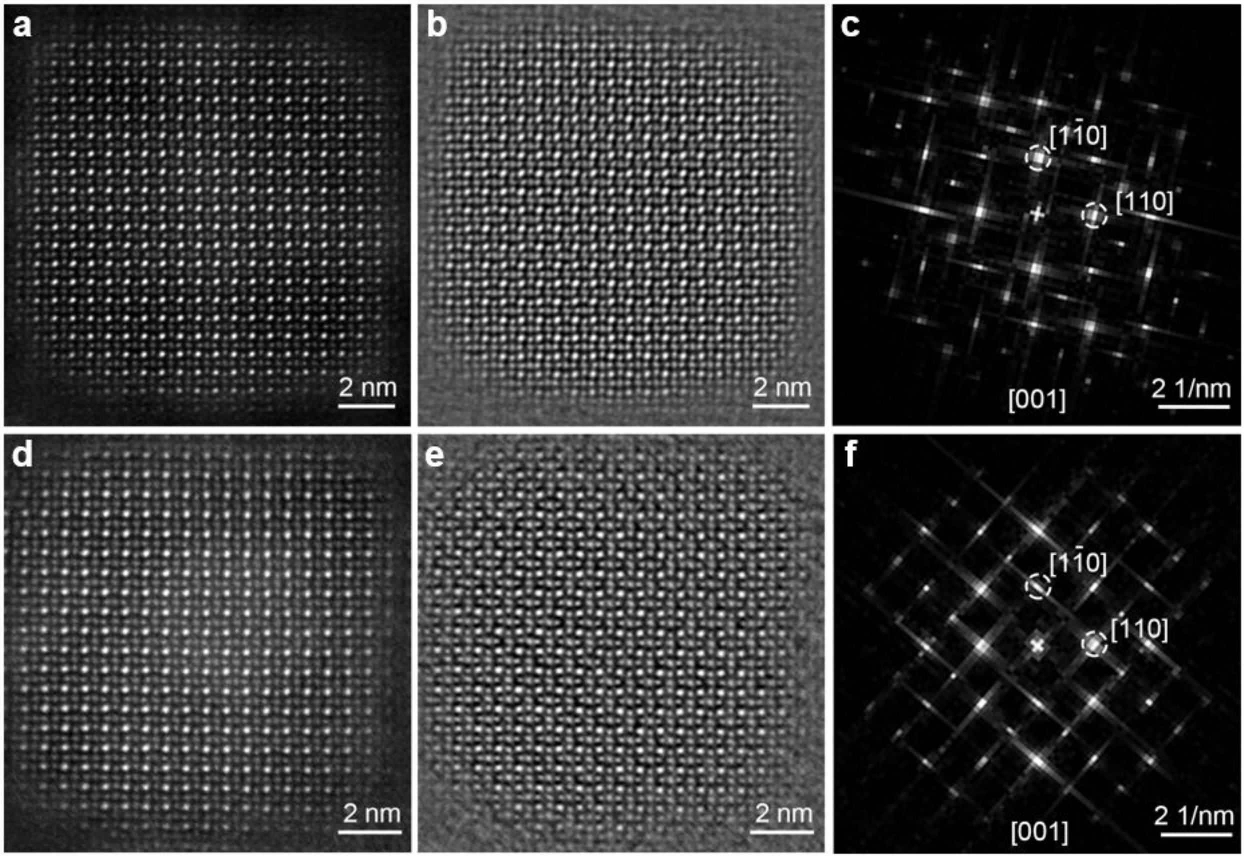

Figure S1 Structural characterization of $CsPbI_3$ and $CsPbI_{3-x}Br_x$ (x = 0.5) perovskites. HAADF-STEM images of **a,** $CsPbI_3$ and **d,** $CsPbI_{3-x}Br_x$ ($x = 0.5$). iDPC-STEM images of **b,** $CsPbI_3$ and **e,** $CsPbI_{3-x}Br_x$ ($x = 0.5$). FFT of **c,** $CsPbI_3$ and **f,** $CsPbI_{3-x}Br_x$ ($x = 0.5$). The consistent lattice symmetry and atomic column registry observed in both HAADF- and iDPC-STEM images confirm near-zone-axis alignment and support the reliability of atomic position extraction used for quantitative tilt analysis.

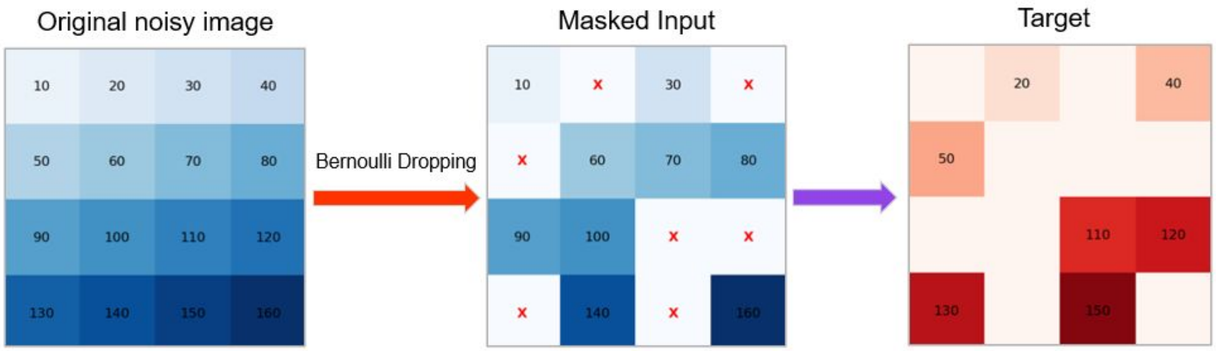

Figure S2 Bernoulli masking used for self-prediction: starting from a noisy image y. A random mask drops a subset of pixels (red "x"), yielding the masked input $\tilde{\boldsymbol{y}}$. The target is the complementary part $\bar{\boldsymbol{y}} = (\mathbf{1} - \boldsymbol{r}) \odot \boldsymbol{y}$ ($\boldsymbol{r}$ $\boldsymbol{denotes\ mask}$); the network is trained only on these hidden pixels. At inference, multiple stochastic forward passes (dropout enabled) are averaged to produce the final denoised output.

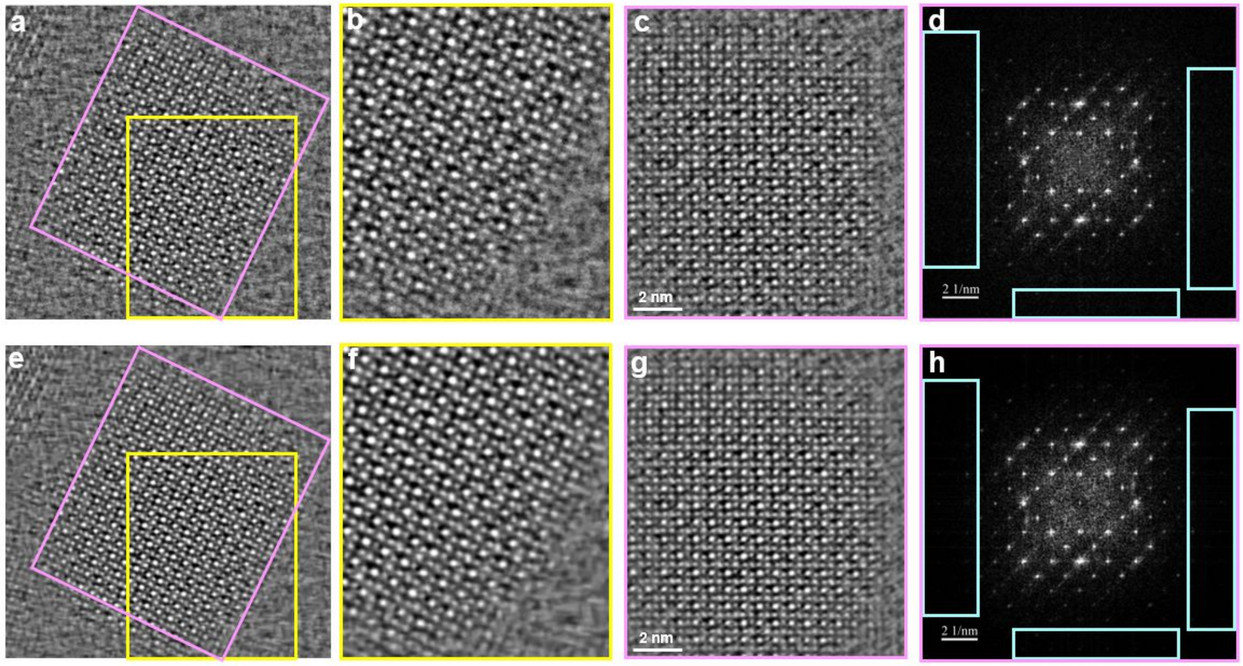

Figure S3 S2SRED denoising of iDPC-STEM images of specific $CsPbI_{3-x}Br_x$ (*x* = 0.5) QD. a-c, Representative ABSF-filtered iDPC STEM images of one specific $CsPbI_{3-x}Br_x$ (*x* = 0.5) QD sample, showing the raw contrast-limited signal. Atomic columns are discernible but obscured by significant shot noise, complicating reliable lattice interpretation. **b-c,** Magnified region (yellow and magenta box in **a**, highlights the difficulty of unambiguously resolving adjacent atomic sites due to background fluctuations. **d,** FFT of the QD region indicated by the magenta box in **a,** and **c**, displaying diffuse Bragg reflections and weak lattice-frequency components. **e-g,** Corresponding images after denoising with the S2SRED method. The S2S-restored micrographs **e,** preserve the intrinsic crystallographic periodicity while markedly suppressing high-frequency noise, thereby enhancing the visibility of both A-site cations and halide sublattices. **f-g,** Enlarged view of the boxed region in **e**, demonstrating that subtle modulations in column intensity become more faithfully resolved after S2SRED. **h,** FFT of the denoised QD region (magenta box in **d** and **g**), where lattice-frequency spots appear sharper and more intense marked by the cyan-boxes, demonstrating that S2SRED improves both real-space contrast and reciprocal-space lattice-signal recovery.

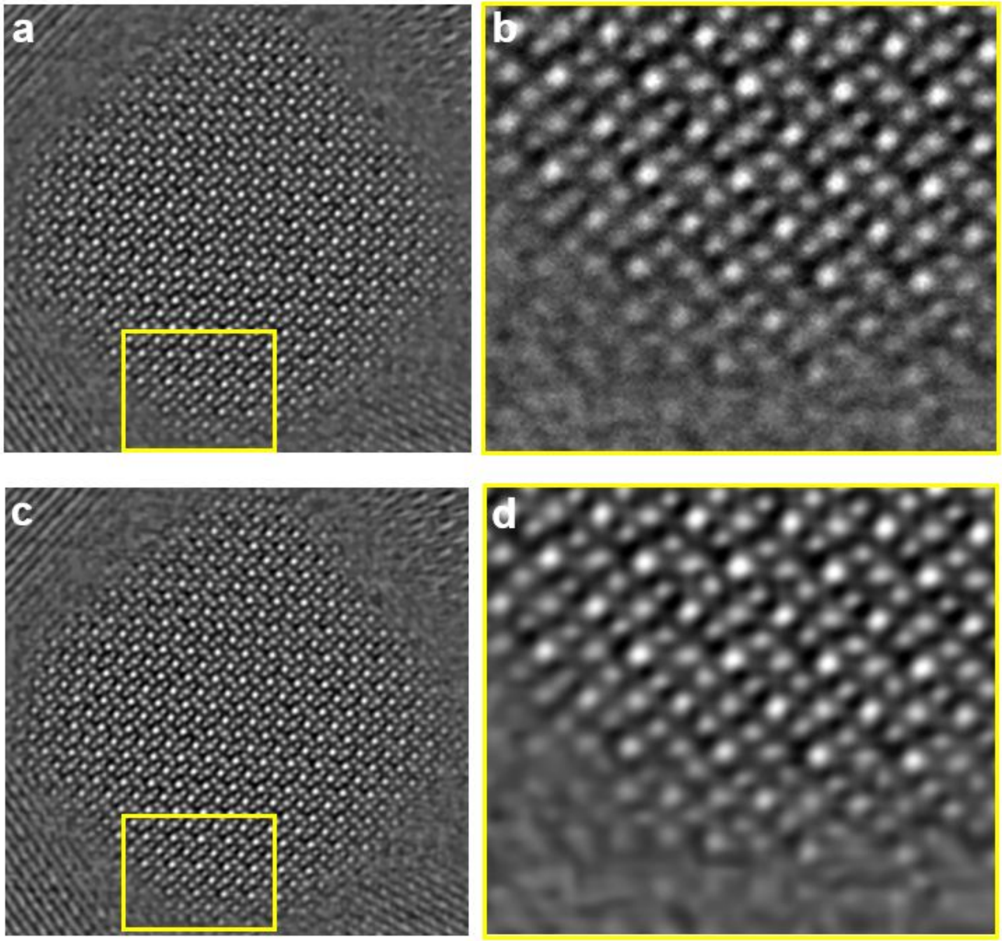

Figure S4 S2SRED denoising of iDPC-STEM images of specific $CsPbI_{3-x}Br_x$ (x = 0.5) QD. a-b, Representative ABSF-filtered iDPC STEM images of another specific $CsPbI_{3-x}Br_x$ QD sample. **b,** Magnified region of the yellow box in **a**, illustrating the degraded signal-to-noise ratio. **c-d,** Corresponding images after denoising with the S2SRED method. **d,** Enlarged view of the boxed region in **c**, the improvement is especially apparent, as the perovskite lattice emerges with enhanced clarity and uniformity, enabling reliable extraction of atomic positions for subsequent structural analysis.

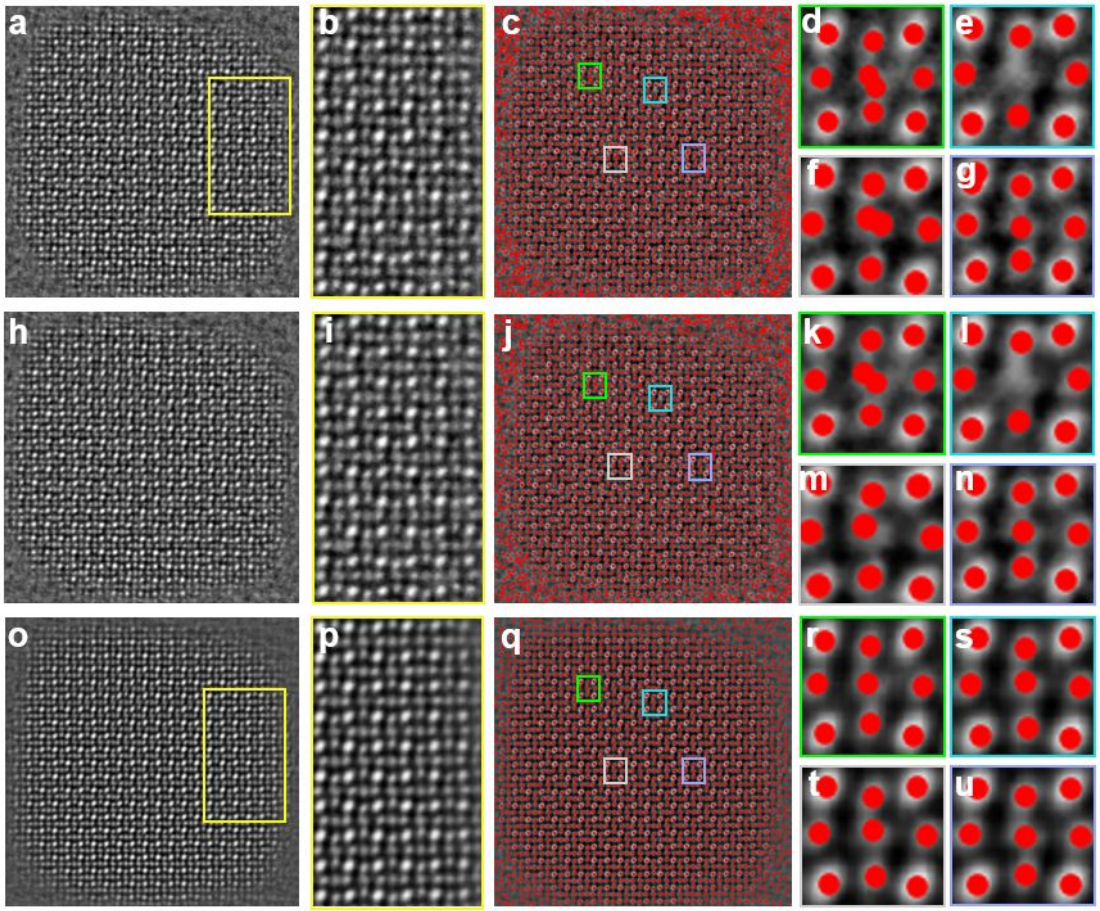

Figure S5 Comparison of atomic center detection based on 2D Gaussian fitting applied to ABSF-filtered, BM3D-denoised, and S2SRED-denoised iDPC-STEM images of a representative CsPbI3 QD. a-b, ABSF-filtered original iDPC-STEM image and the corresponding magnified region indicated by the yellow box. **c,** Atomic column centers identified by 2D Gaussian fitting overlaid on the original image, with color-coded boxes marking representative local regions. **d-g,** Enlarged views of the boxed regions in **c,** showing fitted Gaussian peaks (red) superimposed on the original atomic contrast, where peak localization is visibly affected by residual noise and contrast non-uniformity. **h-I,** BM3D-denoised iDPC-STEM image and the corresponding magnified inset. **j,** Atomic centers obtained by applying the same 2D Gaussian fitting procedure to the BM3D-denoised image. **k-n,** Enlarged local views from **j,** illustrating that BM3D denoising improves overall signal smoothness and peak separability compared to the original image, yet residual structural blurring and local contrast attenuation persist, leading to occasional peak broadening and reduced fitting robustness in closely spaced atomic columns. **o-p,** S2SRED-denoised iDPC-STEM image and the corresponding magnified region. **q,** Atomic centers extracted via 2D Gaussian fitting overlaid on the S2SRED-denoised image. **r-u,** Enlarged local views from **q,** demonstrating sharply defined atomic contrast and consistently well-localized Gaussian peaks, with minimal peak distortion or spurious merging. Compared with both the ABSF-filtered and BM3D-denoised images, S2SRED provides superior preservation of atomic column shape and intensity symmetry, enabling the most reliable and spatially consistent atomic center detection.

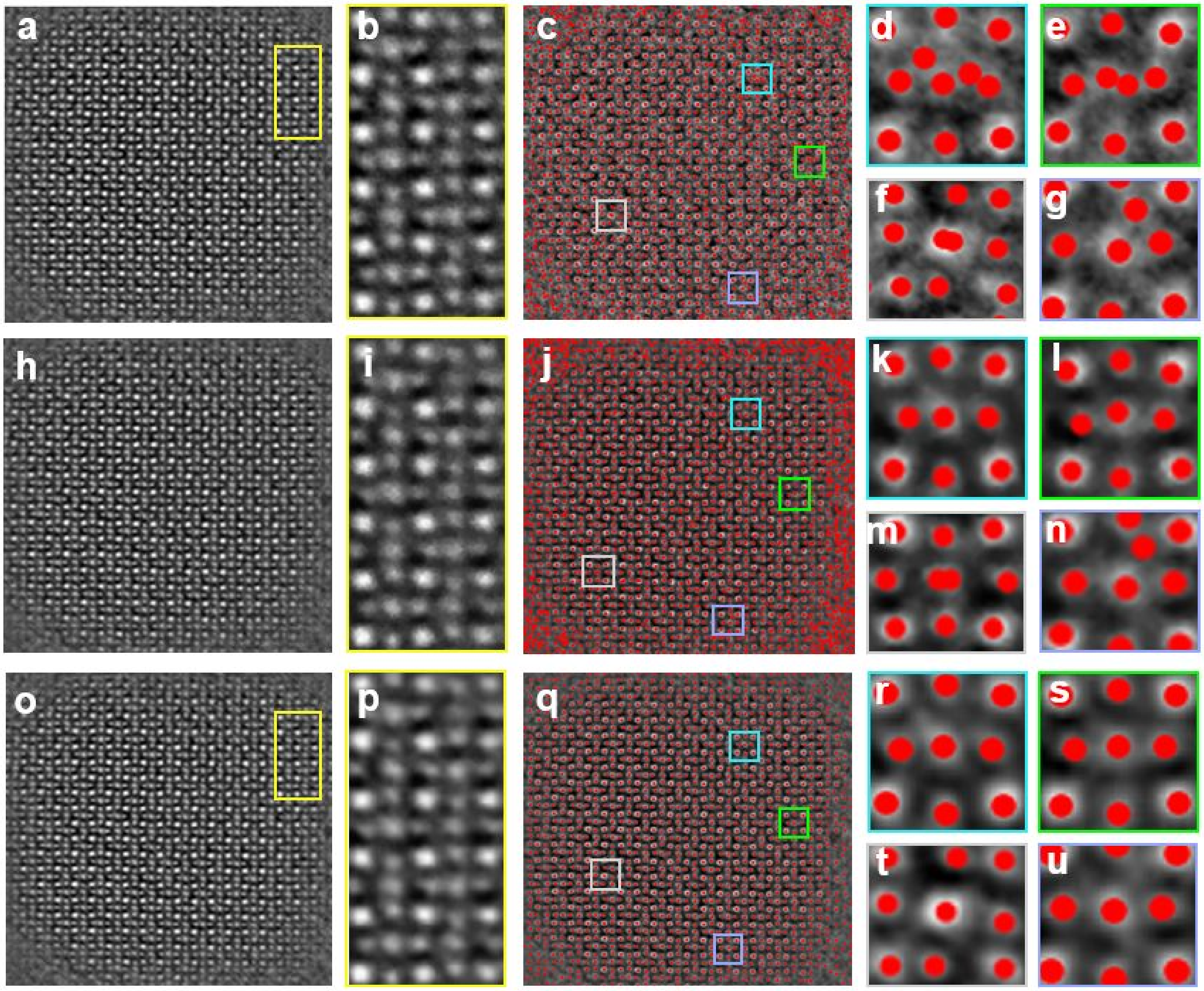

Figure S6 Comparison of atomic center detection from ABSF-filtered, BM3D-denoised, and S2SRED-denoised iDPC-STEM images of a $CsPbI_{3-x}Br_x$ (x = 0.5) QD. a-b, Original ABSF-filtered iDPC-STEM image of a $CsPbI_{3-x}Br_x$ (x = 0.5) QD and magnified region highlighted by the yellow box. **c,** Atomic center distribution obtained by 2D Gaussian fitting on the original image, with representative regions marked for comparison. **d-g,** Enlarged views of the selected regions, showing fitted Gaussian maxima (red) overlaid on the original contrast, where compositional disorder and noise lead to increased variability in peak shapes and positions. **h-i,** BM3D-denoised iDPC-STEM image and corresponding magnified region. **j,** Atomic centers extracted from the BM3D-processed image using the same fitting protocol. **k-n,** Enlarged local regions from **j**, indicating improved contrast relative to the original image, while residual artifacts and peak asymmetry persist in locally distorted areas. **o-p,** S2SRED-denoised iDPC-STEM image and corresponding magnified inset. **q,** Atomic centers determined from the S2SRED-denoised image via identical Gaussian fitting. **r-u,** Enlarged local regions from **q**, showing uniform peak shapes and stable atomic center localization across different local environments, highlighting the superior performance of S2SRED for precise atomic center extraction in mixed-halide systems.

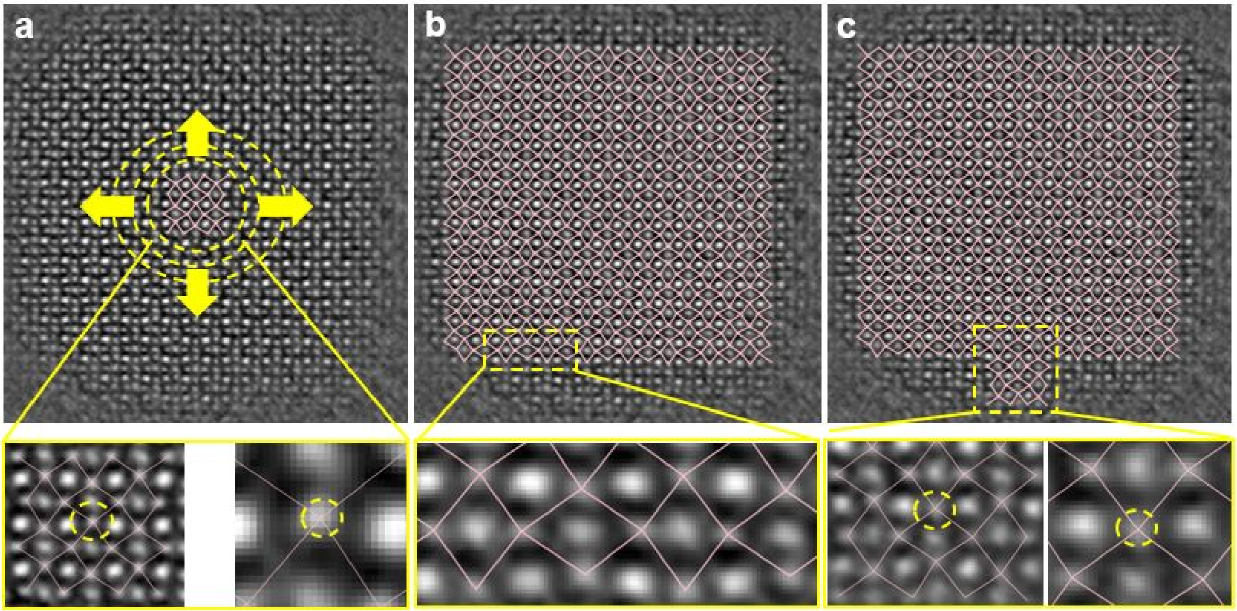

Figure S7 The stepwise procedure of the SOFS algorithm applied to a single image. a, The initial atom is identified within a magnified region outlined by the yellow framework. This marks the starting point for the first spiral depth propagation, which continues until encountering some of the error points in the background, signifying the completion of the first spiral depth. **b,** The propagation process concludes upon reaching the error dot, finalizing the spiral region. **c,** Subsequent initial atoms are automatically detected, and the diffusion process is iteratively repeated, as demonstrated in **a**, until the peripheral regions are completely traversed. This progression ensures that the outermost layer is identified, characterized by the absence of error points at maximum depth.

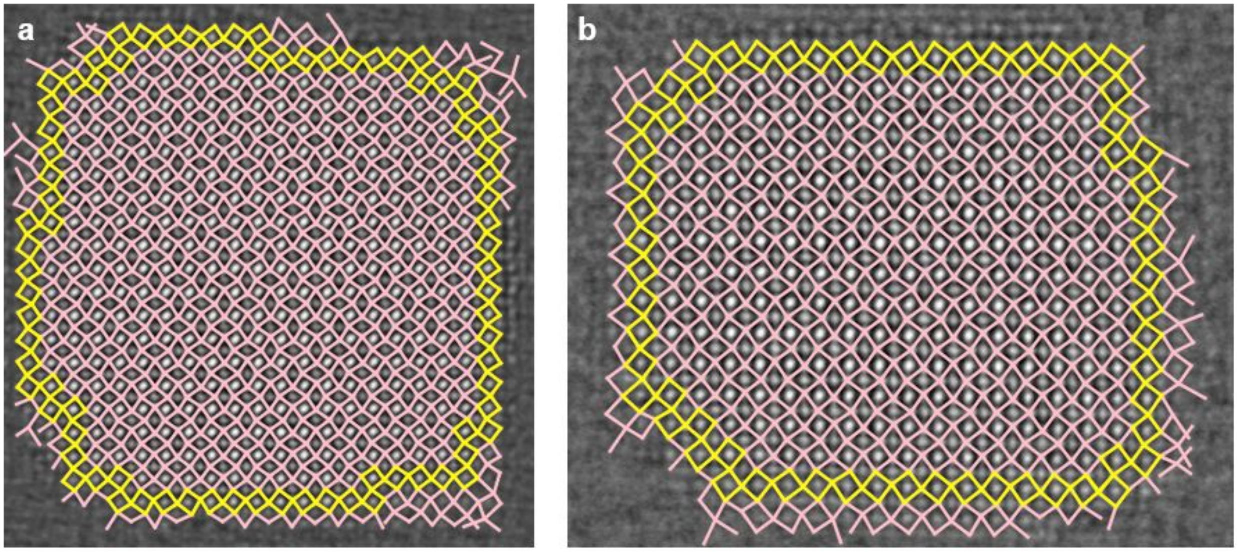

Figure S8 Representative final processed images obtained using the SOFS algorithm. a-b, Results for pure $CsPbI_3$ QD samples of varying sizes. The outermost layers, free from error points, are delineated with yellow frames. Dendritic-like connections outside the yellow frames, shown in pink, are automatically excluded from subsequent calculations.

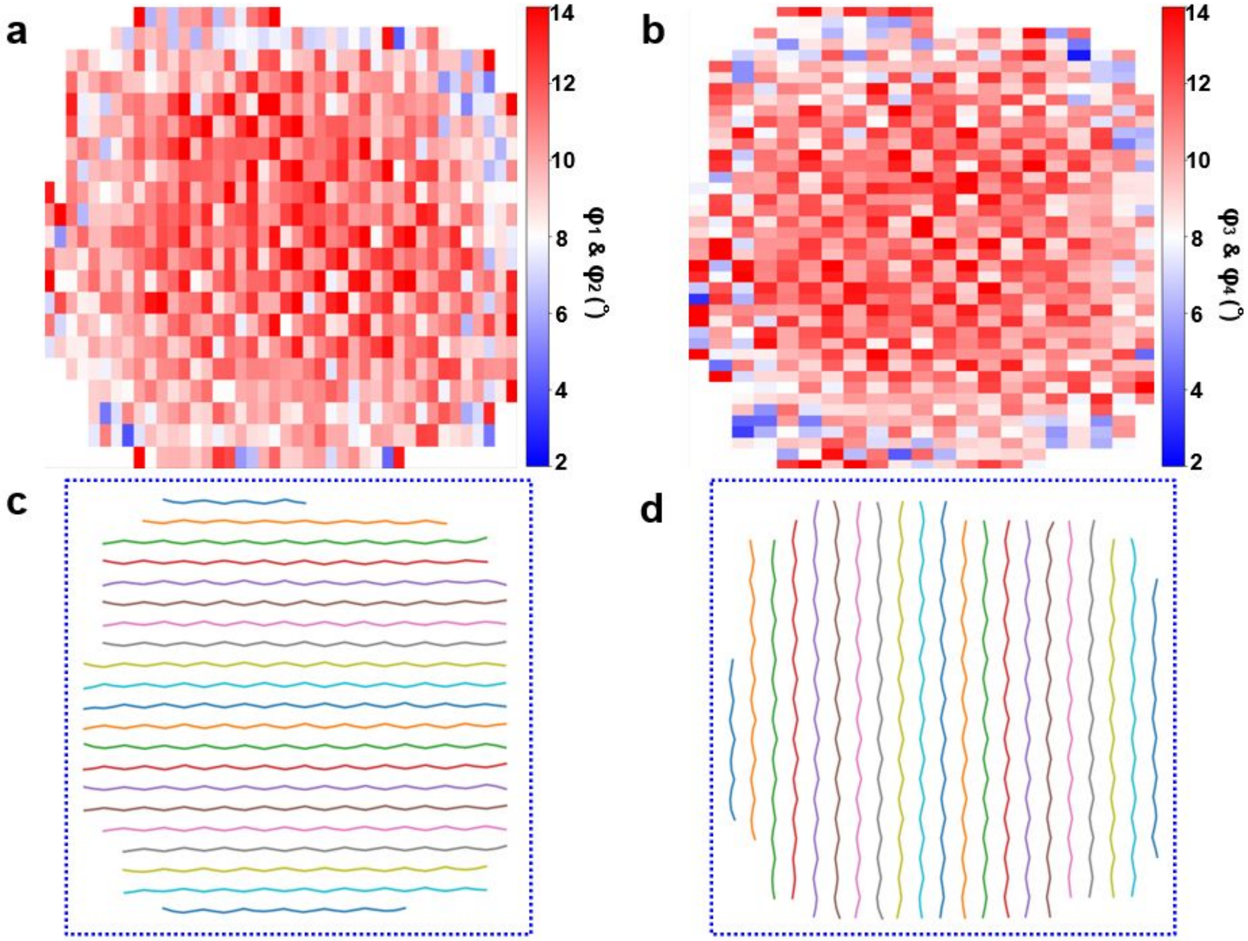

Figure S9 Tilt mappings of Pb-I in the first $CsPbI_3$ QD sample and the extracted corresponding I-Pb-I sublattice connections. a, Tilt angle mappings φ_1 and φ_2, calculated along the horizontal direction. **b,** Tilt angle mappings φ_3 and φ_4, calculated along the vertical direction. Type or paste caption here. Create a page break and paste in the Table above the caption. **c-d,** I-Pb-I lattice framework in the horizontal and vertical direction respectively.

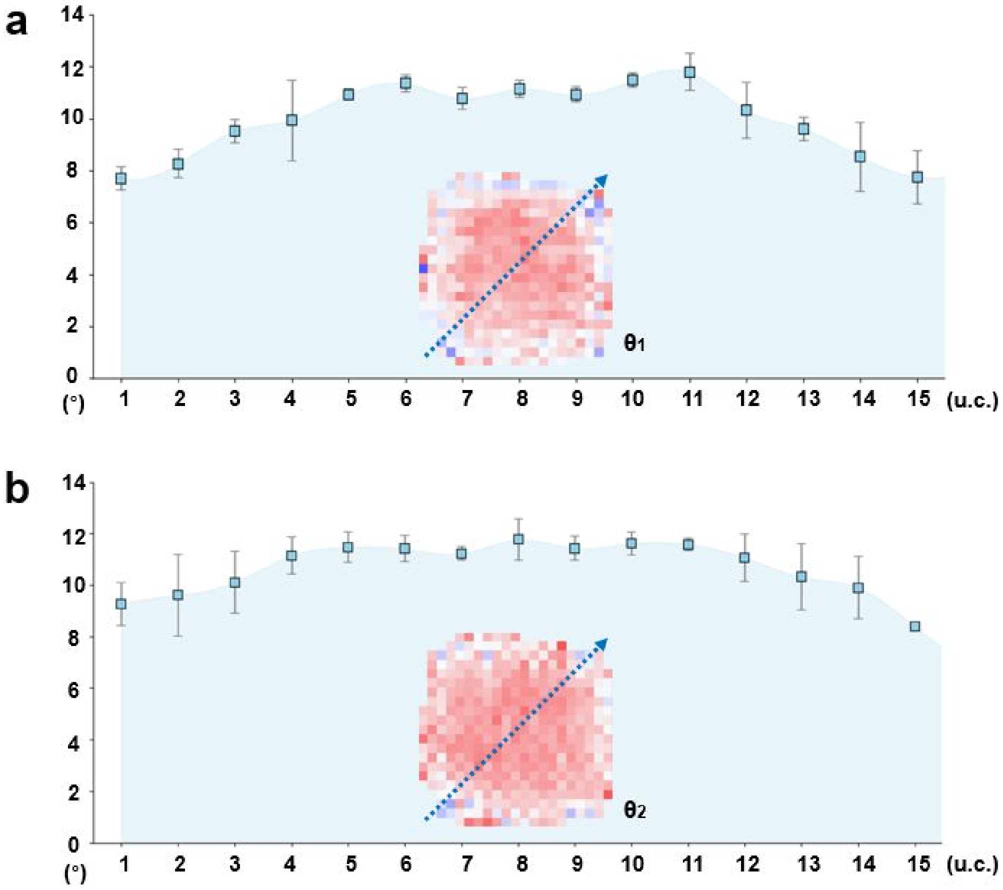

Figure S10 Line profiles of tilt angles extracted along orthogonal crystallographic directions a, θ_1 and b, θ_2 from selected orientation and regions in the first $CsPbI_3$ QD sample. The dark-blue arrow indicates the selected orientation, along which the line profile is extracted by integrating four regions adjacent to the arrow, each offset by one lattice spacing.

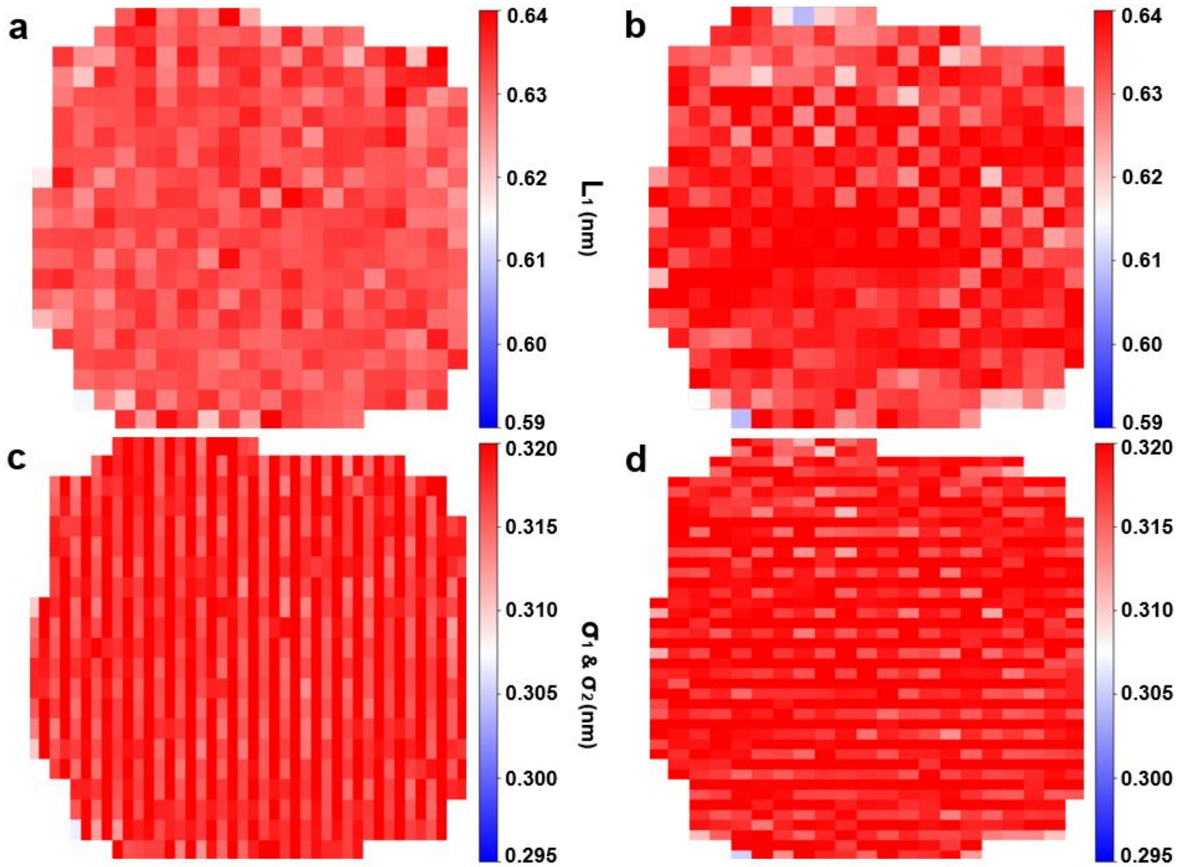

Figure S11 Bond length mappings of Pb-I in the first $CsPbI_3$ QD sample. a, Bond length mapping of L_1, obtained as the sum of σ_1 and σ_2 in every unit cell in **c**. **b,** Bond length mapping of L_2, derived as the sum of σ_3 and σ_4 in every unit cell in **d**. **c,** Bond length mapping of σ_1 and σ_2, derived from the horizontal direction. **d,** Bond length mapping of σ_3 and σ_4, derived from the vertical direction.

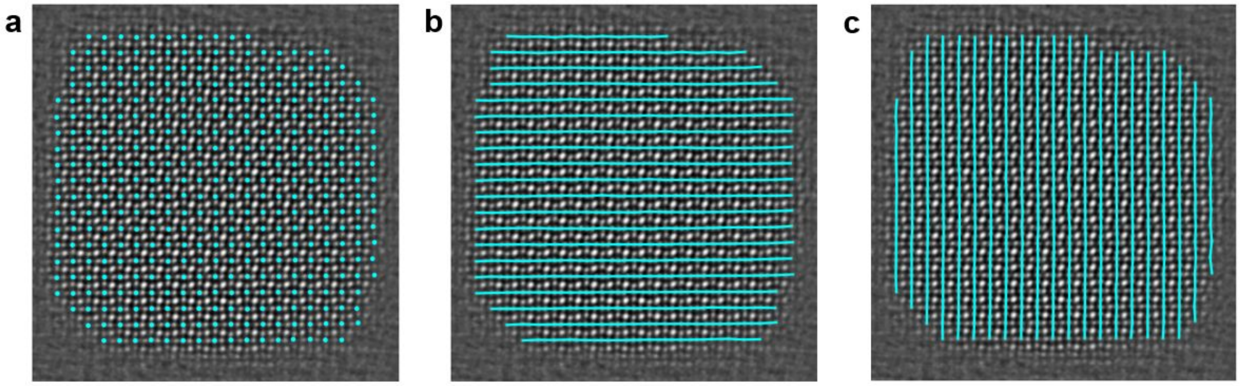

Figure S12 Characterization of Cs atoms and derived Cs-Cs sublattice in the first $CsPbI_3$ QD sample. a, Classified Cs sites, highlighted in cyan. **b,** Cs-Cs lattice periodicity resolved along the horizontal direction. **c,** Cs-Cs lattice periodicity resolved along the vertical direction.

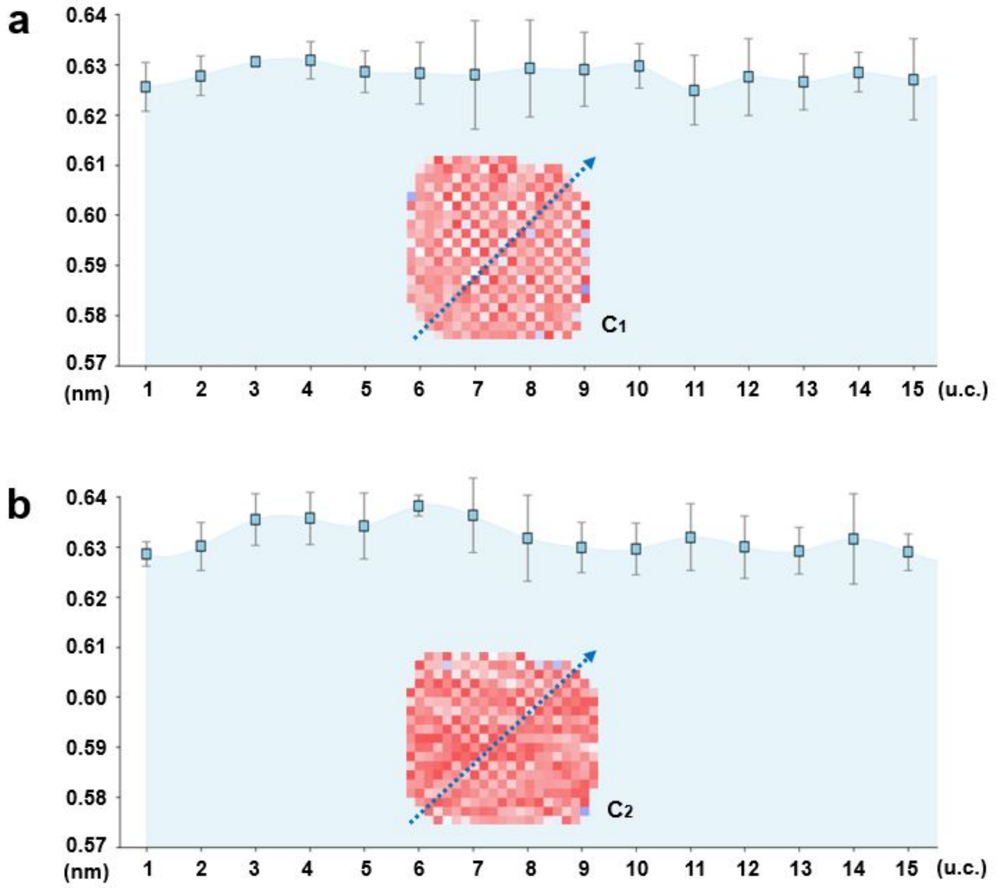

Figure S13 Line profiles of Cs-Cs bond lengths extracted along orthogonal crystallographic directions a, C_1 and b, C_2 from selected orientation and regions in the first $CsPbI_3$ QD sample. The dark-blue arrow indicates the selected orientation, along which the line profile is extracted by integrating four regions adjacent to the arrow, each offset by one lattice spacing.

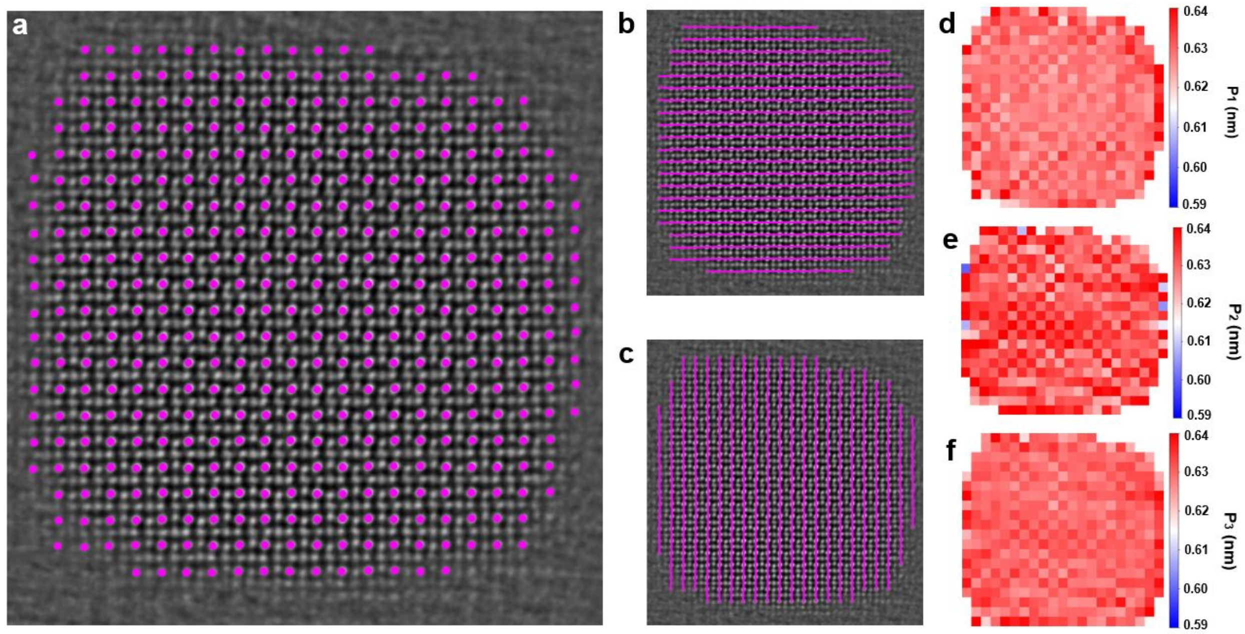

Figure S14 Characterization of Pb atoms and derived Pb-Pb sublattice and mappings in the first $CsPbI_3$ QD sample. a, Classified Pb sites, highlighted in bright purple. **b,** Pb-Pb lattice periodicity resolved along the horizontal direction. **c,** Pb-Pb lattice periodicity resolved along the vertical direction. The Pb-Pb lattice spacing mapping P_1, P_2 along the **d,** horizontal and **e,** vertical directions, respectively, and their averaged mapping **f,** P_3.

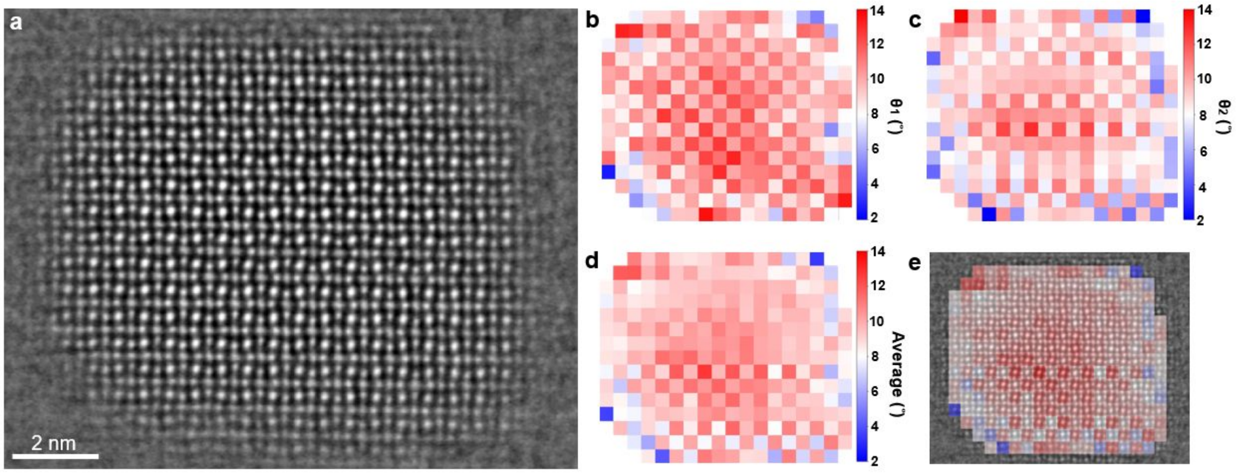

Figure S15 Characterization and tilt mappings of second $CsPbI_3$ QD sample. a, S2SRED denoised iDPC-STEM image of this sample. **b,** Mapping of the tilt angle θ_1, derived as the mean of φ_1 and φ_2 in every unit cell in (**Figure. S16a**). **c,** Mapping of the tilt angle θ_2, derived as the mean of φ_3 and φ_4 in every unit cell in (**Figure. S16b**). **d,** Composite mapping of the average tilt angles from **b** and **c**, providing an integrated perspective on structural tilting. **e,** Integration obtained by overlaying **d** onto **a** for a direct visual impression.

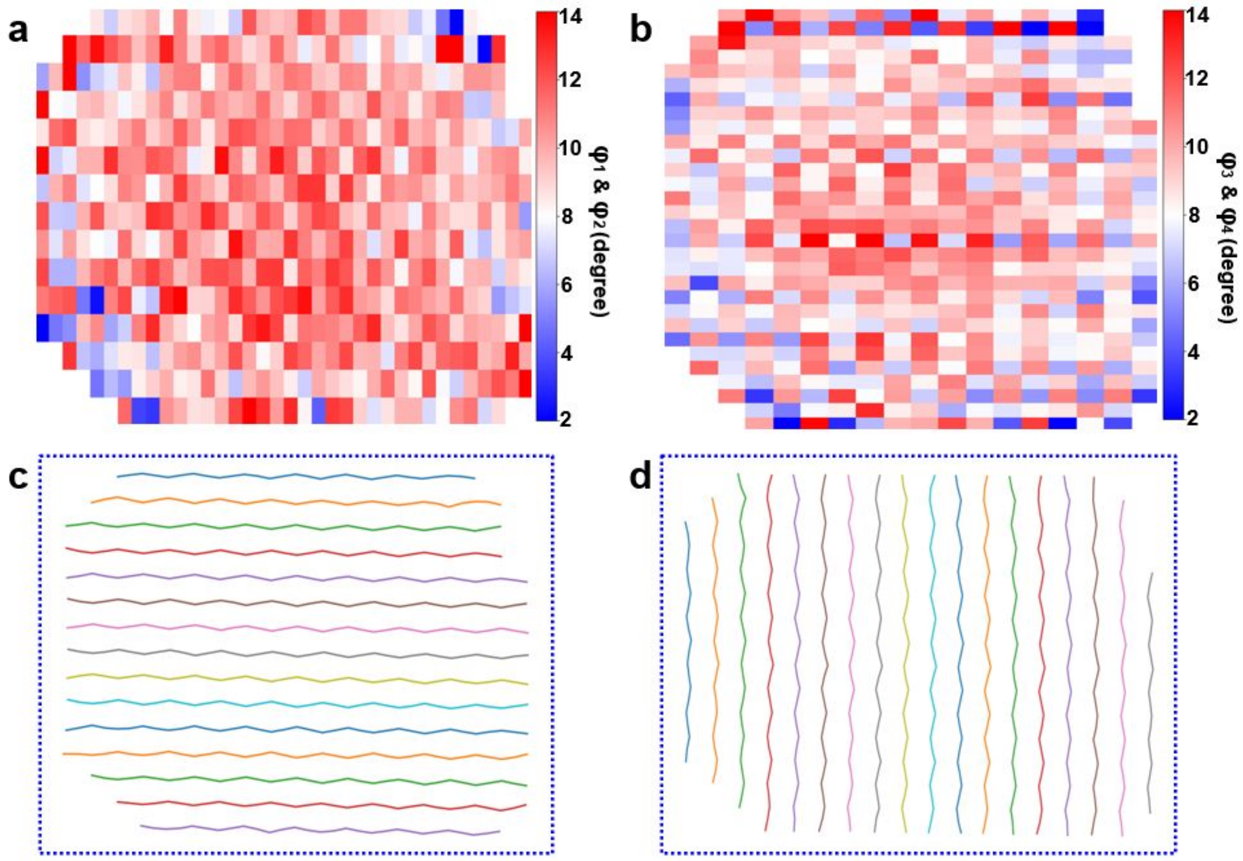

Figure S16 Tilt mappings of Pb-I in the second $CsPbI_3$ QD sample and the extracted corresponding I-Pb-I sublattice connections. a, Tilt angle mappings φ_1 and φ_2, calculated along the horizontal direction. **b,** Tilt angle mappings φ_3 and φ_4, calculated along the vertical direction. Type or paste caption here. Create a page break and paste in the Table above the caption. **c-d,** I-Pb-I lattice framework in the horizontal and vertical direction respectively.

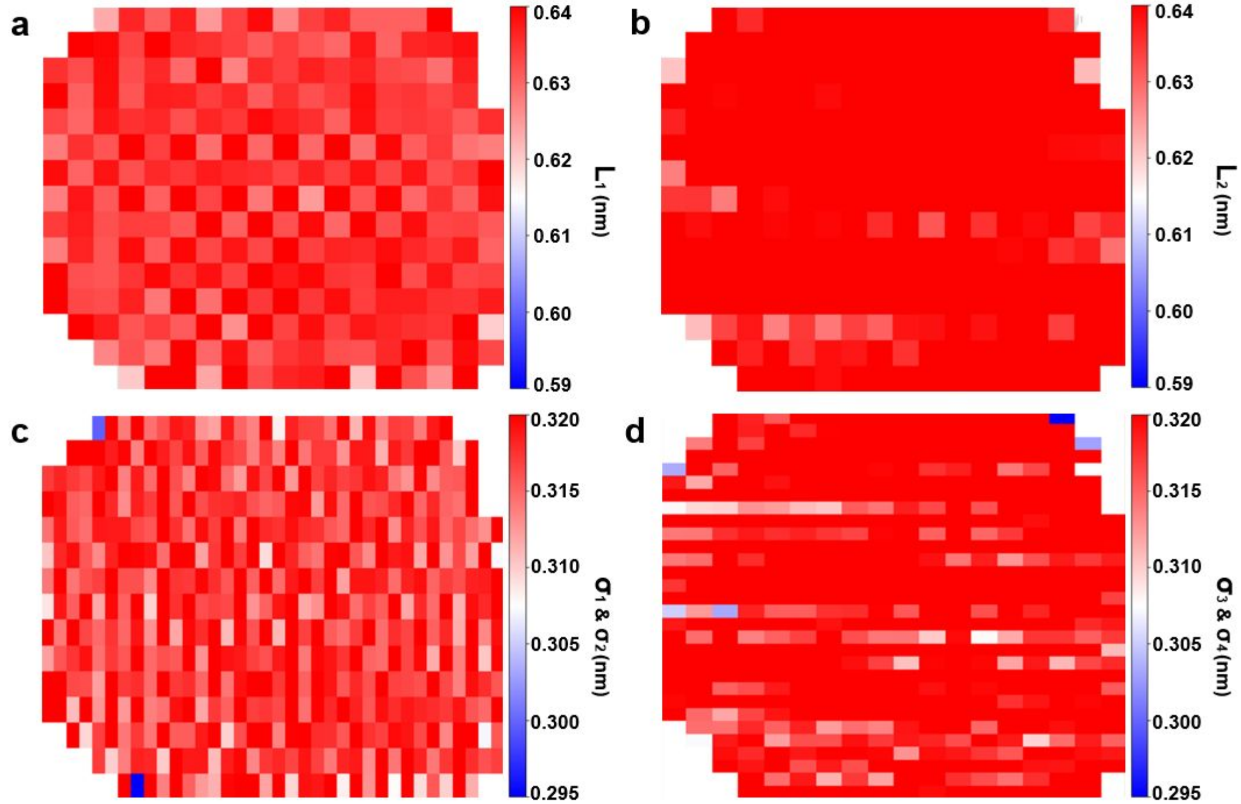

Figure S17 Bond length mappings of Pb-I in the second $CsPbI_3$ QD sample. a, Bond length mapping of L_1, obtained as the sum of σ_1 and σ_2 in every unit cell in **c**. **b,** Bond length mapping of L_2, derived as the sum of σ_3 and σ_4 in every unit cell in **d**. **c,** Bond length mapping of σ_1 and σ_2, derived from the horizontal direction. **d,** Bond length mapping of σ_3 and σ_4, derived from the vertical direction.

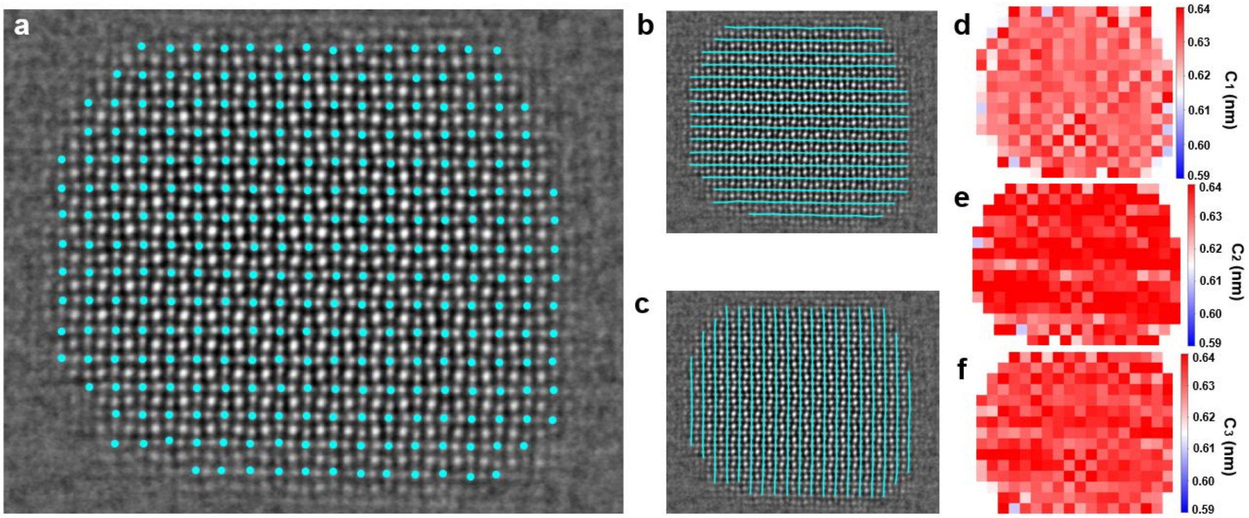

Figure S18 Characterization of Cs atoms and derived Cs-Cs sublattice and mappings in the second $CsPbI_3$ QD sample. a, Classified Cs sites, highlighted in cyan. **b,** Cs-Cs lattice periodicity resolved along the horizontal direction. **c,** Cs-Cs lattice periodicity resolved along the vertical direction. The Cs-Cs lattice spacing mapping C_1, C_2 along the **d,** horizontal and **e,** vertical directions, respectively, and their averaged mapping **f,** C_3.

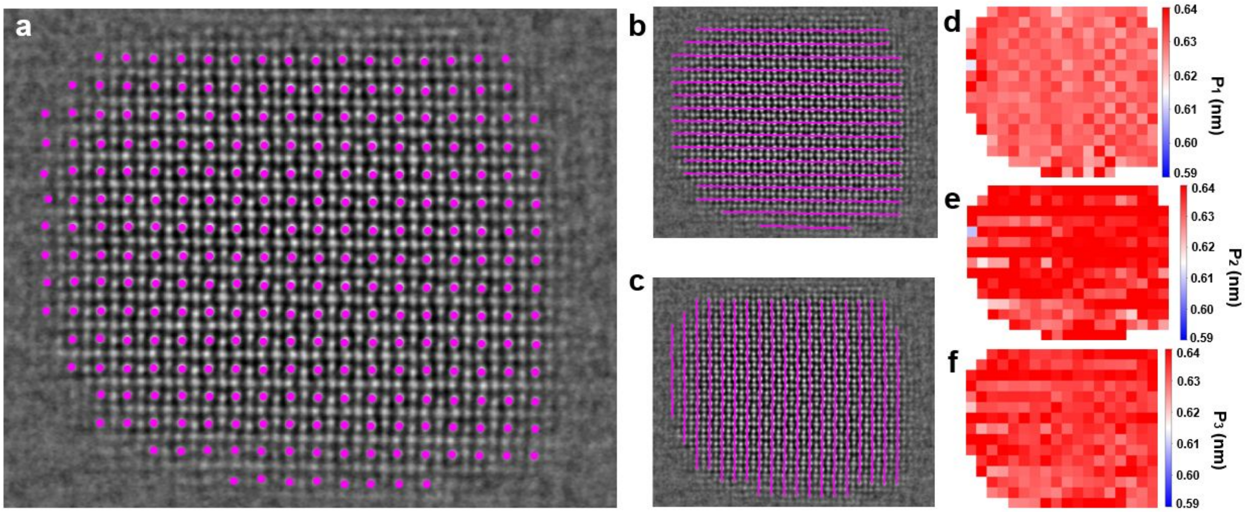

Figure S19 Characterization of Pb atoms and derived Pb-Pb sublattice and mappings in the second $CsPbI_3$ QD sample. a, Classified Pb sites, highlighted in bright purple. **b,** Pb-Pb lattice periodicity resolved along the horizontal direction. **c,** Pb-Pb lattice periodicity resolved along the vertical direction. The Pb-Pb lattice spacing mapping P_1, P_2 along the **d,** horizontal and **e,** vertical directions, respectively, and their averaged mapping **f,** P_3.

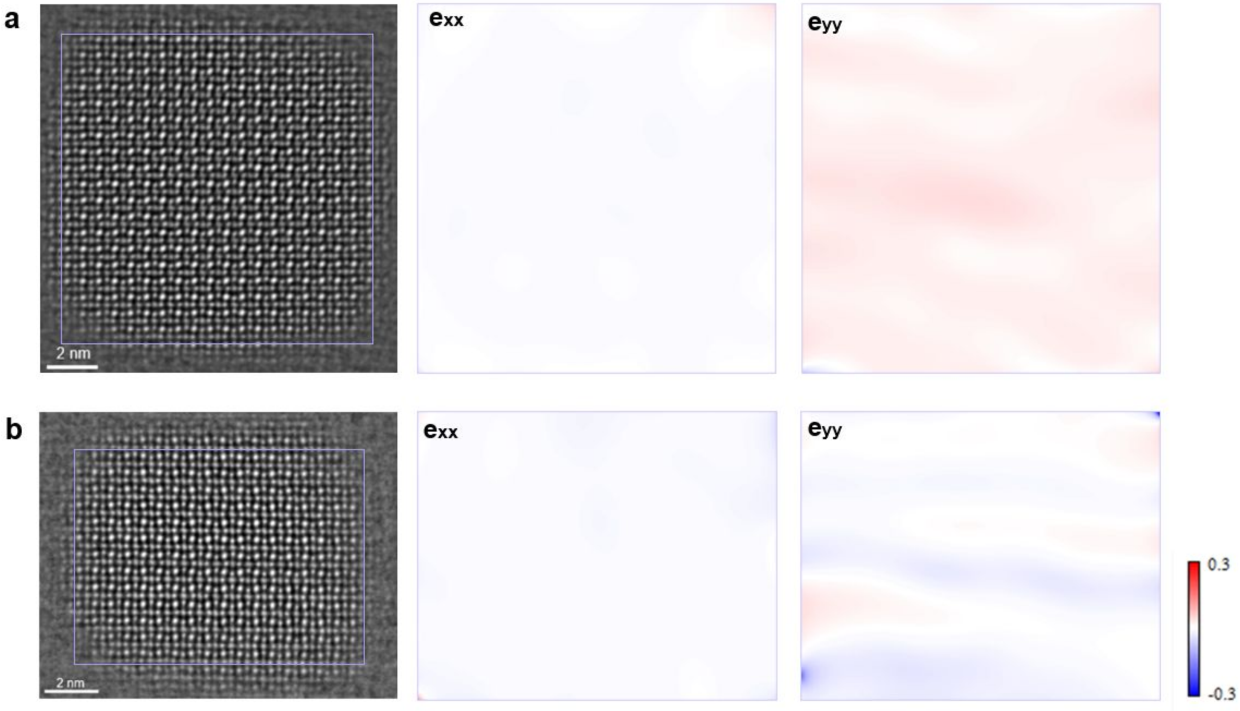

Figure S20 Geometric Phase Analysis (GPA) for the two $CsPbI_3$ samples. Both e_{xx} and $e_{\gamma\gamma}$ maps show low and spatially smooth strain within the interior regions of the quantum dots.

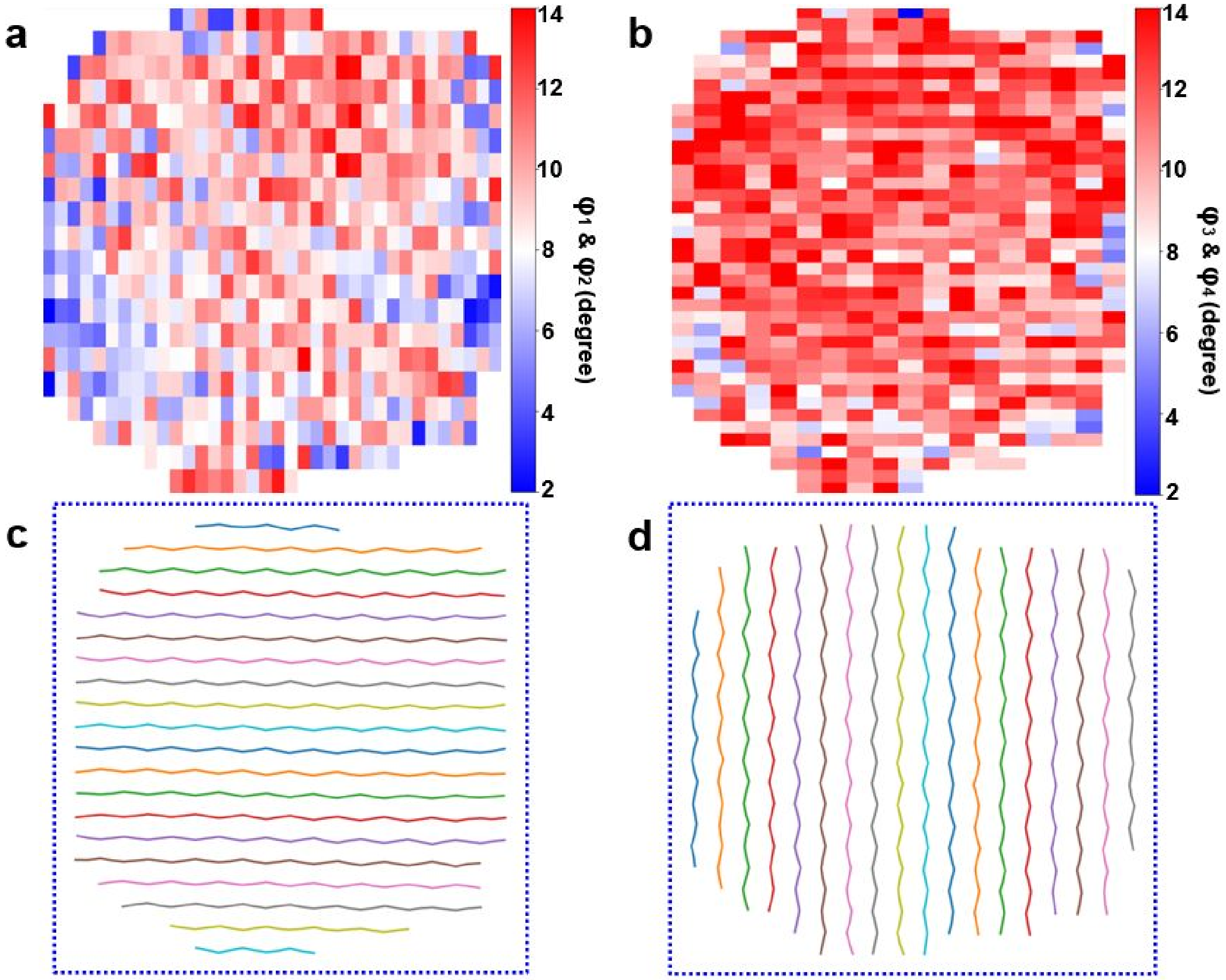

Figure S21 Tilt mappings of Pb-I in the first $CsPbI_{3-x}Br_x$ (x = 0.5) QD sample and the extracted corresponding I-Pb-I sublattice connections. a, Tilt angle mappings φ_1 and φ_2, calculated along the horizontal direction. **b,** Tilt angle mappings φ_3 and φ_4, calculated along the vertical direction. Type or paste caption here. Create a page break and paste in the Table above the caption. **c-d,** I-Pb-I lattice framework in the horizontal and vertical direction respectively.

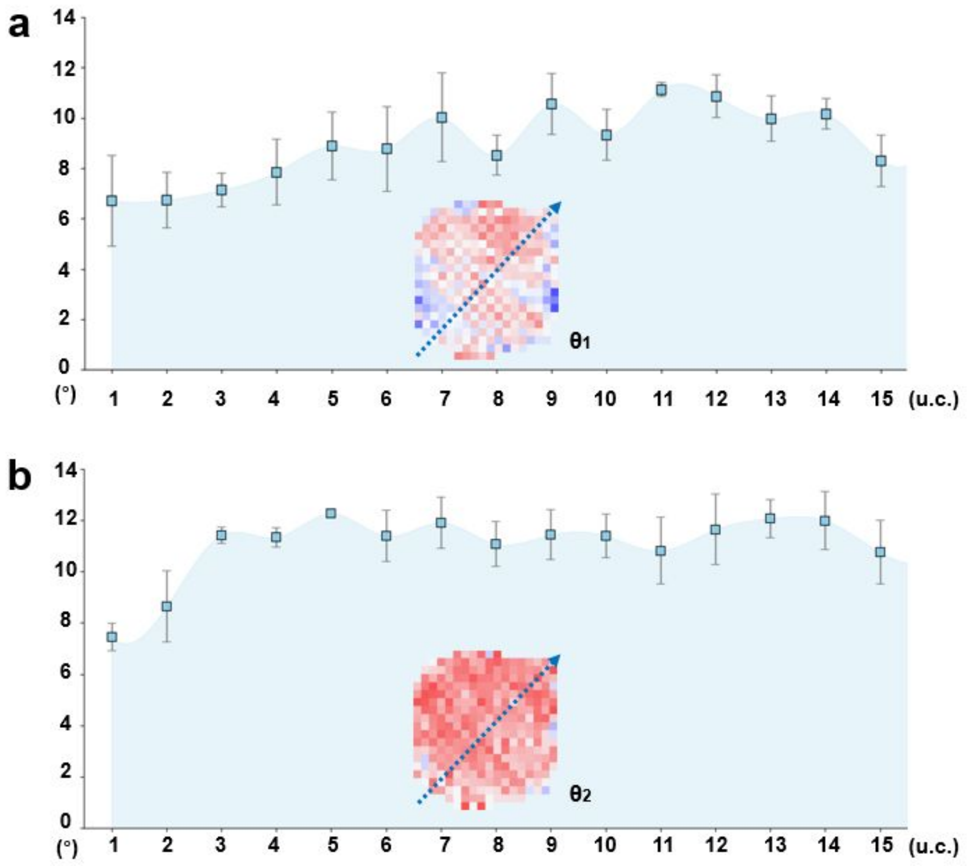

Figure S22 Line profiles of tilt angles extracted along orthogonal crystallographic directions a, θ_1 and b, θ_2 from selected orientation and regions in the first $CsPbI_{3-x}Br_x$ (x = 0.5) QD sample. The dark-blue arrow indicates the selected orientation, along which the line profile is extracted by integrating four regions adjacent to the arrow, each offset by one lattice spacing.

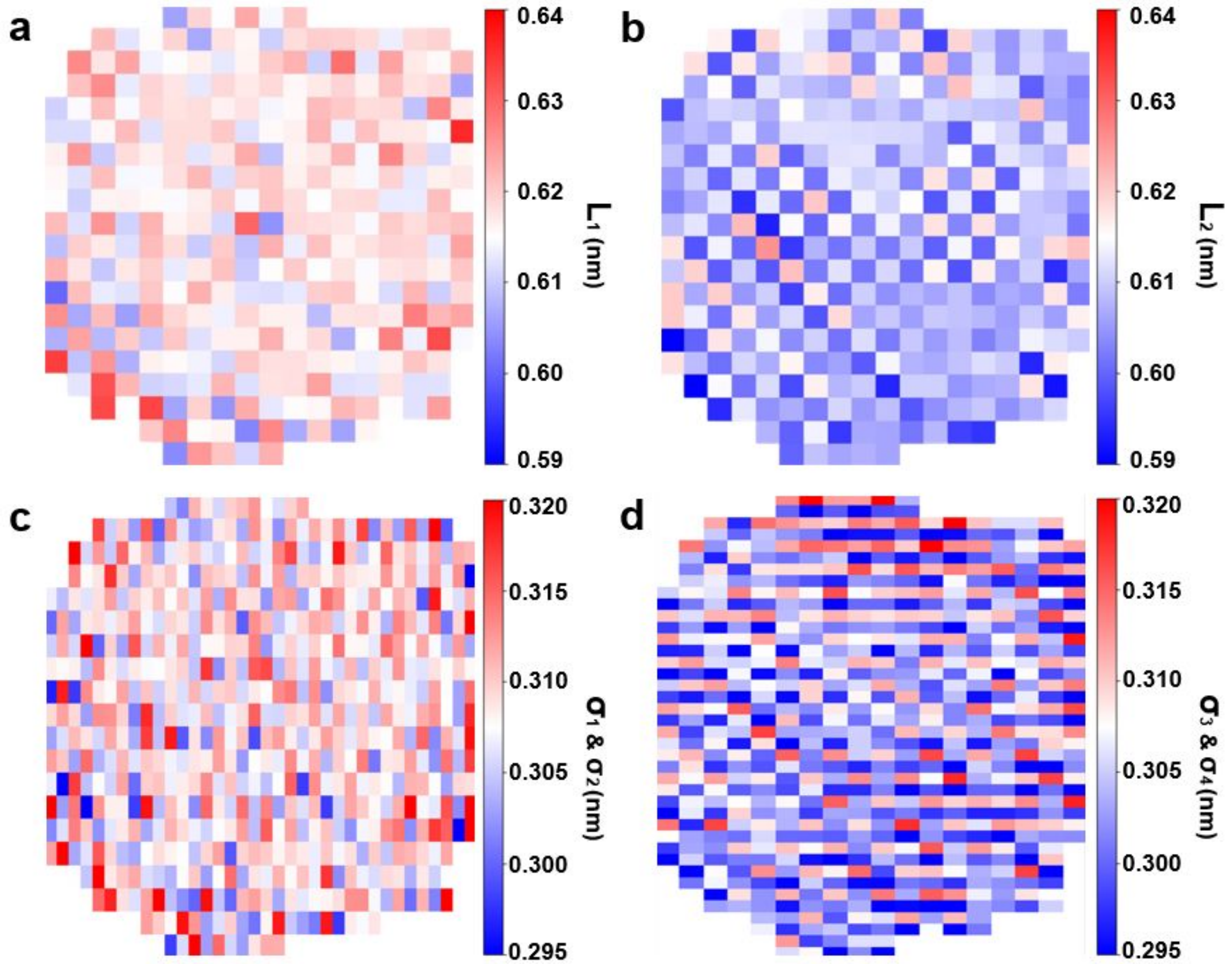

Figure S23 Bond length mappings of Pb-I in the first $CsPbI_{3-x}Br_x$ (x = 0.5) QD sample. a, Bond length mapping of L_1, obtained as the sum of σ_1 and σ_2 in every unit cell in **c**. **b,** Bond length mapping of L_2, derived as the sum of σ_3 and σ_4 in every unit cell in **d**. **c,** Bond length mapping of σ_1 and σ_2, derived from the horizontal direction. **d,** Bond length mapping of σ_3 and σ_4, derived from the vertical direction.

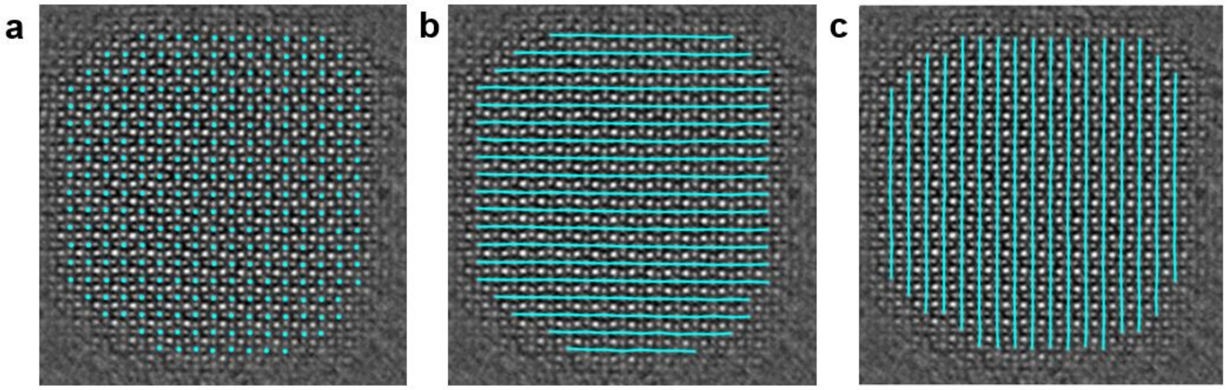

Figure S24 Characterization of Cs atoms and derived Cs-Cs sublattice in the first $CsPbI_{3-x}Br_x$ (x = 0.5) QD sample. a, Classified Cs sites, highlighted in cyan. **b,** Cs-Cs lattice periodicity resolved along the horizontal direction. **c,** Cs-Cs lattice periodicity resolved along the vertical direction.

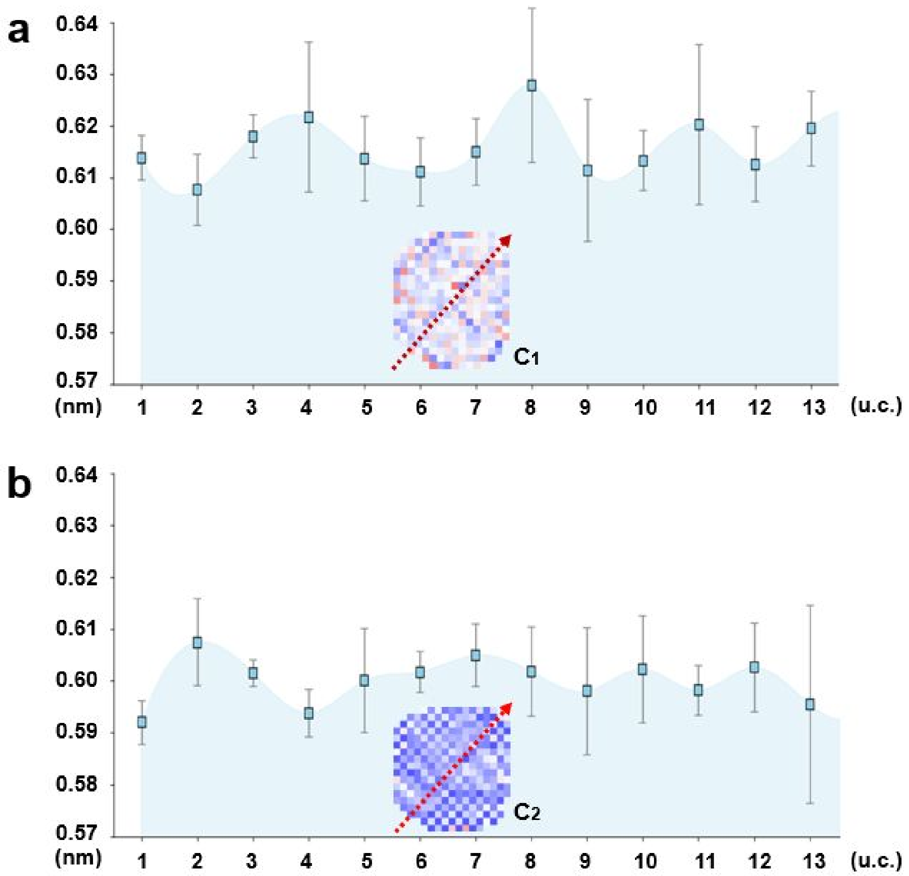

Figure S25 Line profiles of Cs-Cs bond lengths extracted along orthogonal crystallographic directions a, C_1 and b, C_2 from selected orientation and regions in the first $CsPbI_{3-x}Br_x$ (x = 0.5) QD sample. The dark-red arrow indicates the selected orientation, along which the line profile is extracted by integrating four regions adjacent to the arrow, each offset by one lattice spacing.

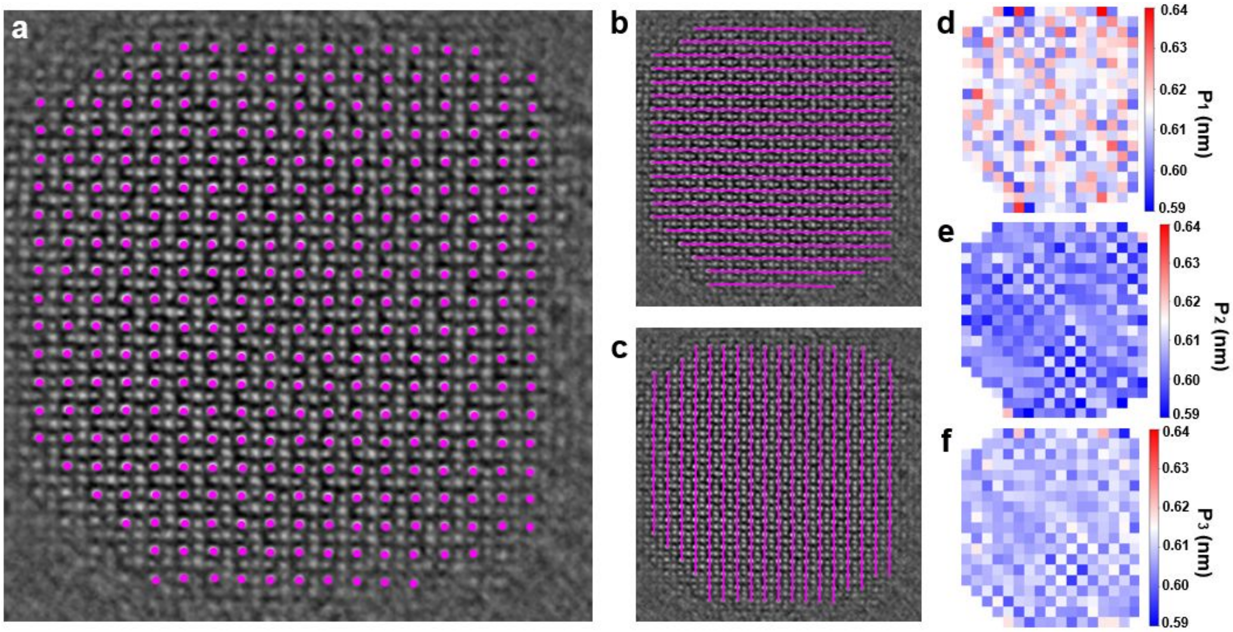

Figure S26 Characterization of Pb atoms and derived Pb-Pb sublattice and mappings in the first $CsPbI_{3\text{-}x}Br_x$ (x = 0.5) QD sample. a, Classified Pb sites, highlighted in bright purple. **b,** Pb-Pb lattice periodicity resolved along the horizontal direction. **c,** Pb-Pb lattice periodicity resolved along the vertical direction. The Pb-Pb lattice spacing mapping P_1, P_2 along the **d,** horizontal and **e,** vertical directions, respectively, and their averaged mapping **f,** P_3.

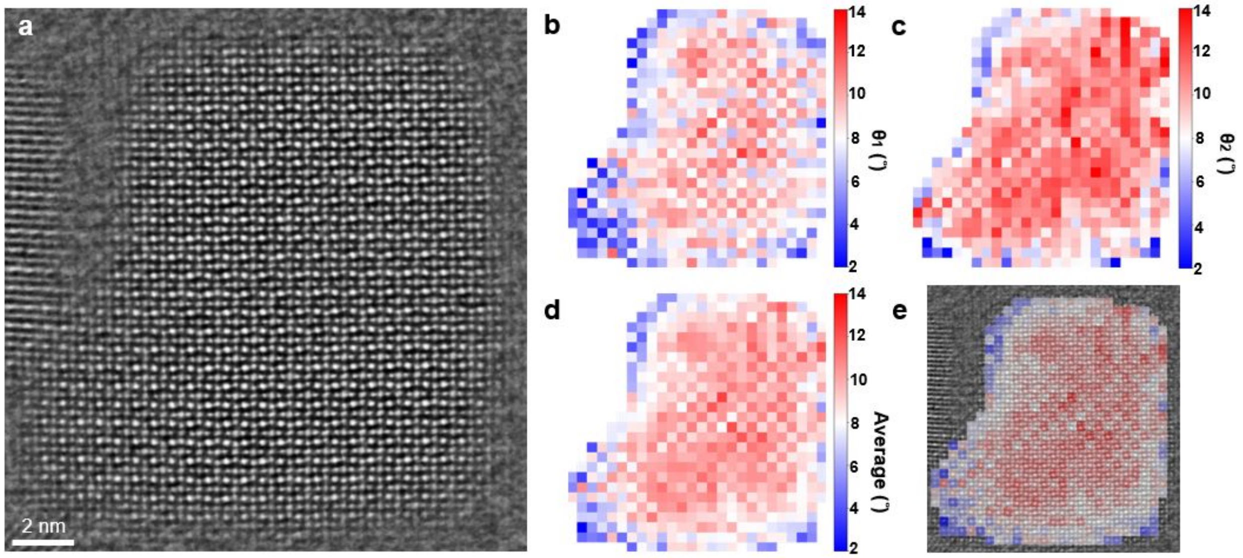

Figure S27 Characterization and tilt mappings of second $CsPbI_{3-x}Br_x$ (x = 0.5) QD sample. a, S2SRED denoised iDPC-STEM image of this sample. **b,** Mapping of the tilt angle θ_1, derived as the mean of φ_1 and φ_2 in every unit cell in (**Figure. S27a**). **c,** Mapping of the tilt angle θ_2, derived as the mean of φ_3 and φ_4 in every unit cell in (**Figure. S27b**). **d,** Composite mapping of the average tilt angles from **b** and **c**, providing an integrated perspective on structural tilting. **e,** Integration obtained by overlaying **d** onto **a** for a direct visual impression.

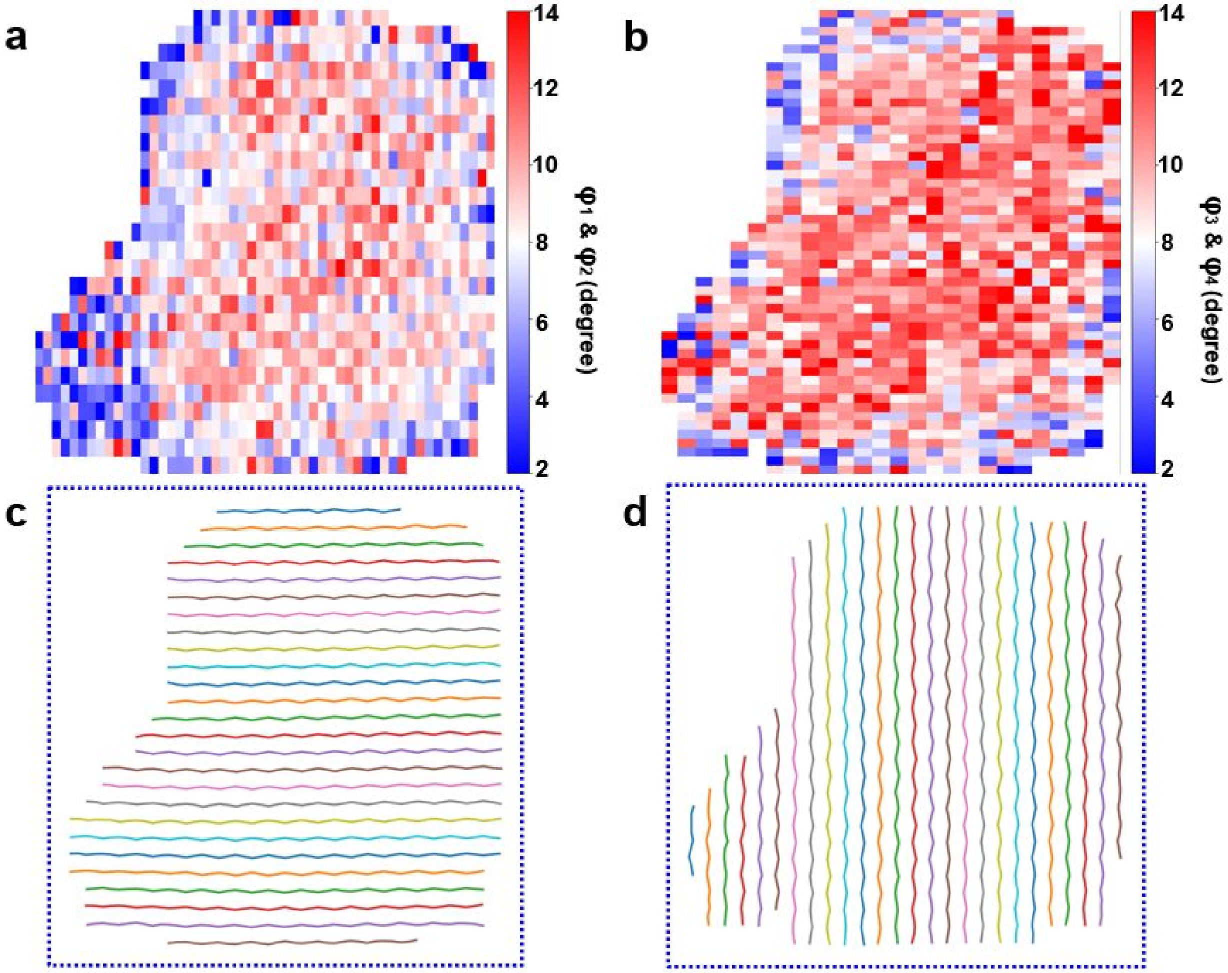

Figure S28 Tilt mappings of Pb-I in the second $CsPbI_{3-x}Br_x$ (x = 0.5) QD sample and the extracted corresponding I-Pb-I sublattice connections. a, Tilt angle mappings φ_1 and φ_2, calculated along the horizontal direction. **b,** Tilt angle mappings φ_3 and φ_4, calculated along the vertical direction. Type or paste caption here. Create a page break and paste in the Table above the caption. **c-d,** I-Pb-I lattice framework in the horizontal and vertical direction respectively.

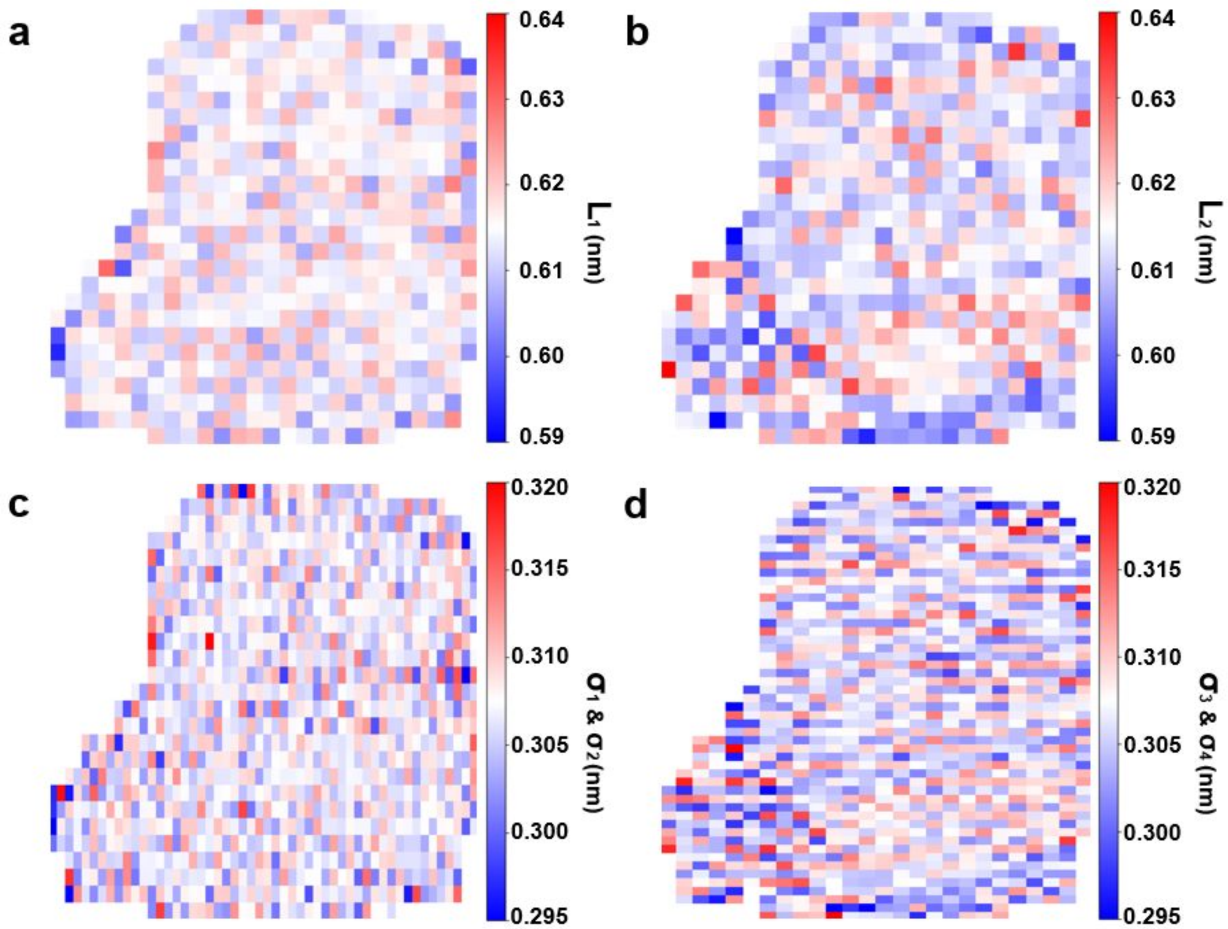

Figure S29 Bond length mappings of Pb-I in the second $CsPbI_{3-x}Br_x$ (x = 0.5) QD sample. a, Bond length mapping of L_1, obtained as the sum of σ_1 and σ_2 in every unit cell in **c**. **b,** Bond length mapping of L_2, derived as the sum of σ_3 and σ_4 in every unit cell in **d**. **c,** Bond length mapping of σ_1 and σ_2, derived from the horizontal direction. **d,** Bond length mapping of σ_3 and σ_4, derived from the vertical direction.

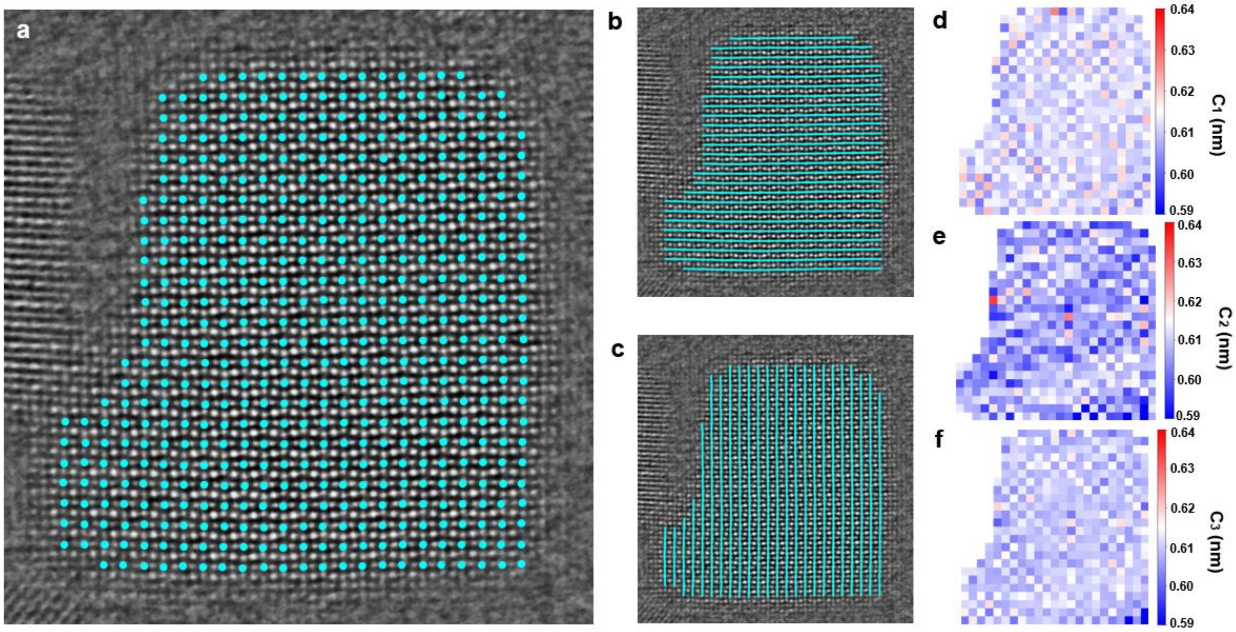

Figure S30 Characterization of Cs atoms and derived Cs-Cs sublattice and mappings in the second $CsPbI_{3-x}Br_x$ (x = 0.5) QD sample. a, Classified Cs sites, highlighted in cyan. **b,** Cs-Cs lattice periodicity resolved along the horizontal direction. **c,** Cs-Cs lattice periodicity resolved along the vertical direction. The Cs-Cs lattice spacing mapping C_1, C_2 along the **d,** horizontal and **e,** vertical directions, respectively, and their averaged mapping **f,** C_3.

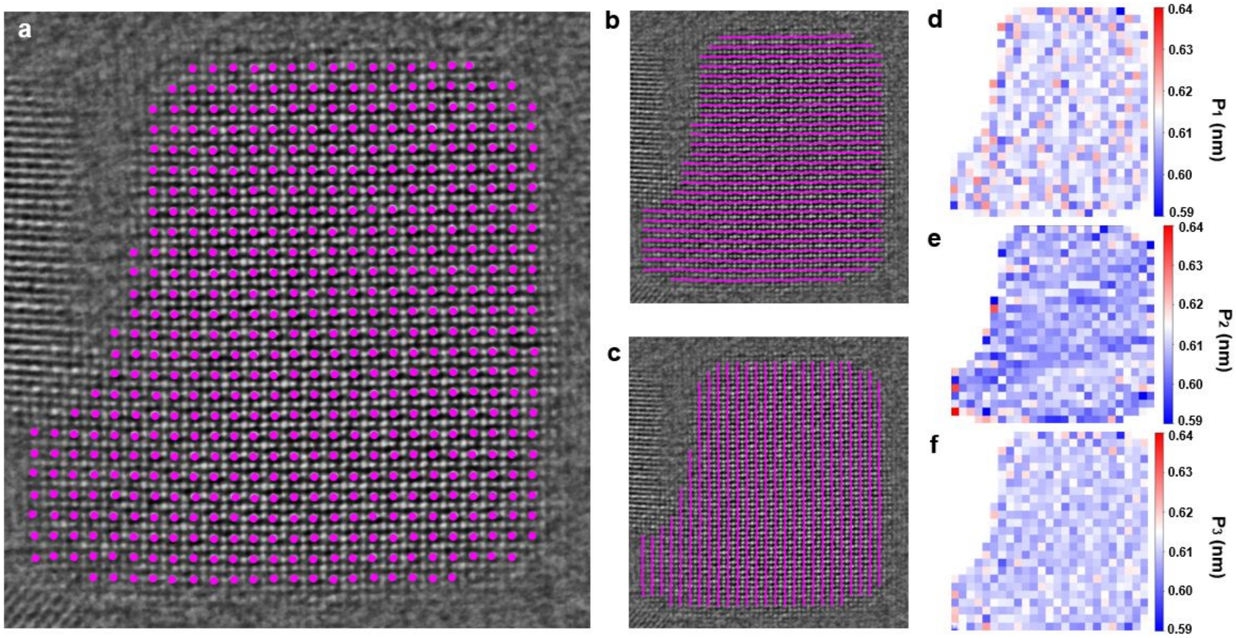

Figure S31 Characterization of Pb atoms and derived Pb-Pb sublattice and mappings in the second $CsPbI_{3-x}Br_x$ (x = 0.5) QD sample. a, Classified Pb sites, highlighted in bright purple. **b,** Pb-Pb lattice periodicity resolved along the horizontal direction. **c,** Pb-Pb lattice periodicity resolved along the vertical direction. The Pb-Pb lattice spacing mapping P_1, P_2 along the **d,** horizontal and **e,** vertical directions, respectively, and their averaged mapping **f,** P_3.

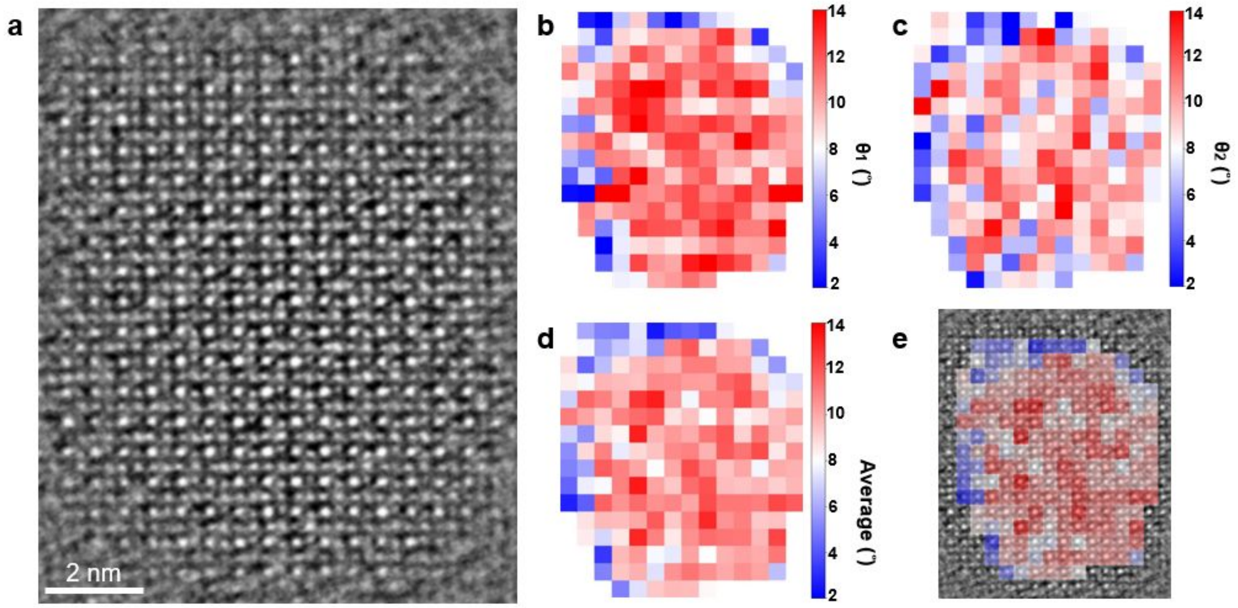

Figure S32 Characterization and tilt mappings of third $CsPbI_{3-x}Br_x$ (x = 0.5) QD sample. a, S2SRED denoised iDPC-STEM image of this sample. **b,** Mapping of the tilt angle θ_1, derived as the mean of φ_1 and φ_2 in every unit cell in (**Figure. S32a**). **c,** Mapping of the tilt angle θ_2, derived as the mean of φ_3 and φ_4 in every unit cell in (**Figure. S32b**). **d,** Composite mapping of the average tilt angles from **b** and **c**, providing an integrated perspective on structural tilting. **e,** Integration obtained by overlaying **d** onto **a** for a direct visual impression.

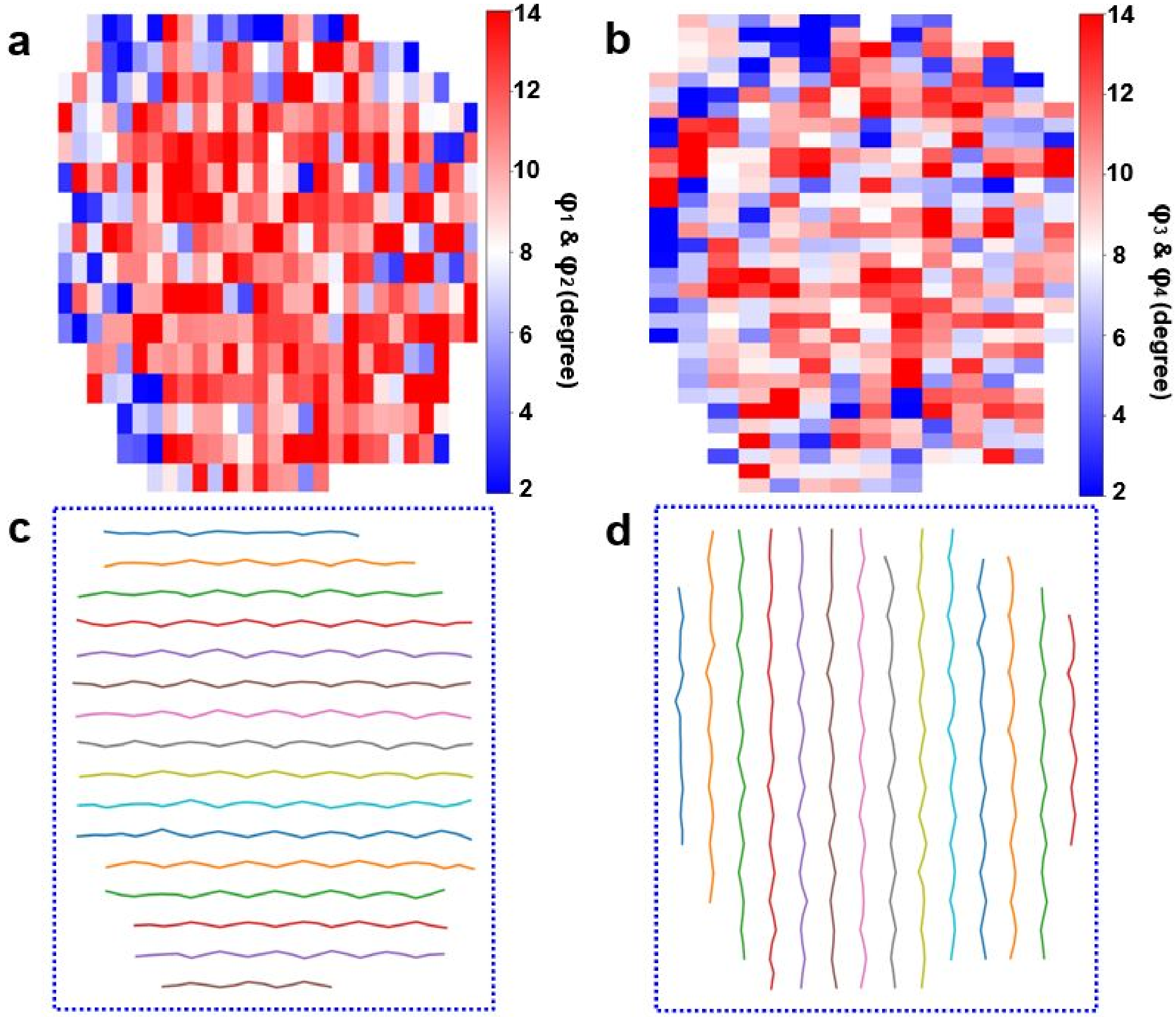

Figure S33 Tilt mappings of Pb-I in the third $CsPbI_{3-x}Br_x$ (x = 0.5) QD sample and the extracted corresponding I-Pb-I sublattice connections. a, Tilt angle mappings φ_1 and φ_2, calculated along the horizontal direction. **b,** Tilt angle mappings φ_3 and φ_4, calculated along the vertical direction. Type or paste caption here. Create a page break and paste in the Table above the caption. **c-d,** I-Pb-I lattice framework in the horizontal and vertical direction respectively.

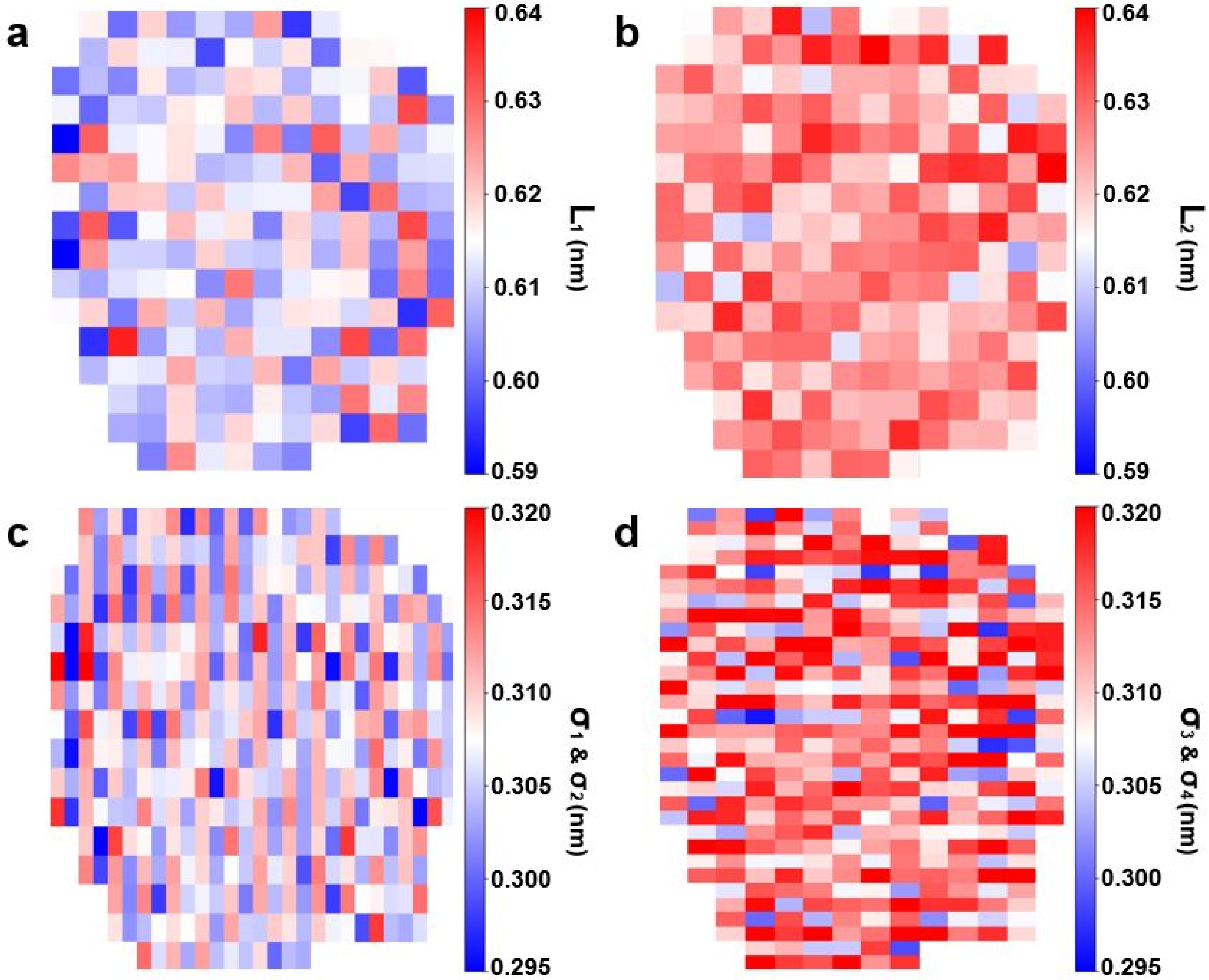

Figure S34 Bond length mappings of Pb-I in the third $CsPbI_{3-x}Br_x$ (x = 0.5) QD sample. a, Bond length mapping of L_1, obtained as the sum of σ_1 and σ_2 in every unit cell in **c**. **b,** Bond length mapping of L_2, derived as the sum of σ_3 and σ_4 in every unit cell in **d**. **c,** Bond length mapping of σ_1 and σ_2, derived from the horizontal direction. **d,** Bond length mapping of σ_3 and σ_4, derived from the vertical direction.

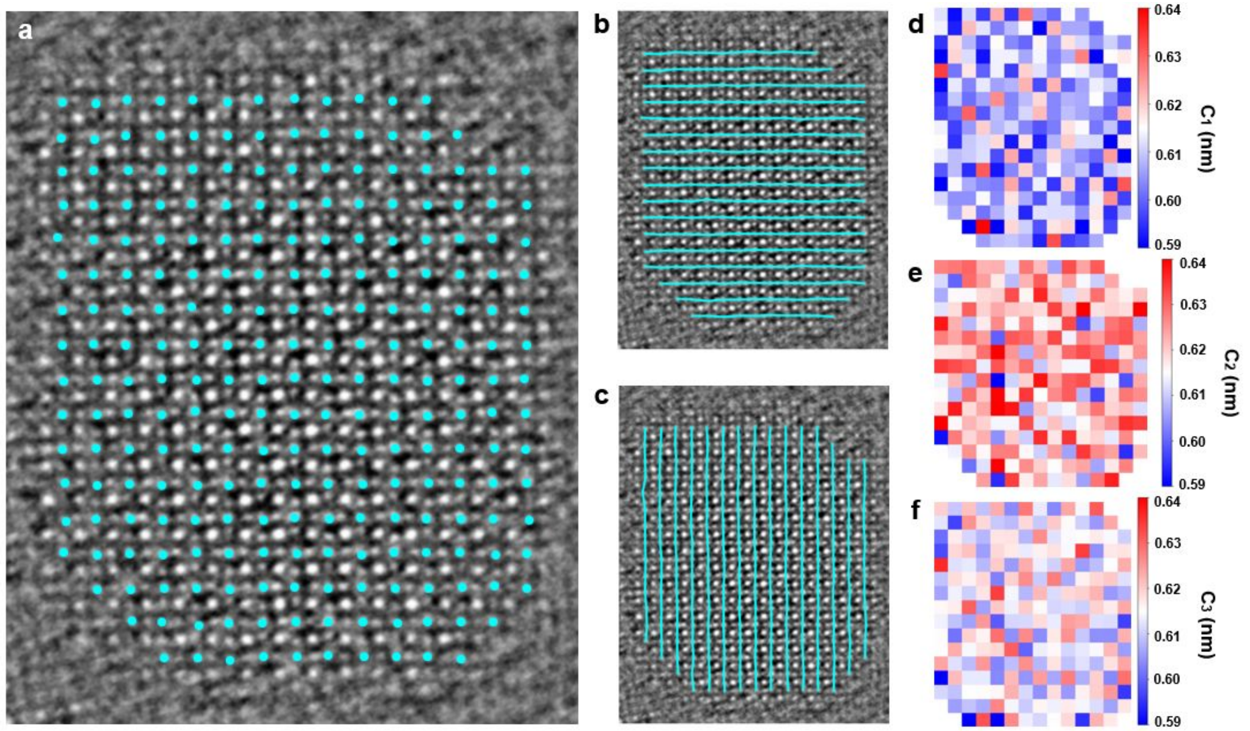

Figure S35 Characterization of Cs atoms and derived Cs-Cs sublattice and mappings in the third $CsPbI_{3-x}Br_x$ (x = 0.5) QD sample. a, Classified Cs sites, highlighted in cyan. **b,** Cs-Cs lattice periodicity resolved along the horizontal direction. **c,** Cs-Cs lattice periodicity resolved along the vertical direction. The Cs-Cs lattice spacing mapping C_1, C_2 along the **d,** horizontal and **e,** vertical directions, respectively, and their averaged mapping **f,** C_3.

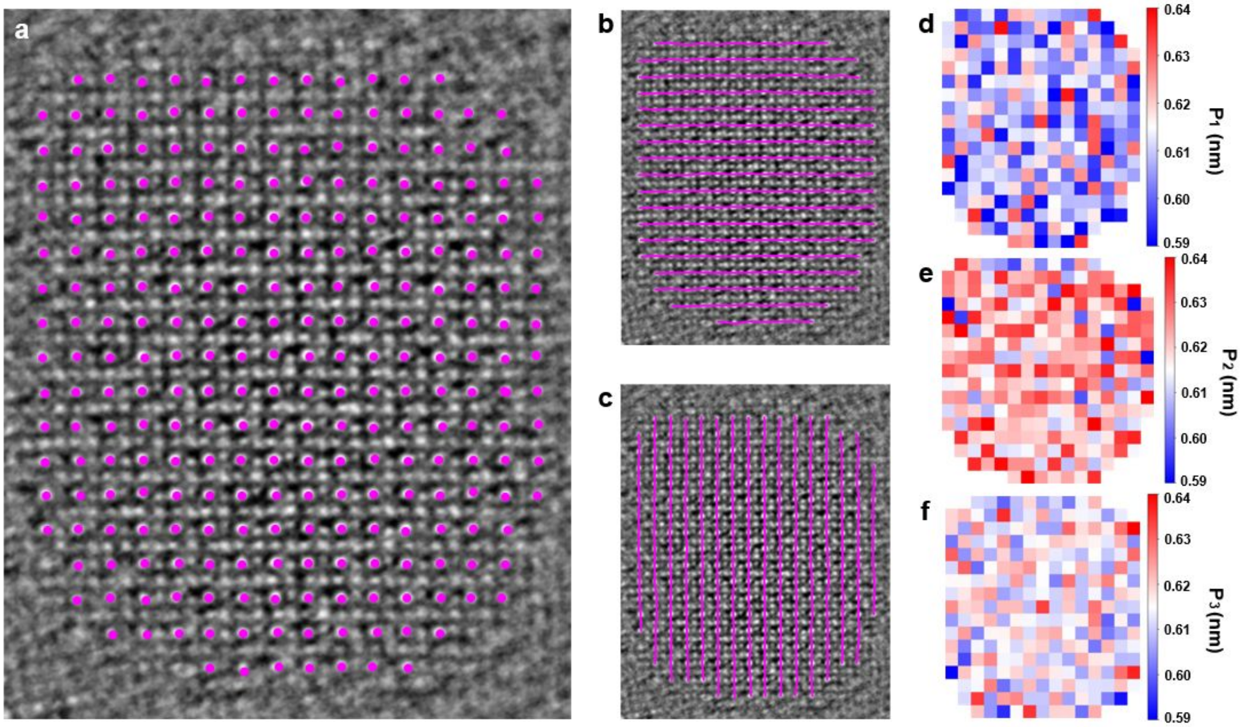

Figure S36 Characterization of Pb atoms and derived Pb-Pb sublattice and mappings in the third $CsPbI_{3-x}Br_x$ (x = 0.5) QD sample. a, Classified Pb sites, highlighted in bright purple. **b,** Pb-Pb lattice periodicity resolved along the horizontal direction. **c,** Pb-Pb lattice periodicity resolved along the vertical direction. The Pb-Pb lattice spacing mapping P_1, P_2 along the **d,** horizontal and **e,** vertical directions, respectively, and their averaged mapping **f,** P_3.

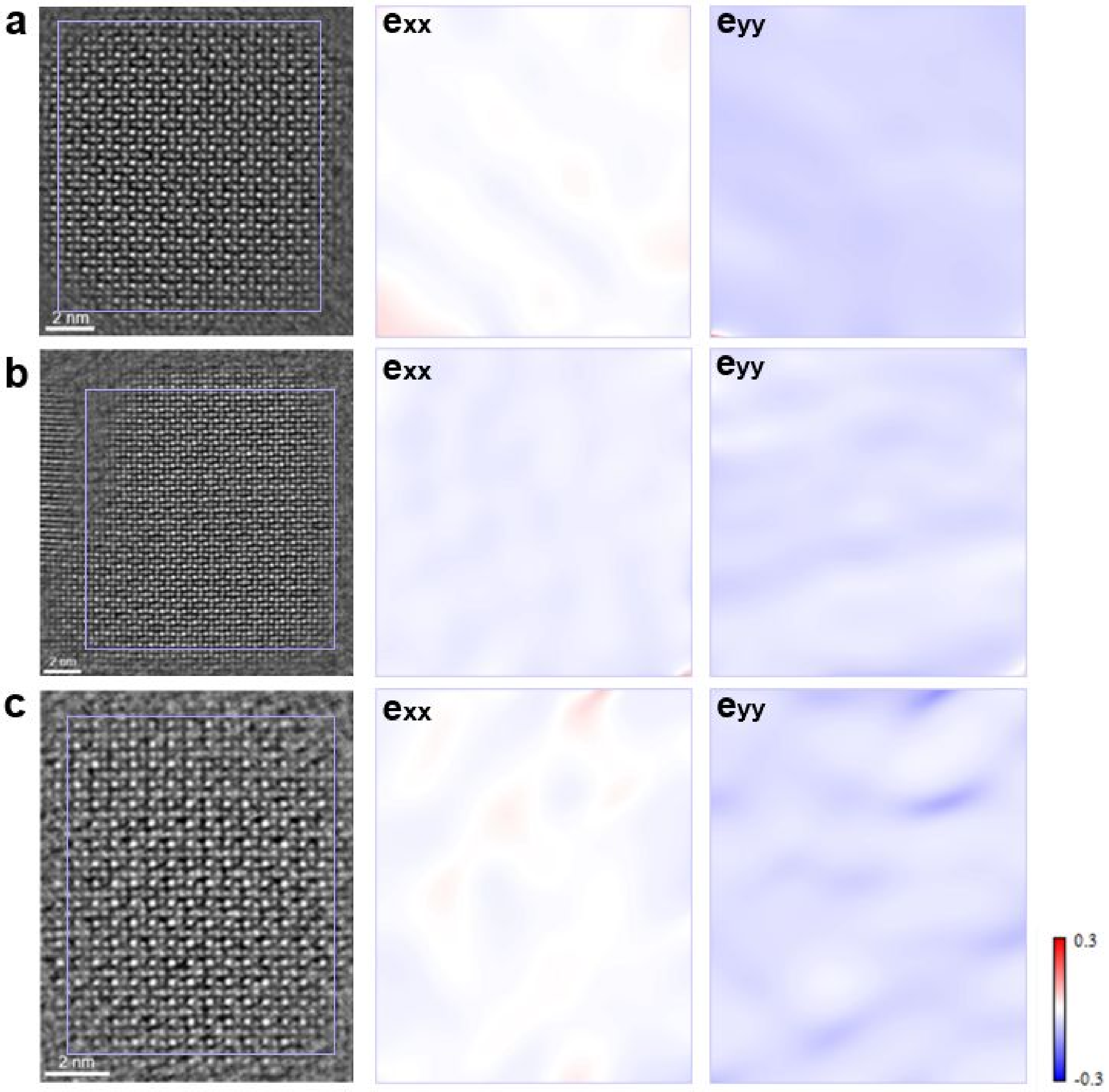

Figure S37 Geometric Phase Analysis (GPA) for the three $CsPbI_{3-x}Br_x$ (x = 0.5) samples. The GPA strain maps display a somewhat more noticeable color contrast compared to pure $CsPbI_3$, suggesting enhanced local strain variations, similar to the phenomenon in the corresponding Cs-Cs and Pb-Pb maps.

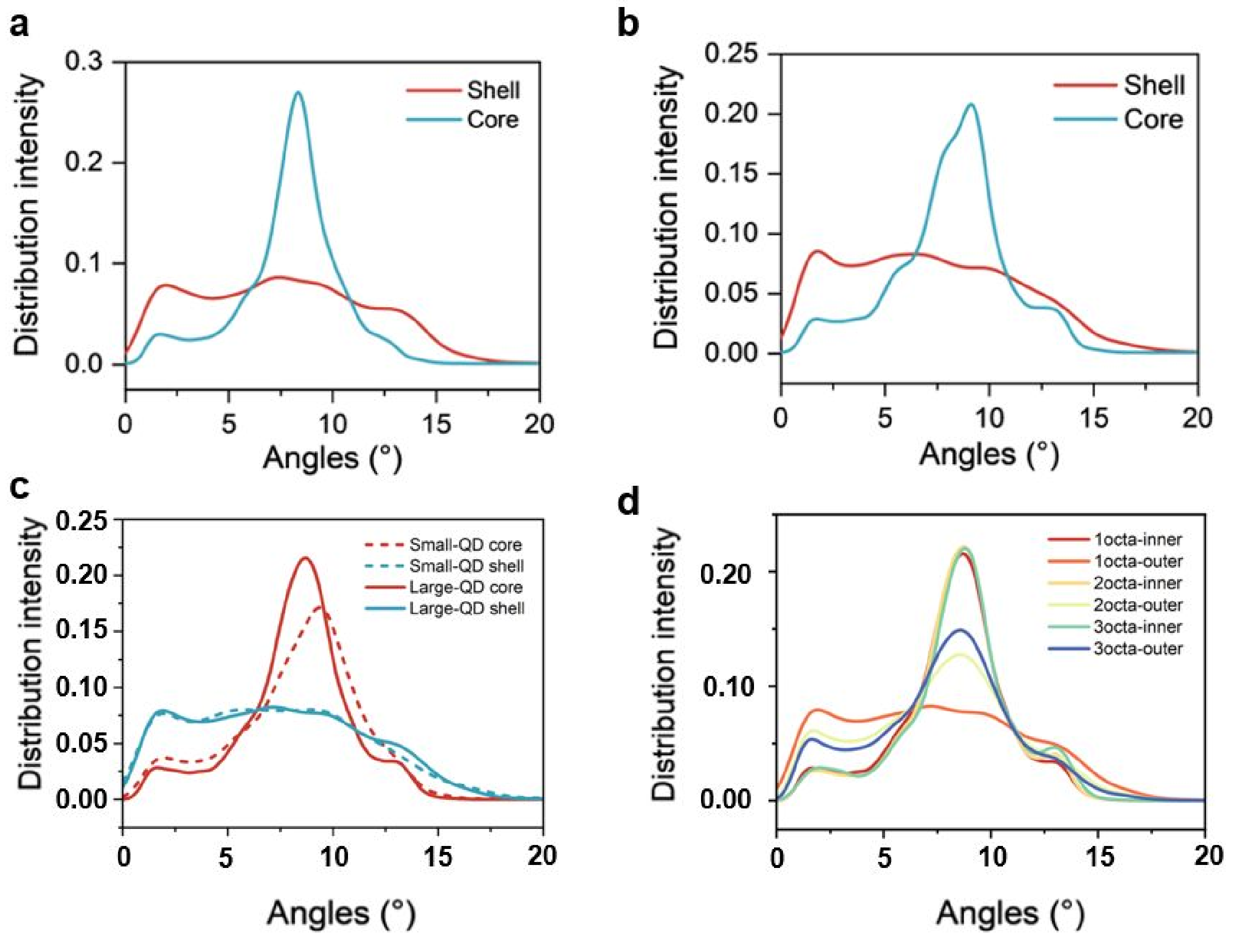

Figure S38 MD simulation results of Pb-I bond angles distribution in $CsPbI_3$ QDs. Distribution of Pb-I bond distortion angles in both shell and core regions from **a,** hidden XZ projection plane and **b,** hidden YZ projection plane. **c,** Distribution of Pb-I bond distortion angles in both shell and core regions in small (4 nm) and large (9.5 nm) $CsPbI_3$. The smaller QD simulation clearly exhibits a core-shell differentiation. The core region (red dashed line) maintains a high tilting angle distribution peaking around 9.5°, while the shell region (blue dashed line) shows a significantly broader distribution with much lower tilt angles (peaking around 2° and 8°), indicating suppressed octahedral tilting at the surface. **d,** Distribution of Pb-I bond distortion angles in both shell and core regions as different surface regions. For 1-layer definition (1octa), the distinction between the shell and the core is most pronounced here. The 1octa-outer curve (orange line) shows a broad, flattened distribution, indicating significant structural relaxation at the immediate surface. For 2-layer and 3-layer definitions: As we expand the shell definition to include the second (2octa) and third (3octa) layers, the outer distribution curves begin to develop a prominent peak around 9°, which is characteristic of the bulk-like inner core. This analysis demonstrates that the unique surface relaxation behavior (significantly reduced tilting) is predominantly confined to the outermost layer of octahedra. Including deeper layers effectively mixes bulk properties into the shell statistics.

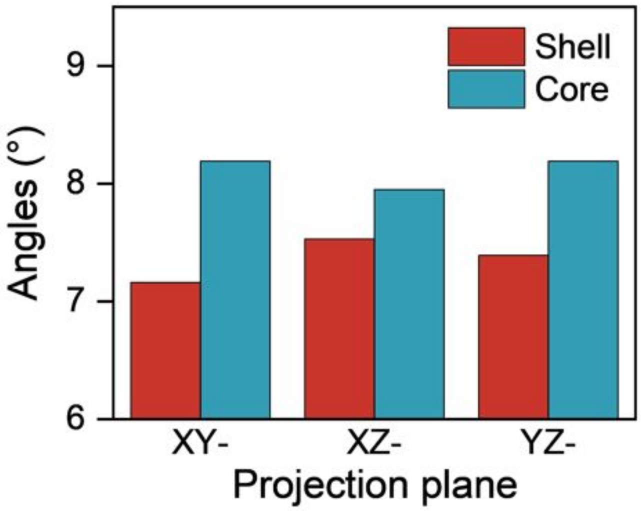

Figure S39 MD simulation results of the average distortion angles in the shell and core regions projected onto different planes in $CsPbI_3$.

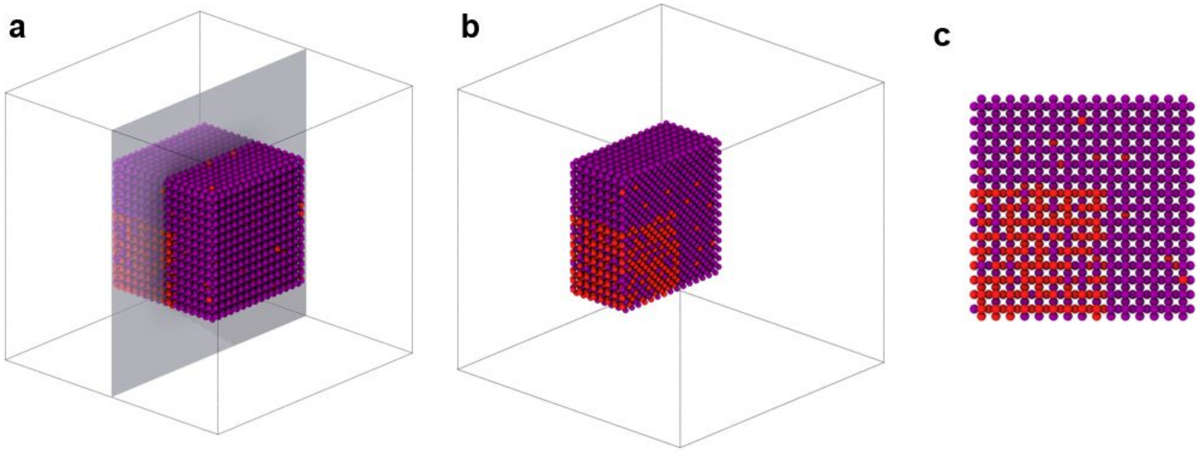

Figure S40 Structural model of $CsPbI_{3-x}Br_x$ (x = 0.5) perovskite QD. a, 3D atomic rendering of the perovskite nanocube, with a translucent slice plane highlighting the internal halide distribution. **b,** Isolated view of the cube emphasizing the substitutional incorporation of Br (red) within the I (purple) sublattice, predominantly concentrated in the lower domain. **c,** Planar projection of the halide lattice, illustrating the spatial segregation of Br atoms into localized regions within the otherwise I-rich framework.

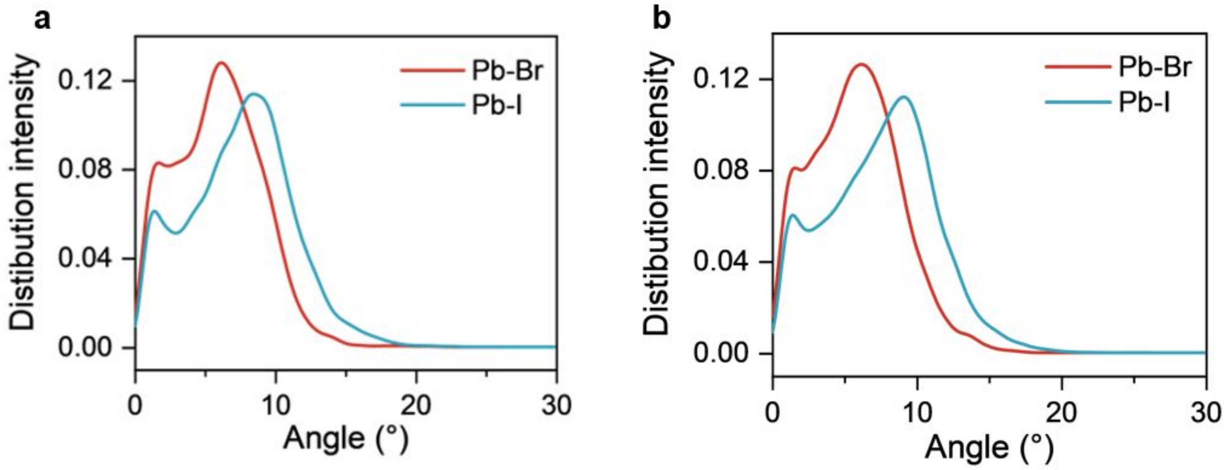

Figure S41 MD simulation results of Pb-X (X = I/Br) bond angles distribution in $CsPbI_{3-x}Br_x$ (x = 0.5) QDs. Distribution of Pb-X bond distortion angles from **a,** hidden XZ projection plane and **b,** hidden YZ projection plane.

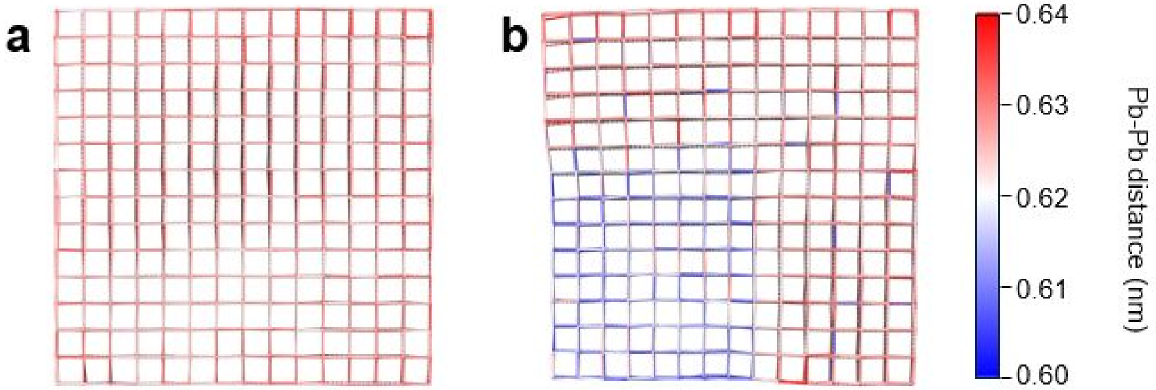

Figure S42 Interatomic Pb-Pb distance analysis in $CsPbI_3$ and $CsPbI_{3-x}Br_x$ (x = 0.5) perovskite QDs. a, Pb-Pb distance mapping in $CsPbI_3$. **b,** Pb-Pb distance mapping in $CsPbI_{3-x}Br_x$ (x = 0.5).

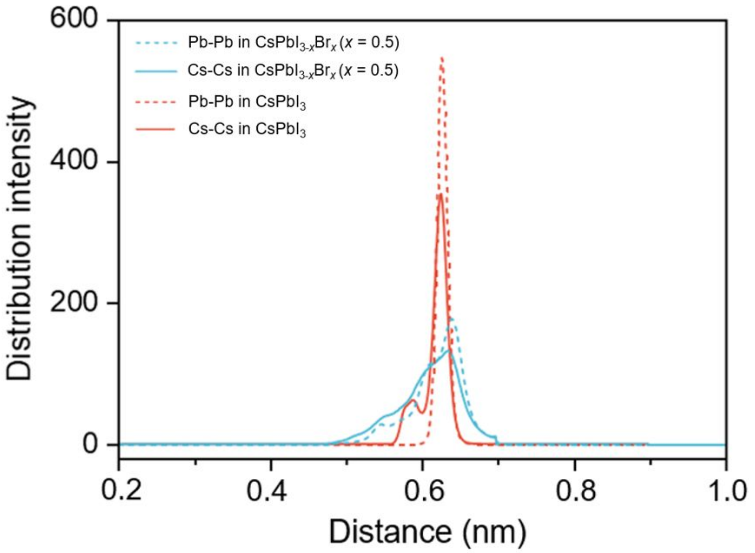

Figure S43 Statistical distribution of Pb-Pb and Cs-Cs distances in $CsPbI_3$ and $CsPbI_{3-x}Br_x$ (x = 0.5), showing the effect of halide substitution on the local structural environment.

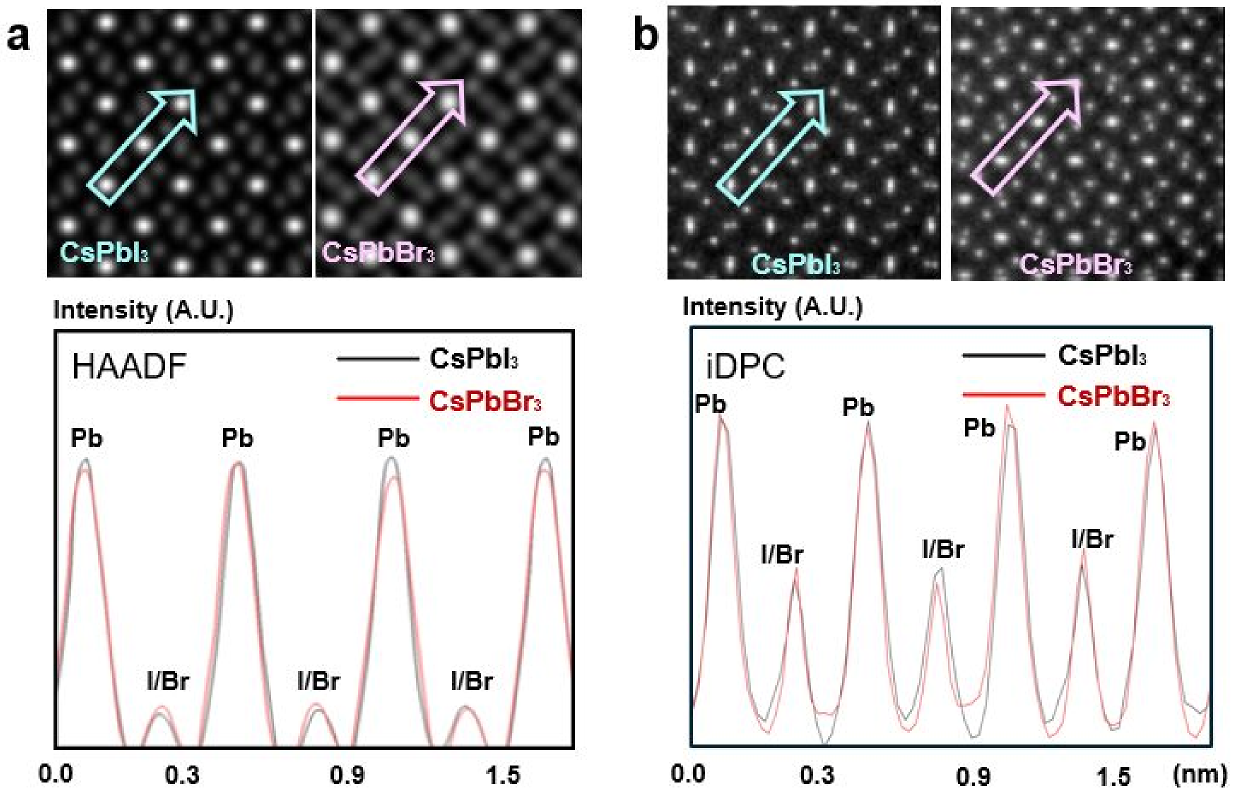

Figure S44 Simulated HAADF- and iDPC-STEM images with corresponding intensity line profiles of $CsPbI_3$ and $CsPbBr_3$. a, HAADF-STEM simulations of the two perovskites, $CsPbI_3$ (left) and $CsPbBr_3$ (right), with cyan and magenta arrows marking the crystallographic direction along which the intensity line profile was extracted. The line-profile plot beneath shows the alternating Pb and I/Br columns whose curves for the two compounds almost completely overlap, indicating nearly identical HAADF contrast at the halide sites. **b,** iDPC-STEM simulations of the same structures with the same profile-extraction direction highlighted by arrows. The corresponding iDPC line profile likewise exhibits closely aligned Pb and I/Br peaks for $CsPbI_3$ (black) and $CsPbBr_3$ (red), with only a slight deviation in amplitude yet nearly identical peak positions at the halide columns. These results demonstrate that Br-for-I substitution produces minimal contrast change in either imaging mode, emphasizing the difficulty of distinguishing Br segregation directly from raw intensity.
